# Subtype-associated gut microbial taxa and their clinical correlates in acute ischemic stroke: comparison of large-artery atherosclerosis and small-vessel occlusion

**DOI:** 10.3389/fmicb.2026.1867057

**Published:** 2026-09-11

**Authors:** Songyi Park, Jonghee Choi, Ali Sadra, Sang-Hwa Lee, Jong-Hee Sohn

**Affiliations:** 1 Institute of New Frontier Research Team, College of Medicine, Hallym University, Chuncheon, Republic of Korea; 2 Department of Neurology, Chuncheon Sacred Heart Hospital, Hallym University College of Medicine, Chuncheon, Republic of Korea

**Keywords:** gut-brain axis, ischemic stroke, large-artery atherosclerosis, microbiome, small-vessel occlusion

## Abstract

**Objective:**

The gut microbiome gets altered during ischemic stroke (IS); however, whether distinct microbial signatures characterize specific IS subtypes, such as large-artery atherosclerosis (LAA) and small-vessel occlusion (SVO), and their relation to subtype-specific clinical features, remains unclear. This study aimed at comparing gut microbiome profiles among patients with LAA, patients with SVO, and healthy controls (HCs); further, the associations between subtype-enriched microbial taxa and clinical parameters were examined.

**Methods:**

We compared gut microbiome profiles and clinical associations among patients with LAA, patients with SVO, and HCs (*n* = 50 per group), using 16S rRNA sequencing to analyze microbial diversity and taxonomic composition. Correlation analyses were then performed between clinical parameters and the identified differential taxa.

**Results:**

Linear discriminant analysis effect size analysis showed that, compared with HCs, patients with IS had reduced alpha diversity and distinct beta diversity. No significant differences were observed between patients with LAA and SVO in alpha diversity (all indices, *p* > 0.05) or beta diversity (*R*^2^ = 0.009, *p* = 0.595). Taxa enriched in HCs (*Agathobaculum, Holdemanella*, and *Prevotella*) were negatively associated with age, D-dimer levels, and modified Rankin Scale scores. LAA-enriched taxa (*Desulfovibrio* and *Eubacterium*) were positively correlated with Fazekas scale scores. SVO-enriched taxa (*Catenibacterium, Collinsella, Megamonas, Sellimonas*, and *Weissella*) were positively correlated with hemoglobin A1c, body mass index, and National Institutes of Health Stroke Scale scores.

**Conclusion:**

Although overall microbial diversity did not differ between patients with LAA and SVO, subtype-enriched taxa were associated with distinct clinical features. These findings support the presence of subtype-specific microbial patterns that may reflect different pathophysiological processes; however, causal relationships remain to be established.

## 1 Introduction

Ischemic stroke (IS) occurs when cerebral blood flow is interrupted by vessel occlusion due to thrombosis or atherosclerosis. In contrast, hemorrhagic stroke results from rupture of a cerebral blood vessel. Although all stroke types cause brain tissue injury and neurological impairment, each has distinct clinical characteristics. IS accounts for approximately two-thirds of all stroke cases and is further classified into three etiological subtypes according to the underlying mechanism of vessel occlusion: large-artery atherosclerosis (LAA), small-vessel occlusion (SVO), and cardioembolism (CE; Caplan, 2000).

LAA and SVO are associated with systemic vascular conditions, including hypertension and atherosclerosis. Conversely, CE is caused by emboli originating from the heart and is frequently related to atrial fibrillation (Adams et al., 1993). In CE, cerebral vessels may be relatively healthy, but embolic occlusion can lead to severe clinical outcomes. In contrast, LAA and SVO are characterized by chronic inflammation, endothelial dysfunction, and impaired metabolic regulation associated with their underlying vascular pathology.

In this study, we focused on the differentiating microbiome-related factors in LAA and SVO because their etiologies are closely related to cerebral vessel health. Comparing these two subtypes is particularly relevant because their systemic and vascular features are influenced by the gut microbiome through multiple mechanisms, including immune modulation, microbial metabolite production, and neuroendocrine pathways (Cryan et al., 2019; Loh et al., 2024; Zhuang et al., 2024). The gut microbiome has also been implicated in other neurodegenerative conditions, including Parkinson's disease and Alzheimer's disease (Chandra et al., 2023; Pluta et al., 2021; Romano et al., 2021; Seo and Holtzman, 2024; Tan et al., 2022; Zhu et al., 2022), highlighting the broad relevance of gut-brain interactions across neurological disorders.

Several clinical studies have reported reduced gut microbiome diversity and altered microbial composition in patients with IS compared with healthy individuals. A recent study highlighted the bidirectional relationship between brain injury and gut dysbiosis, showing that IS can disrupt intestinal homeostasis, and microbial metabolites may influence poststroke recovery (Pluta et al., 2021). Another study has emphasized the role of microbiome in modulating established stroke risk factors, such as hypertension, hyperglycemia, and atherosclerosis, and discussed microbiota-targeted interventions as potential preventive or therapeutic strategies (Wang et al., 2022). In addition, numerous studies have sought to identify microbial taxa that are increased in patients with IS compared with healthy controls (HCs), but the results have been inconsistent (Chen et al., 2024; Hu et al., 2022; Lee et al., 2021; Yu et al., 2024). Beyond taxonomic comparisons, recent studies have identified gut microbiome-derived metabolites, including short-chain fatty acids (SCFAs), trimethylamine N-oxide (TMAO), and phenylacetylglutamine (PAGln), that are closely linked to IS pathogenesis (Henry et al., 2021; Li et al., 2024; Yu et al., 2021).

Recent research has begun to examine the relationship between gut microbes and IS subtypes, including LAA and SVO, because the gut microbiome may contribute differently to their pathogenesis. A recent large-scale Mendelian randomization analysis identified specific gut microbial taxa associated with different IS subtypes, including LAA and SVO, suggesting differential microbial contributions to pathogenesis (Meng et al., 2023). However, comparative analyses of IS subtypes remain scarce. Although the gut microbiome has been linked to various clinical and demographic parameters related to IS (Huang et al., 2023; Wang et al., 2022), few studies have investigated the associations between subtype-specific microbial taxa and these parameters (He P. et al., 2024). Direct head-to-head comparisons of gut microbiome profiles between LAA and SVO subtypes are scarce; therefore, it is unclear whether subtype-specific microbial signatures exist and how they relate to the distinct clinical features of each subtype.

To address these limitations, we compared the gut microbiome profiles among Korean patients with LAA, Korean patients with SVO, and HCs. Using 16S rRNA sequencing and differential taxonomic analysis, we investigated whether distinct microbial signatures characterized the LAA and SVO subtypes of IS and whether these signatures were associated with specific clinical parameters. Our findings indicate that specific taxa are differentially associated with selected clinical features, offering potential mechanistic and therapeutic insights. By identifying subtype-associated microbial patterns in LAA and SVO, we aimed to clarify how these differences reflect the divergent pathophysiology of each IS subtype. In addition, we evaluated whether specific bacterial taxa, in combination with relevant clinical parameters, could serve as potential indicators of IS predisposition in a hospital-based cohort.

## 2 Materials and methods

### 2.1 Study participants

Patients were prospectively and consecutively enrolled from among those admitted to the Department of Neurology at Chuncheon Sacred Heart Hospital. The inclusion criteria were as follows: (1) age ≥19 years; (2) admission with acute IS within 1 week of symptom onset; (3) diagnosis confirmed by clinical evaluation and computed tomography/magnetic resonance imaging (MRI) assessed by board-certified neurologists; and (4) classification as LAA or SVO according to the TOAST classification (Adams et al., 1993).

One hundred patients were included in the study, comprising 50 patients with LAA and 50 with SVO. The HC group was age- and sex-matched to the mean demographic characteristics of the combined LAA and SVO patient groups. The exclusion criteria were as follows: (1) diagnosis of transient ischemic attack (TIA) rather than completed IS; (2) IS subtypes other than LAA or SVO (e.g., cardioembolism, undetermined etiology); (3) refusal to participate in the study or inability to provide informed consent; (4) antibiotic or probiotic use within the previous month; (5) active malignancy, chronic kidney disease requiring hemodialysis or peritoneal dialysis, decompensated liver cirrhosis, inflammatory bowel disease, or other serious systemic/gastrointestinal comorbidities known to independently alter gut microbiota composition; or (6) insufficient stool sample volume or sequencing quality control (QC) failure identified after sample collection.

De-identified gut microbiome data from HCs were obtained from CJ Bioscience (Seoul, Republic of Korea) and analyzed in parallel with patient samples. Patients were enrolled consecutively until the pre-specified sample size of 50 per subtype was reached. As sample collection and clinical data capture was performed only once (during the index hospitalization), no longitudinal follow-up was performed and no loss to follow-up occurred. Five samples were subsequently excluded owing to insufficient stool volume or sequencing QC failure (LAA, *n* = 2; SVO, *n* = 3); these sample-level exclusions were applied prior to analysis, and no imputation was used to replace them. A participant flow diagram is provided as Supplementary Figure 1.

### 2.2 Clinical data and stool sample collection

Clinical and demographic data were collected from all 100 patients. Demographic parameters included age, body mass index (BMI), height, sex, and weight. Clinical parameters included laboratory blood markers, including hemoglobin, hematocrit, prothrombin time (PT), D-dimer, glucose levels, blood urea nitrogen (BUN), and white blood cell (WBC) count; systolic and diastolic blood pressure; the National Institutes of Health Stroke Scale score (NIHSS); the modified Rankin Scale score (mRS) at baseline and discharge; and Fazekas scale scores from MRI. The Fazekas scale was evaluated using the standard 0–3 visual grading system on axial fluid-attenuated inversion recovery (FLAIR) MRI. White matter hyperintensities were semi-automatically quantified using Neurophet AQUA (Neurophet, Seoul, Republic of Korea), a deep learning-based software program for volumetric analysis of brain MRI scans. Patient stool samples were collected during the acute phase of IS within 1 week of stroke onset. Samples were collected using a spatula attached to a stool collection container and stored at −80 C until analysis. Approximately 1–3 g of fresh stool was collected at a single time point. HC samples were obtained using a standard stool collection procedure from the Healthy Korean Microbiome Project (Oh et al., 2022). To ensure comparability, HCs were randomly selected and matched to the patient group by age range and sex. Stool-based microbiome samples were obtained from both groups. Differences in sample collection context between groups are acknowledged as a potential source of systematic variation, namely acute hospitalized patients vs. community-based healthy individuals.

### 2.3 Standard protocol approvals, registrations, and patient consents

De-identified gut microbiome data from HCs was obtained from the Healthy Korean Microbiome Project (CJ Bioscience) through a data-use agreement. Samples were collected from Korean individuals in three independent studies conducted between 2016 and 2020. Samples collected between 2016 and 2017 were obtained through a collaborative project between ChunLab, Inc. and Samsung Medical Center, which was approved by the Samsung Medical Center Institutional Review Board (IRB No. 2016-06-140). Use of these data for research purposes was approved by the IRB of Chuncheon Sacred Heart Hospital. This study was also approved by the Institutional Review Board of Chuncheon Sacred Heart Hospital, Hallym University Medical Center (IRB No. 2022-01-028). Each participant provided written informed consent before participation.

### 2.4 DNA extraction and 16S rRNA sequencing

Bacterial DNA was extracted using the Maxwell RSC PureFood GMO Authentication Kit (Promega, Madison, WI, USA). The V3–V4 regions of the 16S rRNA gene were amplified using primers 341F 5′-CCTACGGGNGGCWGCAG) and 805R 5′-GACTACHVGGGTATCTAATCC). PCR was performed with initial denaturation at 95 C for 3 min, followed by 25 cycles of 95 C, 55 C, and 72 C for 30 s each, and a final extension at 72 C for 5 min. Amplification was confirmed by electrophoresis on a 1% agarose gel using the Gel Doc system (Bio-Rad, Hercules, CA, USA). Amplicons were purified using a bead-based method, followed by size selection with the ProNex Size-Selective Purification System (Promega) to remove non-target fragments. DNA concentration was measured using the QuantiFluor dsDNA System (Promega), and libraries were prepared by pooling samples at equimolar concentrations. Sequencing was performed on an Illumina MiSeq platform (Illumina, San Diego, CA, USA) by CJ Bioscience. To minimize technical variability, all stool samples, including those from HCs obtained from the Healthy Korean Microbiome Project (CJ Bioscience), were processed under identical conditions. These conditions included the use of the same DNA extraction kit (Maxwell RSC PureFood GMO and Authentication Kit), V3–V4 primer set, PCR amplification protocol, sequencing platform (Illumina MiSeq), sequencing facility (CJ Bioscience), and bioinformatics pipeline (DADA2/QIIME2, SILVA release 138). A small batch-effect assessment using PERMANOVA on run-level grouping variables showed no significant batch-related stratification (*p* > 0.05).

### 2.5 Sequence processing and quality control

Illumina paired-end sequencing reads were separated by sample using barcode information, and primer sequences were removed using Cutadapt (Martin, 2011). Low-quality bases (*Q* ≤ 20) were trimmed, and reads were filtered, merged, and denoised using the DADA2 pipeline (Callahan et al., 2016). Chimeric sequences were then removed, and amplicon sequence variants (ASVs) were generated using the DADA2 plugin in QIIME2 (total ASV count: 6,678; version 2021.11) (Supplementary Figure 2, Supplementary Tables 1–3, and Supplementary Data) (Bolyen et al., 2019).

### 2.6 Microbial diversity and differential abundance analysis

Alpha and beta diversity analyses were performed to evaluate gut microbiome genus structure at the genus level. Alpha diversity was calculated using the Chao 1, Shannon, Faith's phylogenetic diversity (PD), and Observed Features indices to evaluate microbial richness and diversity. Beta diversity was assessed using the Bray–Curtis dissimilarity index and visualized using principal coordinates analysis (PCoA). Statistical significance was evaluated using permutational multivariate analysis of variance (PERMANOVA). Linear discriminant analysis effect size (LEfSe) analysis was conducted to identify differentially abundant taxa across IS subtypes using linear discriminant analysis (LDA) score thresholds of 2.0 or 3.0, depending on the comparison. Alpha diversity was calculated using rarefied ASV-level feature tables with a rarefaction depth of 28,495 reads per sample. Beta diversity results were confirmed to be consistent using both rarefied and unrarefied tables. Taxonomic classification was performed using the SILVA rRNA database (release 138). PERMANOVA batch-effect assessment of sequencing-run grouping showed no significant stratification (*p* > 0.05). We applied an LDA score threshold of ≥3.0 for three-group comparisons (HC vs. LAA vs. SVO) and ≥2.0 for pairwise comparisons (LAA vs. SVO), consistent with standard LEfSe practice. LEfSe uses an internal nonparametric framework combined with LDA score thresholds as an effect size filter to reduce false positives from taxonomically correlated features. Benjamini–Hochberg false discovery rate (FDR) correction was applied to all Spearman correlation analyses. FDR correction was not applied separately to the LEfSe output because the LDA threshold served as an effect-size filtering step.

### 2.7 Microbiome-based clustering and ordination

Unsupervised clustering was performed on genus-level microbiome profiles to stratify patients with IS independently of conventional IS subtypes. The optimal number of microbiome-based clusters was determined using silhouette analysis. Differentially abundant taxa between the identified clusters were then determined using LEfSe with an LDA threshold of 2.0. To evaluate associations between the microbiome-derived clusters and clinical parameters, distance-based redundancy analysis (dbRDA) was performed using genus-level profiles; *envfit* was applied to fit clinical parameters as vectors onto the ordination space and to test their significance.

### 2.8 Microbial association with clinical parameters

Associations between gut microbial taxa and clinical parameters were assessed within the IS cohort using Spearman's rank correlation coefficient (Zar, 2005). Correlations were assessed between genus-level microbial abundances and clinical parameters, including demographic variables, laboratory markers, and neurological and radiological assessments such as the NIHSS, mRS, and the Fazekas scale. Statistical significance was evaluated using *p* values for correlations between genus-level microbial abundance and clinical parameters, followed by multiple-testing correction using the FDR to obtain FDR-adjusted *q* values. Correlation results were visualized in a heatmap with color gradients representing the strength and direction of correlations, and statistical significance was indicated by *p* values. Separately, correlations evaluated using FDR-adjusted *q* values were visualized using radial plots, in which fill color encoded the Spearman correlation coefficient and radial length was scaled by –log10(*q*). Based on LEfSe group comparisons, each genus was classified as HC-, LAA-, or SVO-dominant. Correlation results were then presented for the dominant groups. All data visualizations were generated using in-house R scripts together with the ggplot2 R package (version 3.3.5; CJ Bioscience).

### 2.9 Statistical analysis and data visualization

Data are presented as the mean ± standard error of the mean (SEM), unless otherwise specified. Statistical analyses were performed using GraphPad Prism (version 8; GraphPad Software, San Diego, CA, USA), SPSS Statistics (version 30.0; IBM, Armonk, NY), and R (version 4.4.2; R Foundation for Statistical Computing, Vienna, Austria). Clinical and demographic parameters were analyzed using parametric tests [Student's t-test or one-way analysis of variance (ANOVA)] or nonparametric tests (Mann–Whitney U test or Kruskal–Wallis test), as appropriate. Nonparametric tests, including the Wilcoxon rank-sum test and Kruskal–Wallis test, were used for microbiome diversity indices. In all analyses, *p* < 0.05 was considered statistically significant. All correlation analyses are hypothesis-generating. Multiple testing was addressed using the Benjamini–Hochberg FDR correction, and associations with *q* < 0.05 were considered as primary findings.

## 3 Results

### 3.1 Participant characteristics and sequencing overview

**Figure 1:**
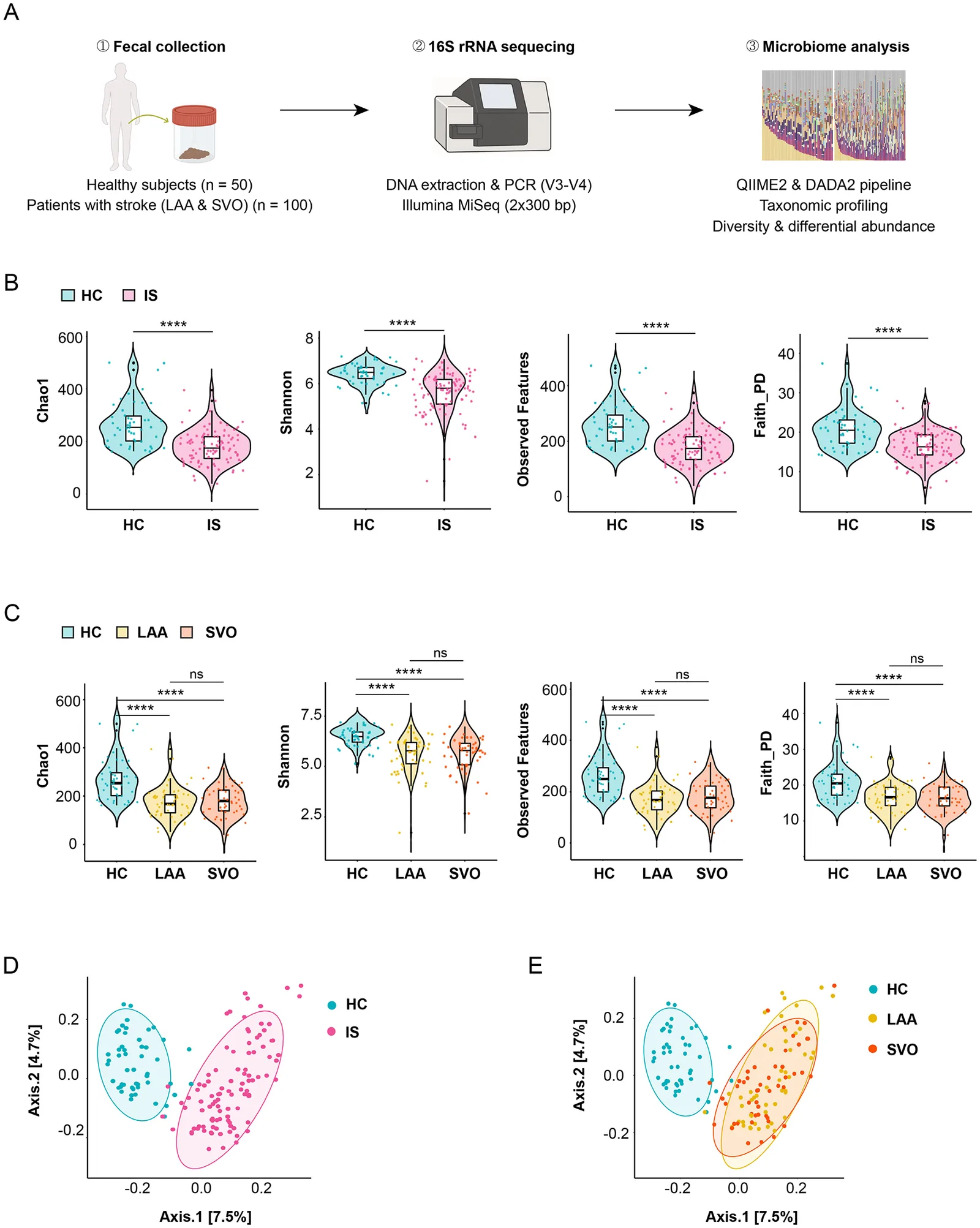
Study design and diversity analysis of the gut microbiota in IS and HC. **(A)** Stool samples from IS patients (LAA, *n* = 50; SVO, *n* = 50) and HC (*n* = 50) were profiled by 16S rRNA sequencing. **(B)** Alpha diversity indices (Chao1, Shannon, Observed Features, Faith_PD) were significantly lower in IS (pink) compared to HC (blue; *p* < 0.0001). **(C)** Both LAA (yellow) and SVO (orange) showed reduced diversity compared to HC, with no difference between the subtypes (*p* < 0.0001). **(D)** Bray–Curtis PCoA showed clear separation between HC and IS (*p* < 0.001). **(E)** HC was also distinct from both LAA and SVO (both *p* < 0.001), while LAA and SVO did not show a significant difference (*p* = 0.595). Images were created using BioRender.com. IS, ischemic stroke; HC, healthy control; LAA, large-artery atherosclerosis; SVO, small-vessel occlusion; PCoA, principal component analysis.

A total of 100 patients with IS were enrolled in this study, including 50 patients with LAA and 50 patients with SVO (Figure 1A). Among these patients, 63% were male (*n* = 63) and 37% were female (*n* = 37), with a mean age of 69.41 ± 11.73 years. Age did not differ significantly between the LAA and SVO groups (70.48 ± 11.06 vs. 68.34 ± 12.38 years, *p* = 0.364). The HC group (*n* = 50) was matched to the IS cohort by age and sex; it comprised 74% male participants (*n* = 37) and 26% female participants (*n* = 13), with a mean age of 64.76 ± 7.84 years. Detailed demographic and clinical characteristics of the patients with IS are presented in Table 1. However, individual-level clinical metadata were unavailable for HCs, limiting direct cross-group comparisons of microbiome-clinical associations. We surveyed the use of medications that could potentially influence gut microbiome composition in all patients with IS and summarized the results (Table 1). Medication profiles did not differ significantly between the LAA and SVO groups. However, medication data were unavailable for HCs; therefore, cross-group adjustment for medication effects was not performed. A total of 4,694,358 raw merged reads were obtained from 100 fecal samples from patients with IS, corresponding to 28,495–69,012 reads per sample (mean: 46,943 ± 10,063). After quality control, 3,990,230 clean reads were retained for downstream analyses (Supplementary Figure 2).

**Table 1:** Clinical and demographic characteristics of IS.

Group	IS(*n* = 100)	LAA(*n* = 50)	SVO(*n* = 50)	*p*-value
Mean (±SD)				
Sex	M = 63, F = 37	M = 35, F = 15	M = 28, F = 22	
Age (years)	69.41 (±11.73)	70.48 (±11.06)	68.34 (±12.38)	0.364
Height (cm)	162.31 (±8.54)	163.45 (±7.81)	161.17 (±9.14)	0.329
Weight (kg)	65.81 (±13.52)	66.77 (±11.92)	64.85 (±15.02)	0.414
BMI (kg/m^2^)	24.84 (±4.01)	24.97 (±4.15)	24.71 (±3.91)	0.978
Hemoglobin (g/dL)	13.82 (±2.02)	13.78 (±2.17)	13.87 (±1.87)	0.832
Hematocrit (%)	41.14 (±5.09)	41.37 (±5.35)	40.90 (±4.87)	0.644
Hemoglobin A1c (%)	6.42 (±1.53)	6.28 (±1.49)	6.55 (±1.57)	0.404
Initial Glucose (mg/dL)	161.34 (±77.56)	159.50 (±84.02)	163.18 (±71.33)	0.486
Fasting Blood sugar (mg/dL)	154.80 (±82.00)	153.18 (±90.87)	156.42 (±72.95)	0.245
PT (second)	0.98 (±0.09)	0.96 (±0.07)	0.99 (±0.10)	0.328
D-dimer (ng/mL)	347.04 (±530.47)	379.02 (±546.21)	315.06 (±517.77)	0.184
BUN (mg/dL)	13.81 (±6.64)	13.43 (±4.53)	14.19 (±8.26)	0.828
Creatinine (mg/dL)	0.95 (±0.74)	0.90 (±0.26)	1.00 (±1.01)	0.619
SBP (mmHg)	153.83 (±30.99)	146.50 (±28.72)	161.16 (±31.72)	0.171
DBP (mmHg)	87.06 (±15.80)	82.64 (±12.46)	91.48 (±17.59)	0.007^**^
WBC ( × 10^3^/μL)	8.17 (±2.68)	8.62 (±2.75)	7.73 (±2.55)	0.148
hsCRP (mg/L)	7.92 (±21.83)	11.12 (±30.43)	4.72 (±3.97)	0.041^*^
LDL (mg/dL)	104.41 (±39.11)	99.98 (±38.15)	109.02 (±39.97)	0.648
HDL (mg/dL)	46.32 (±12.16)	47.20 (±12.39)	45.40 (±11.98)	0.391
Triglycerides (mg/dL)	166.22 (±120.55)	161.74 (±129.70)	170.90 (±111.40)	0.337
Total Cholesterol (mg/dL)	170.48 (±42.59)	167.26 (±46.13)	173.70 (±38.94)	0.233
Platelet Count ( × 10^3^/μL)	234.83 (±73.96)	234.82 (±80.45)	234.84 (±67.67)	0.4
Initial NIHSS (0–42)	2.90 (±2.62)	3.12 (±3.20)	2.68 (±1.86)	0.81
Discharge NIHSS (0–42)	2.8 (±2.76)	2.72 (±3.17)	2.88 (±2.30)	0.314
Pre-MRS (0–6)	0.60 (±1.06)	0.62 (±1.05)	0.58 (±1.09)	0.704
Discharge MRS (0–6)	1.74 (±1.24)	1.66 (±1.17)	1.82 (±1.30)	0.601
Fazekas Scale (0–3)	1.96 (±0.57)	1.98 (±0.51)	1.94 (±0.63)	0.7
Medication use	*n* (%)	
Statin	89 (89.0)	42 (84.0)	47 (94.0)	0.2
Antiplatelet	98 (98.0)	49 (98.0)	49 (98.0)	1
Anticoagulation	4 (4.0)	2 (4.0)	2 (4.0)	1
PPI	85 (85.0)	42 (84.0)	43 (86.0)	1
Metformin	26 (26.0)	14 (28.0)	12 (24.0)	0.82
Antihypertensive	38 (38.0)	20 (40.0)	18 (36.0)	0.837
Laxative	3 (3.0)	2 (4.0)	1 (2.0)	1
Enteral nutrition	3 (3.0)	2 (4.0)	1 (2.0)	1

Baseline demographic, metabolic, hematologic, inflammatory, and stroke severity parameters are presented as mean ± standard deviation. Comparisons between the LAA (n = 50) and SVO (n = 50) groups were performed using appropriate statistical tests. p-values indicate between-group differences. Between-group comparisons were performed using Student's t-test or the Mann–Whitney U test for continuous variables, as appropriate. Significant differences were observed in diastolic blood pressure (DBP) and high-sensitivity C-reactive protein (hsCRP), whereas other clinical variables did not differ significantly between the subtypes. Medication use is presented as n (%). Comparisons between the LAA and SVO groups were performed using the Fisher's exact test. BMI, body mass index; PT, prothrombin time; BUN, blood urea nitrogen; WBC, white blood cells; LDL, low-density lipoprotein; HDL, high-density lipoprotein; NIHSS, National Institutes of Health Stroke Scale; mRS, modified Rankin Scale; PPI, proton-pump inhibitor; IS, Ischemic Stroke. ^*^p < 0.05, ^**^p < 0.01.

### 3.2 Shared alterations in gut microbial diversity and composition across IS etiologies

We first evaluated gut microbial richness and evenness in HCs and patients with IS. The alpha diversity indices Chao1, Shannon index, Faith's phylogenetic diversity (Faith PD), and Observed Features were significantly lower in patients with IS than in HCs (all *p* < 0.0001), indicating a consistent reduction in microbial diversity associated with IS (Figure 1B and Table 2). In the three-group comparison of HCs, LAA, and SVO, both LAA and SVO showed similarly reduced alpha diversity relative to HCs (*p* < 0.0001), with no significant difference between the subtypes (*p* > 0.05) (Figure 1C and Table 2). These results suggest that loss of microbial diversity is a shared characteristic across IS etiologies. Beta diversity analysis based on Bray–Curtis dissimilarity further demonstrated a significant shift in global community structure between the HC and IS groups (*R*^2^ = 0.0616, *p* < 0.001; Figure 1D and Table 3). Pairwise comparisons confirmed robust compositional differences between HCs and both the LAA (*R*^2^ = 0.075, *p* < 0.001) and SVO groups (*R*^2^ = 0.071, *p* < 0.001). Notably, the microbial community structures in LAA and SVO largely overlapped (*R*^2^ = 0.009, *p* = 0.595), indicating a convergent microbiome signature across IS subtypes (Figure 1E and Table 3). Overall, beta diversity analysis showed distinct separation in microbial community structure between patients with IS and HCs, while demonstrating convergence between the LAA and SVO subtypes.

**Table 2:** Comparison of alpha diversity indices among HC, IS, and stroke subtypes.

Index	Group 1	Group 2	*p*-value	Adjusted *p*-value	Significance
Chao1	Control	IS	4.75E-10	4.70E-10	^****^
Faith_PD	Control	IS	6.18E-07	6.20E-07	^****^
Observed_Feature	Control	IS	3.23E-10	3.20E-10	^****^
Pielou_Evenness	Control	IS	4.77E-06	4.80E-06	^****^
Shannon	Control	IS	3.69E-11	3.70E-11	^****^
Simpson_E	Control	IS	0.03688319	0.037	^*^
Simpson	Control	IS	1.22E-09	1.20E-09	^****^
Chao1	Control	LAA	2.30E-08	6.80E-08	^****^
Chao1	Control	SVO	2.30E-07	4.60E-07	^****^
Chao1	LAA	SVO	0.546364687	0.55	ns
Faith_PD	Control	LAA	1.90E-05	4.10E-05	^****^
Faith_PD	Control	SVO	1.40E-05	4.10E-05	^****^
Faith_PD	LAA	SVO	0.947782842	0.95	ns
Observed_Features	Control	LAA	1.80E-08	5.30E-08	^****^
Observed_Features	Control	SVO	1.60E-07	3.30E-07	^****^
Observed_Features	LAA	SVO	0.562504126	0.56	ns
Pielou_Evenness	Control	LAA	5.30E-04	0.0011	^***^
Pielou_Evenness	Control	SVO	8.90E-06	2.70E-05	^****^
Pielou_Evenness	LAA	SVO	0.501489839	0.5	ns
Shannon	Control	LAA	4.20E-08	8.30E-08	^****^
Shannon	Control	SVO	2.60E-09	7.90E-09	^****^
Shannon	LAA	SVO	0.860457035	0.86	ns
Simpson	Control	LAA	1.20E-06	2.50E-06	^****^
Simpson	Control	SVO	1.50E-08	4.60E-08	^****^
Simpson	LAA	SVO	0.666567448	0.67	ns
Simpson_E	Control	LAA	2.20E-01	0.45	ns
Simpson_E	Control	SVO	1.70E-02	0.051	^*^
Simpson_E	LAA	SVO	0.329324443	0.45	ns

Alpha diversity metrics including Chao1, Faith_PD, Observed Features, Pielou evenness, Shannon index, Simpson index, and Simpson's evenness were compared between the groups. Statistical comparisons were performed using the Wilcoxon rank-sum tests. Adjusted p-values were calculated using the Benjamini–Hochberg method. The significance levels are indicated as follows: ^*^p < 0.05, ^**^p < 0.01, ^***^p < 0.001, ^****^p < 0.0001; ns, not significant. The healthy control (HC) vs. ischemic stroke (IS) comparisons demonstrate reduced microbial richness and diversity in the IS, ischemic stroke IS group, whereas no significant differences were observed between large-artery atherosclerosis (LAA) and small-vessel occlusion (SVO) across most indices.

**Table 3:** Beta diversity comparisons based on Bray–Curtis dissimilarity among study groups.

Group 1	Group 2	*p*-value	Adjusted *p*-value	Significance	*R* ^2^	*F*
Control	IS	0.001	0.001	^***^	0.06164	9.7226
Control	SVO	0.001	0.001	^***^	0.07133	7.5276
Control	LAA	0.001	0.001	^***^	0.07546	7.9984
SVO	LAA	0.595	0.595	ns	0.00944	0.934

PERMANOVA was performed to assess the differences in the overall microbial community structure between the groups. P-values were based on 999 permutations (minimum reportable value: p = 0.001) and adjusted using the Benjamini–Hochberg method. Significant differences in beta diversity were observed between HC and IS, as well as between HC and each IS subtype (LAA and SVO). No significant differences were observed between the LAA and SVO groups. ^*^p < 0.05, ^**^p < 0.01, ^***^p < 0.001. HC, healthy control; IS, ischemic stroke; LAA, large-artery atherosclerosis; SVO, small-vessel occlusion.

### 3.3 Differential genus-level gut microbiota among the IS subtypes and HC

**Figure 2:**
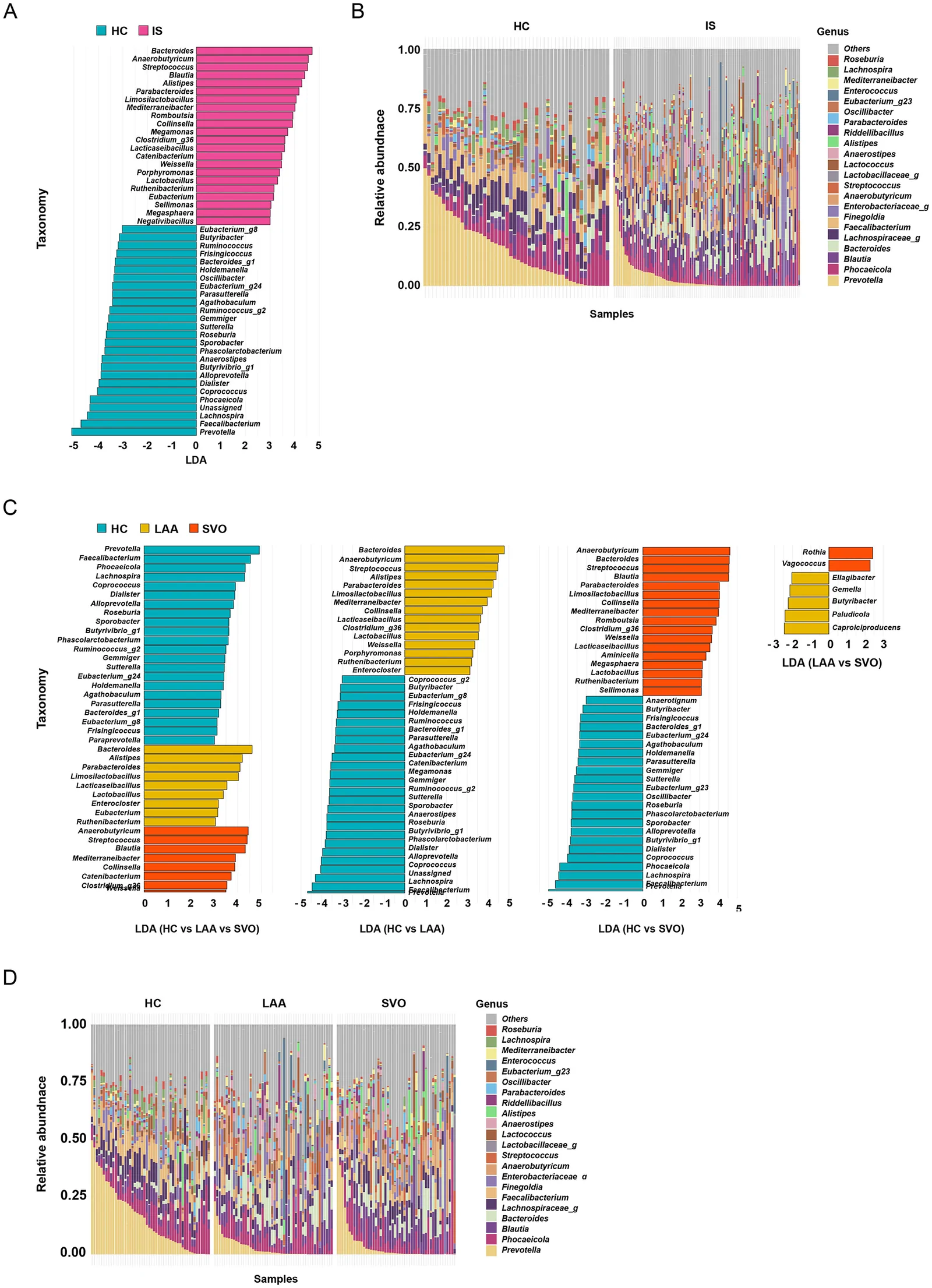
Taxonomic differences and subtype-specific microbial signatures in IS. **(A)** LEfSe analysis showing taxa enriched in HC (blue) and IS (pink; LDA > 2.0). **(B)** Genus-level relative abundance in HC and IS; each bar represents one sample. **(C)** LEfSe results among HC (blue), LAA (yellow), and SVO (orange). Comparisons with HC (HC vs. LAA, HC vs. SVO, HC vs. LAA vs. SVO) used LDA > 3.0, while LAA vs. SVO was analyzed with LDA > 2.0. **(D)** Genus-level relative abundance across HC, LAA, and SVO. (LAA, *n* = 50; SVO, *n* = 50; HC, *n* = 50). LEfSe, linear discriminant analysis effect size; LDA, linear discriminant analysis; IS, ischemic stroke; HC, healthy control; LAA, large-artery atherosclerosis; SVO, small-vessel occlusion.

To identify microbial taxa that could help differentiate patients with different subtypes of IS and HCs, we performed LEfSe analysis at the genus level. LDA showed that several taxa were significantly enriched in patients with IS, whereas others were predominantly associated with HCs (LDA score > 2.0). Notably, taxa such as *Alistipes, Anaerobutyricum, Bacteroides, Blautia*, and *Streptococcus* showed higher LDA scores in IS. In contrast, taxa including *Faecalibacterium, Lachnospira, Phocaeicola*, and *Prevotella* were more abundant in the HC group (Figure 2A and Supplementary Figure 3). Compositional bar plots further illustrated alterations in microbial community structure between the IS and HC groups. Overall, patients with IS showed greater inter-individual heterogeneity in taxonomic composition than HCs (Figure 2B and Supplementary Figure 5). To further delineate subtype-specific microbial signatures, we compared taxa-level compositions among HCs, LAA, and SVO using LDA > 3.0 for comparisons involving HCs (HC vs. LAA vs. SVO, HC vs. LAA, and HC vs. SVO) and LDA > 2.0 for the LAA vs. SVO comparison. HCs were characterized by enrichment of taxa including *Faecalibacterium, Lachnospira, Phocaeicola*, and *Prevotella*. In contrast, LAA showed higher LDA scores for taxa such as *Anaerobutyricum, Bacteroides, Parabacteroides*, and *Streptococcus*, indicating a strong discriminatory contribution. SVO showed a different microbial pattern, with enrichment of taxa including *Blautia, Romboutsia, Rothia, Sellimonas*, and *Vagococcus*, suggesting subtype-specific microbial shifts distinct from those observed in LAA. Although some taxa overlapped between LAA and SVO, the magnitude and direction of enrichment differed, supporting divergent microbial signatures between the IS subtypes (Figure 2C and Supplementary Figure 4). Compositional bar plots further highlighted subtype-level structural differences. Although a shared core microbiota was present across all groups, internal distribution patterns differed between the LAA and SVO groups. LAA showed dominant patterns centered on shifts in major genera, with a subset of individuals exhibiting clear *Bacteroides*-centered configurations. In contrast, SVO did not show a single prevailing dominant pattern, but instead displayed a more dispersed redistribution of multiple genera across individuals. These subtype-specific alterations may reflect divergent pathophysiological mechanisms underlying LAA and SVO (Figure 2D and Supplementary Figure 5).

### 3.4 Intrinsic ecological clustering of the gut microbiome independent of the Is subtype

**Figure 3:**
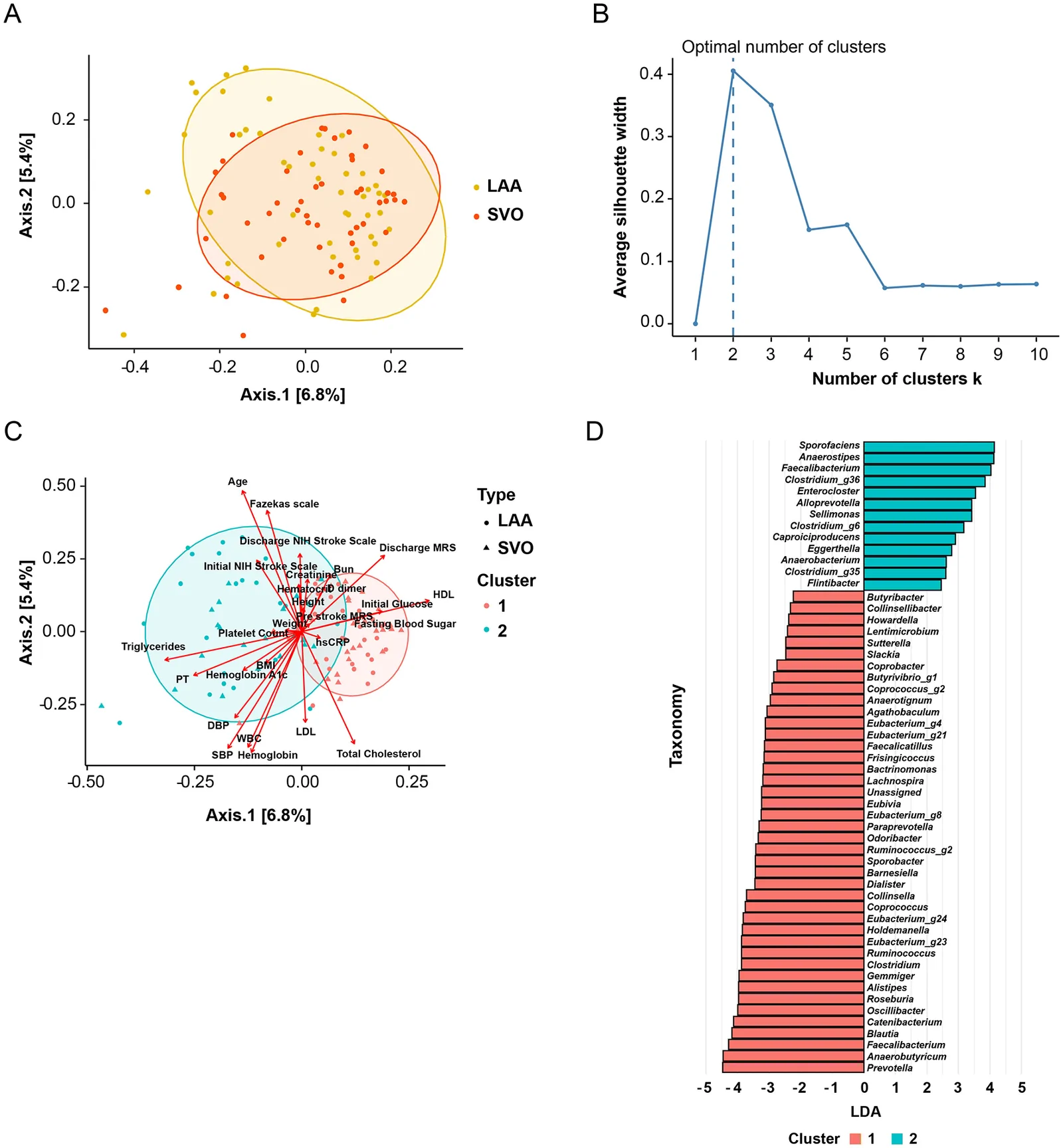
Associations between gut microbiota, clinical parameters, and cluster-specific features. **(A)** Beta diversity (Bray–Curtis) showed no significant difference between LAA (*n* = 50) and SVO (*n* = 50) (PERMANOVA *R*^2^ = 0.009, *p* = 0.595). **(B)** Silhouette analysis identified two optimal clusters. **(C)** Distance-based redundancy analysis (dbRDA) plot showing separation between Cluster 1 (red) and Cluster 2 (blue) with *envfit* vectors indicating the direction and strength of clinical associations. **(D)** LEfSe (LDA > 2.0) identifying differentially enriched taxa in each cluster. LEfSe, linear discriminant analysis effect size; LDA, linear discriminant analysis; LAA, large-artery atherosclerosis; SVO, small-vessel occlusion.

Beta diversity analysis based on Bray–Curtis dissimilarity showed no significant difference in overall microbial community composition between LAA and SVO (*R*^2^ = 0.009, *p* = 0.595; Figure 3A), indicating that conventional IS subtypes were not associated with global microbiome structure. To determine whether gut microbiome profiles could stratify patients independently of IS subtype, unsupervised clustering was performed at the genus level. Silhouette analysis identified two optimal clusters (*k* = 2; Figure 3B), classifying patients into Cluster 1 (*n* = 57; LAA = 27, SVO = 30) and Cluster 2 (*n* = 43; LAA = 23, SVO = 20). The distribution of LAA and SVO was comparable between the clusters, further supporting subtype independence. To determine whether host clinical characteristics contributed to the observed microbiome stratification, we performed dbRDA with permutation-based *envfit* testing using genus-level abundance profiles. None of the examined clinical parameters, including IS subtype, vascular risk factors, metabolic parameters, or stroke severity indices, were significantly associated with ordination structure or cluster separation (all *p* > 0.05) (Figure 3C and Table 4). These results indicate that the identified ecological subgroups were statistically independent of the measured host clinical factors. LEfSe analysis (LDA > 2.0) revealed distinct taxonomic signatures between the clusters (Figure 3D). Cluster 1 was enriched in *Anaerobutyricum, Blautia, Catenibacterium, Faecalibacterium*, and *Prevotella*, whereas Cluster 2 was enriched in *Anaerostipes, Clostridium_g36, Enterocloster, Faecalibacterium*, and *Sporofaciens*. *Faecalibacterium* was detected in both clusters but showed significantly different distribution patterns, indicating strain- or abundance-level heterogeneity within this genus. In summary, genus-level gut microbiome composition does not discriminate between conventional IS subtypes, demonstrating substantial compositional overlap between LAA and SVO. However, patients segregated into distinct ecological clusters characterized by specific taxonomic signatures that were independent of the measured clinical parameters. These findings suggest that, in IS, the gut microbiome is organized according to intrinsic ecological configurations rather than traditional clinical classification.

**Table 4:** Association of clinical parameters with gut microbiota composition based on dbRDA with *envfit* analysis.

Feature	Axis.1	Axis.2	*R* ^2^	*p*-value	Significance
Age	−0.99913	−0.04172	0.0219	0.4	ns
WBC	0.19699	0.9804	0.0671	0.06	ns
Total Cholesterol	0.9997	−0.02456	0.0401	0.09	ns
BUN	−0.92354	−0.38349	0.025	0.27	ns
Creatinine	−0.93577	−0.35262	0.0454	0.08	ns
Hemoglobin	0.88923	0.45746	0.0146	0.45	ns
Triglycerides	−0.68687	−0.72678	0	1	ns
Hematocrit	0.23326	0.97241	0.017	0.34	ns
HDL	0.44321	0.89642	0.0415	0.14	ns
Fasting Blood Sugar	−0.1798	0.9837	0.0253	0.37	ns
Platelet Count	0.21572	0.97646	0.004	0.83	ns
LDL	0.85234	−0.52299	0.0536	0.06	ns
Hemoglobin A1c	0.96666	−0.25606	0.0016	0.97	ns
PT	0.13628	−0.99067	0.0029	0.82	ns
hsCRP	−0.31758	0.94823	0.0624	0.07	ns
Initial Glucose	−0.48546	0.87426	0.0313	0.25	ns
D-Dimer	−0.85902	−0.51193	0.0375	0.12	ns
SBP	0.57936	0.81507	0.0011	0.95	ns
DBP	0.8983	−0.43938	0.0006	0.95	ns
Height	0.99239	0.1231	0.0017	0.95	ns
Weight	−0.5004	0.86579	0.0106	0.66	ns
BMI	−0.77494	0.63204	0.0106	0.56	ns
Initial NIH Stroke Scale	0.42687	0.90431	0.0021	0.9	ns
Discharge NIH Stroke Scale	−0.38741	0.9008	0.0008	0.98	ns
Pre-stroke MRS	−0.18252	0.9832	0.0011	0.96	ns
Discharge MRS	−0.67172	0.7408	0.0279	0.39	ns
Fazekas Scale	−0.99384	−0.11082	0.0388	0.15	ns

Distance-based redundancy analysis (dbRDA) was performed using genus-level abundance profiles to evaluate the associations between the microbial community structure and clinical parameters. Envfit permutation testing was conducted to assess the significance of each parameter. Axes 1 and 2 represent the correlation coefficients of ordination axes. R^2^ indicates the proportion of variance explained by each parameter. None of the examined clinical parameters showed significant associations with the microbial community structure (all p > 0.05). IS, ischemic stroke; WBC, white blood cells; BUN, blood urea nitrogen; HDL, high-density lipoprotein; LDL, low-density lipoprotein; PT, prothrombin time; hsCRP, high-sensitivity C-reactive protein; SBP, systolic blood pressure; DBP, diastolic blood pressure; BMI, body mass index; NIHSS, National Institutes of Health Stroke Scale; mRS, modified Rankin Scale.

### 3.5 Subtype-specific correlations between gut microbial taxa and clinical parameters in Is

**Figure 4:**
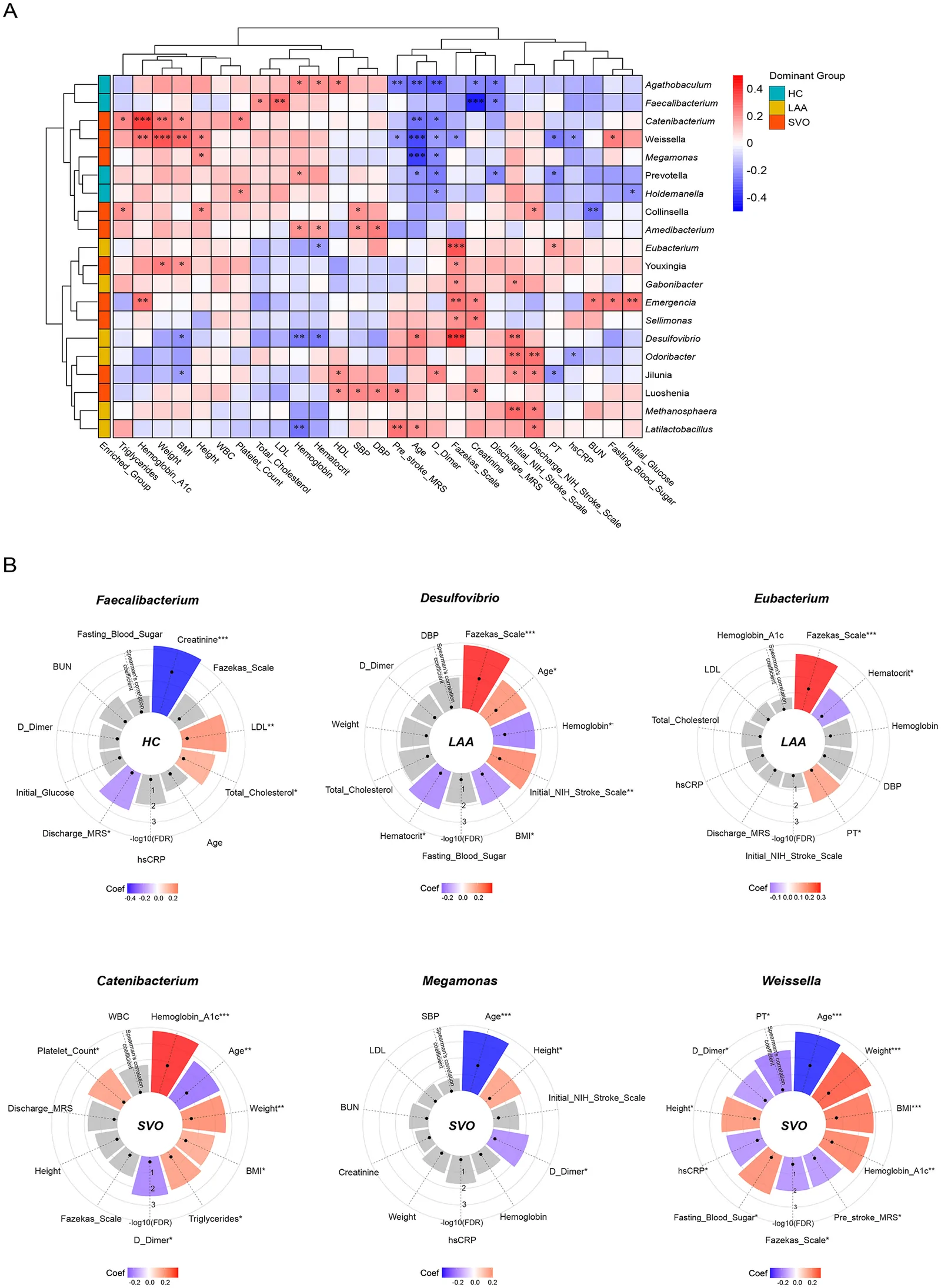
Correlation heatmap of gut microbial taxa and clinical parameters in IS patients. **(A)** Spearman correlation analysis was performed between genus-level relative abundances and clinical parameters within the IS cohort. HC-, LAA-, or SVO-dominant according to the three-group LEfSe results. Heatmap colors represent correlation direction and strength (red, positive; blue, negative) *p < 0.05, **p < 0.01, ***p < 0.001. **(B)** FDR-significant correlations (*q* < 0.05) are visualized as polar bar plots. The radial coordinate (bar length) represents –log10 (*q*), with black markers indicating the corresponding –log10 (*q*) values for each clinical parameter. Color encodes the Spearman correlation coefficient (ρ; red, positive; blue, negative). The LAA and SVO groups each comprised 50 participants. IS, ischemic stroke; LEfSe, linear discriminant analysis effect size; FDR, false discovery rate; IS, ischemic stroke; HC, healthy control; LAA, large-artery atherosclerosis; SVO, small-vessel occlusion.

To assess the clinical relevance of gut microbiome alterations in IS, Spearman correlation analysis was performed between genus-level relative abundances and clinical parameters within the IS cohort, including patients with LAA and SVO. Based on the three-group LEfSe analysis, taxa were classified as HC-, LAA-, or SVO-dominant (Supplementary Figure 6 and Supplementary Tables 4, 5). HC-dominant taxa showed negative correlations with age, D-dimer levels, and mRS scores before stroke and at discharge. LAA-dominant taxa were positively correlated with Fazekas scale scores and initial and discharge NIHSS scores. SVO-dominant taxa were positively correlated with HbA1c, body weight, BMI, and initial and discharge NIHSS scores (Figure 4A and Table 5). After FDR adjustment (*q* < 0.05), significant associations were observed between *Faecalibacterium* and measured creatinine levels among HC-dominant taxa. Significant associations were also observed between *Desulfovibrio* and *Eubacterium* and Fazekas scale scores among LAA-dominant taxa. Similarly, *Catenibacterium* spp. were significantly associated with HbA1c levels. *Megamonas* and *Weissella* were also significantly associated with age among SVO-dominant taxa. To define subtype-associated taxa with stronger statistical support, taxa identified by three-group LefSe analysis (LDA > 3.0) and/or taxa with FDR-significant Spearman correlations (*q* < 0.05) were selected (Figure 4B). This approach yielded 10 taxa: 3 HC-dominant taxa (*Agathobaculum, Holdemanella*, and *Prevotella*), 2 LAA-dominant taxa (*Desulfovibrio* and *Eubacterium*), and 5 SVO-dominant taxa (*Catenibacterium, Collinsella, Megamonas, Sellimonas*, and *Weissella*). Among the selected taxa, HC-dominant genus *Agathobaculum* was negatively correlated with age, D-dimer levels, and discharge mRS scores and positively correlated with hemoglobin, hematocrit, and HDL cholesterol levels. *Prevotella* was negatively correlated with D-dimer, age, and discharge mRS scores, and *Holdemanella* was negatively correlated with D-dimer levels. The LAA-dominant genera *Desulfovibrio* and *Eubacterium* were positively correlated with Fazekas scale scores, and *Desulfovibrio* was also correlated with initial NIHSS scores. Among the SVO-dominant taxa, *Catenibacterium* was positively correlated with HbA1c. *Weissella* was positively correlated with HbA1c, fasting blood sugar, body weight, and BMI, and negatively correlated with age and D-dimer levels. *Megamonas* was negatively correlated with age, *Collinsella* was positively correlated with discharge NIHSS scores, and *Sellimonas* was positively correlated with the Fazekas scale scores (Table 6 and Supplementary Table 6). Overall, these subtype-specific microbial associations with clinical severity and metabolic parameters suggest that gut microbial profiles differ in their relationships with disease burden and metabolic status between LAA and SVO. However, all associations are cross-sectional and hypothesis-generating. Causal interpretation requires prospective and experimental validation.

**Table 5:** Correlation between subtype-enriched taxa and clinical parameters in IS cohort.

Control	Variable	Coef	Significance (*p*)	*p*-value	Significance (*q*)	*q*-value (FDR)
*Agathobaculum*	D_Dimer	−0.304	^**^	0.002		0.175
	Age	−0.291	^**^	0.003		0.087
	Pre_stroke_MRS	−0.258	^**^	0.010		0.216
	Discharge_MRS	−0.241	^*^	0.016		0.625
	Hemoglobin	0.228	^*^	0.022		0.248
	Creatinine	−0.225	^*^	0.024		0.346
	HDL	0.221	^*^	0.027		0.537
	Hematocrit	0.201	^*^	0.045		0.433
*Faecalibacterium*	Creatinine	−0.423	^***^	0.000	^*^	0.002
	LDL	0.283	^**^	0.004		0.454
	Discharge_MRS	−0.246	^*^	0.014		0.625
	Total_Cholesterol	0.217	^*^	0.030		0.511
*Holdemanella*	Platelet_Count	0.206	^*^	0.040		0.792
	D_Dimer	−0.204	^*^	0.042		0.643
	Initial_Glucose	−0.200	^*^	0.046		0.440
*Prevotella*	D_Dimer	−0.241	^*^	0.016		0.491
	Discharge_MRS	−0.234	^*^	0.019		0.625
	PT	−0.223	^*^	0.026		0.760
	Age	−0.211	^*^	0.035		0.226
	Hemoglobin	0.198	^*^	0.048		0.353
LAA						
*Desulfovibrio*	Fazekas_Scale	0.387	^***^	0.000	^*^	0.011
	Initial_NIH_Stroke_Scale	0.272	^**^	0.006		0.236
	Hemoglobin	−0.259	^**^	0.009		0.183
	Age	0.254	^*^	0.011		0.140
	Hematocrit	−0.242	^*^	0.015		0.433
	BMI	−0.217	^*^	0.030		0.375
*Eubacterium*	Fazekas_Scale	0.340	^***^	0.001	^*^	0.043
	PT	0.199	^*^	0.047		0.760
	Hematocrit	−0.198	^*^	0.049		0.433
*Gabonibacter*	Initial_NIH_Stroke_Scale	0.215	^*^	0.031		0.317
	Fazekas_Scale	0.203	^*^	0.043		0.387
*Latilactobacillus*	Hemoglobin	−0.273	^**^	0.006		0.183
	Pre_stroke_MRS	0.269	^**^	0.007		0.215
	Discharge_NIH_Stroke_Scale	0.255	^*^	0.010		0.370
	Age	0.205	^*^	0.040		0.226
*Methanosphaera*	Initial_NIH_Stroke_Scale	0.285	^**^	0.004		0.236
	Discharge_NIH_Stroke_Scale	0.248	^*^	0.013		0.370
*Odoribacter*	Discharge_NIH_Stroke_Scale	0.270	^**^	0.007		0.370
	Initial_NIH_Stroke_Scale	0.268	^**^	0.007		0.236
	hsCRP	−0.197	^*^	0.049		0.482
SVO						
*Amedibacterium*	DBP	0.242	^*^	0.015		0.645
	SBP	0.211	^*^	0.035		0.720
	Hemoglobin	0.208	^*^	0.037		0.309
	Hematocrit	0.199	^*^	0.047		0.433
*Catenibacterium*	Hemoglobin_A1c	0.378	^***^	0.000	^*^	0.016
	Age	−0.276	^**^	0.005		0.107
	Weight	0.264	^**^	0.008		0.279
	D_Dimer	−0.244	^*^	0.015		0.491
	Triglycerides	0.226	^*^	0.024		0.418
	Platelet_Count	0.222	^*^	0.027		0.792
	BMI	0.202	^*^	0.043		0.394
*Collinsella*	BUN	−0.261	^**^	0.009		0.548
	SBP	0.216	^*^	0.031		0.720
	Discharge_NIH_Stroke_Scale	0.205	^*^	0.041		0.392
	Height	0.203	^*^	0.043		0.514
	Triglycerides	0.201	^*^	0.045		0.506
*Emergencia*	Fazekas_Scale	0.274	^**^	0.006		0.246
	Hemoglobin_A1c	0.264	^**^	0.008		0.253
	Initial_Glucose	0.263	^**^	0.008		0.392
	BUN	0.256	^*^	0.010		0.548
	Fasting_Blood_Sugar	0.246	^*^	0.013		0.414
	Creatinine	0.232	^*^	0.020		0.346
*Jilunia*	Discharge_NIH_Stroke_Scale	0.228	^*^	0.022		0.370
	D_Dimer	0.228	^*^	0.023		0.518
	Initial_NIH_Stroke_Scale	0.215	^*^	0.032		0.317
	PT	−0.202	^*^	0.044		0.760
	HDL	0.200	^*^	0.046		0.537
	BMI	−0.198	^*^	0.048		0.394
*Luoshenia*	SBP	0.235	^*^	0.018		0.720
	Pre_stroke_MRS	0.226	^*^	0.024		0.290
	DBP	0.221	^*^	0.027		0.645
	Creatinine	0.207	^*^	0.038		0.383
	HDL	0.207	^*^	0.039		0.537
*Megamonas*	Age	−0.368	^***^	0.000	^*^	0.021
	D_Dimer	−0.222	^*^	0.026		0.518
	Height	0.212	^*^	0.034		0.466
*Sellimonas*	Creatinine	0.250	^*^	0.012		0.303
	Fazekas_Scale	0.215	^*^	0.032		0.387
*Weissella*	Age	−0.357	^***^	0.000	^*^	0.021
	Weight	0.327	^***^	0.001		0.141
	BMI	0.287	^**^	0.004		0.220
	Hemoglobin_A1c	0.268	^**^	0.007		0.253
	PT	−0.247	^*^	0.013		0.692
	Fasting_Blood_Sugar	0.246	^*^	0.014		0.414
	Height	0.230	^*^	0.021		0.466
	hsCRP	−0.206	^*^	0.040		0.446
	Fazekas_Scale	−0.204	^*^	0.042		0.387
	Pre_stroke_MRS	−0.199	^*^	0.047		0.370
	D_Dimer	−0.198	^*^	0.048		0.643
*Youxingia*	Weight	0.252	^*^	0.011		0.279
	BMI	0.220	^*^	0.028		0.375
	Fazekas_Scale	0.214	^*^	0.032		0.387

Spearman's correlation analysis was performed between genus-level relative abundances and clinical parameters in the IS cohort. Correlation coefficients (Coef) are shown. p-values were adjusted for multiple comparisons using the Benjamini-Hochberg FDR method. Significance levels are indicated as follows: ^*^ p < 0.05, ^**^ p < 0.01, ^***^ p < 0.001, ^*^ q < 0.05, ^**^ q < 0.01, ^***^ q < 0.001. IS, ischemic stroke; BMI, body mass index; PT, prothrombin time; BUN, blood urea nitrogen; WBC, white blood cells; LDL, low-density lipoprotein; HDL, high-density lipoprotein; NIHSS, National Institutes of Health Stroke Scale; mRS, modified Rankin Scale; hsCRP, high-sensitivity C-reactive protein; SBP, systolic blood pressure; DBP, diastolic blood pressure.

**Table 6:** Subtype-enriched gut microbial taxa and their potential relevance to IS pathophysiology.

Control	Positive correlations	Negative correlations	Known/Proposed mechanism	Disease/Pathway	Key findings
*Agathobaculum*	HDL^*^, Hemoglobin^*^, Hematocrit^*^	Creatinine^*^, D-Dimer^**^, Discharge MRS^*^, Age^**^, Pre stroke MRS^**^	SCFA (butyrate) production	Gut microbial metabolism	“*A. butyriciproducens* gen. nov. sp. nov., a strict anaerobic, butyrate-producing gut bacterium isolated from human feces (Ahn et al., 2016).”
			Neuroprotection *via* oxidative stress regulation	Neuroinflammation/ Neurodegeneration	“*A. butyriciproducens* mitigated neurotoxicity by regulating the AKT/Nrf2/ARE signaling pathway and astrocyte activation (Lee et al., 2022).”
*Holdemanella*	Platelet Count^*^	D-Dimer^*^, Initial Glucose^*^	Immune modulation (pro-inflammatory cytokine shift)	Systemic inflammation	“*H. porci* elevated the frequency of both CCR5+ CD4+ T cells and the ratio of TNF-α/IL-10 (Yamada et al., 2021).”
			Enhance GLP-1 neural signaling and sensitivity	Glucose regulation	“This study shows that *H. biformis*, isolated from the feces of a metabolically healthy volunteer, ameliorates hyperglycemia, improves oral glucose tolerance and restores gluconeogenesis and insulin signaling in the liver of obese mice (Romani-Perez et al., 2021).”
*Prevotella*	Hemoglobin^*^	D-Dimer^*^, Discharge MRS^*^, Age^*^, PT^*^	BCAA biosynthesis	Insulin resistance/Metabolic regulation	“*P*. *copri* was identified as the main species driving the association between biosynthesis of branched-chain amino acids and insulin resistance (Pedersen et al., 2016).”
			Thrombo-inflammatory association	Hypercoagulable state in ischemic stroke	“Patients in a hypercoagulable state exhibited significantly decreased *P*. abundance compared with patients with normal coagulation function (Li et al., 2025).”
LAA					
*Desulfovibrio*	Fazekas Scale^***^, Initial NIH Stroke Scale ^**^, Age^*^	Hemoglobin^**^, Hematocrit^**^, BMI^*^	Opportunistic pathobiontenrichment in LAA stroke/TIA dysbiosis	LAA stroke/TIA	“Stroke and transient ischemic attack patients had more opportunistic pathogens, such as *Enterobacter, Megasphaera, Oscillibacter*, and *D* (Yin et al., 2015).”
			Increased gut permeability	Endothelial inflammation(TLR4/NF-κB)/atherosclerosis	“Our results demonstrate that *D. desulfuricans* can enhance the development of atherosclerosis by increasing intestinal permeability and host inflammatory response (Zhang et al., 2023).”
*Eubacterium*	PT^*^, Fazekas Scale^***^	Hematocrit^*^	Poor prognosis–associated genus	Functional outcomeafter ischemic stroke	“Strikingly, genus Ruminococcaceae *UCG005* and the genus *E*. *oxidoreducens* group showed significantly negative effects on stroke prognosis (Zhu et al., 2024).”
SVO					
*Catenibacterium*	Hemoglibin A1c^***^, Weight^**^, Triglycerides^*^, Platelet Count^*^, BMI^*^	Age^**^, D-Dimer^*^	Gut ecosystem disruption	Dysbiosis-associated LPS burden	“Instead, our findings suggest that *C*. acts as a disruptor of the gut ecosystem, fostering a dysbiotic microbial community that contributes to elevated LPS production (Wei et al., 2025).”
*Collinsella*	Height^*^, SBP^*^, Triglycerides^*^, Discharge NIH Stroke Scale^*^	Bun^**^	Dyslipidemia-associated genus	Lipid metabolism/Metabolic markers	“*C*. showed a positive association with serum cholesterol in an integrated microbiome–lipid profile analysis (Lahti et al., 2013).”
			Dyslipidemia-associated genus	Hypertension/ Hyperlipidemia/type 2 diabetes	“Those of the *C*. genus were also significantly increased in the HT, HL, T2D, RISK2, and RISK3 groups compared to HC (Takagi et al., 2020).”
			Increased gut permeability	Gut barrier disruption/Inflammation	“*C*. administration led to a significant increase in gut permeability compared with pre-treatment (Chen et al., 2016).”
*Megamonas*	Height^*^	Age^***^, D-Dimer^*^	Systemic inflammation–associated dysbiosis	Stroke severity	“Conversely, pathogenic bacterium including *M, Lactobacillus mucosae, Megasphaera*, and *Ruminococcaceae UCG 004* showed positive correlations with systemic inflammation, stroke severity, and poor functional outcome (He Q. et al., 2024).”
*Sellimonas*	Creatinine^*^, Fazekas Scale^*^	X	Inflammation-associated pathogen	Stroke severity/Poor functional outcome	“Additionally, other pathogenic bacterium such as *S, S. intestinalis*, displayed positive correlations specifically with stroke severity and poor functional outcome (He P. et al., 2024).”
*Weissella*	Height^*^, Hemoglobin A1c^**^, Weight^***^, BMI^**^, Fasting Blood Sugar^*^	hsCRP^*^, Fazekas Scale^*^, D-Dimer^*^, Age^***^, PT^*^, Pre stroke MRS^*^	Metabolic modulation	Obesity/hepatic steatosis	“*W. cibaria MG5285* attenuated fat accumulation in adipose tissue and hepatic steatosis in high-fat diet-induced obese mice (Choi et al., 2021).”
			Gut barrier stabilization	Intestinal epithelial integrity	“Treatment with *W. confusa F213* significantly reduced epithelial permeability and maintained ZO-1 protein expression under hydrogen peroxide-induced oxidative stress (Fatmawati et al., 2020).”

Summary of genus-level taxa identified through clinical correlation analyses, including subtype-specific positive and negative associations with clinical parameters, proposed biological mechanisms, relevant disease pathways, and supporting evidence from the literature. The table highlights putative host-microbiome interaction patterns linking enriched genera to thrombo-inflammatory, metabolic, vascular, and neurodegenerative processes in large-artery atherosclerosis (LAA) and small-vessel occlusion (SVO). IS, ischemic stroke; BMI, body mass index; PT, prothrombin time; BUN, blood urea nitrogen; WBC, white blood cells; LDL, low-density lipoprotein; HDL, high-density lipoprotein; NIHSS, National Institutes of Health Stroke Scale; mRS, modified Rankin Scale; SCFA, short-chain fatty acid; BCAA, branched-chain amino acid; LPS, lipopolysaccharide. ^*^p < 0.05, ^*^^*^p < 0.01, and ^*^^*^^*^p < 0.001.

## 4 Discussion

This study investigated gut microbiome alterations in patients with IS, with a focus on the LAA and SVO subtypes, by integrating taxonomic profiles with clinical parameters. Although both subtypes showed reduced microbial diversity and broadly similar community compositions, these global features did not distinguish LAA from SVO. However, genus-level associations with clinical parameters revealed distinct patterns linking specific bacterial taxa to subtype-relevant clinical features. These findings support the hypothesis that gut microbiome composition reflects different pathophysiological processes underlying LAA and SVO. The results also suggest that specific genus-level taxa are differentially associated with clinical parameters relevant to each IS subtype, providing a basis for hypothesis-driven research on the potential contribution of the microbiome to subtype-specific pathophysiology. Importantly, all reported associations were cross-sectional and observational and should not be interpreted as evidence of causality.

In our study, patients with IS showed significantly reduced alpha diversity and distinct beta diversity compared to HCs, whereas no significant differences in either alpha or beta diversity were detected between the LAA and SVO groups. This overall pattern is consistent with previous reports of gut microbiome dysbiosis in patients with IS (Haak et al., 2021; Xia et al., 2019; Yamashiro et al., 2017). However, a recent systematic review concluded that alpha diversity relative to HCs is heterogeneous across studies, with reports of higher, lower, or unchanged alpha diversity across cohorts. This variability may reflect differences in geographic region or ethnicity, sequencing technologies or platforms, and sample collection timing and methods (Zhang et al., 2024). The *R*^2^ values for beta diversity differences between HCs and patients with IS (HC vs. IS: *R*^2^ = 0.062; HC vs. LAA: *R*^2^ = 0.075; HC vs. SVO: *R*^2^ = 0.071) indicated that IS group membership accounted for only a small proportion of total microbiome variance. These results are consistent with broader human gut microbiome studies, in which disease-associated PERMANOVA *R*^2^ values typically fall within the 5%−15% range (Falony et al., 2016; Xia et al., 2019). This pattern reflects the substantial contribution of interindividual factors, such as diet, host genetics, and prior antibiotic exposure, to overall community composition. The high F-statistics and significant *p*-values (all *p* < 0.001) confirmed that these differences were statistically robust. However, the modest *R*^2^ values suggest that IS-associated microbiome alterations are more readily detected at the level of specific taxa than through global community restructuring. This interpretation motivates the genus-level analytical framework used in the present study. Therefore, careful reporting of study parameters is important for cross-study comparisons.

In addition to comparisons between IS and HCs, few studies have examined microbiome differences among IS subtype-related cohorts. In an LAA-related cohort that included patients with LAA stroke or TIA, alpha diversity was higher in patients than in HCs, and beta diversity separated patients from HCs (Yin et al., 2015). In an SVO-related cohort of patients with lacunar infarction, the infarction group showed lower overall richness of observed operational taxonomic units (OTUs) than HCs and exhibited a beta diversity pattern distinct from that of HCs (Xiang et al., 2020). However, direct head-to-head comparisons of LAA and SVO remain limited. Our finding of similar alpha and beta diversity between these subtypes suggests that subtype-relevant differences may be better captured at the taxa level. Accordingly, we used three-group LEfSe analysis to define HC-, LAA-, and SVO-dominant taxa.

In the three-group LEfSe analysis, the HC, LAA, and SVO groups showed distinct taxa with minimal overlap. This finding indicates that gut microbiome differences between IS subsets are driven more by enrichment of specific taxa than by overall diversity. Because taxonomic differences persisted despite similar alpha and beta diversity, we used unsupervised clustering to identify a microbiome-driven stratification among IS patients that transcended conventional LAA and SVO categories.

Unsupervised clustering revealed two ecologically distinct microbiome configurations within the IS cohort, each comprising patients with both LAA and SVO. Cluster 1 was enriched in taxa associated with commensals and SCFA-producing functions, including butyrate-producing genera such as *Faecalibacterium* and *Roseburia* (Louis and Flint, 2009; Sokol et al., 2008), whereas cluster 2 showed enrichment of taxa more frequently reported in dysbiotic and pro-inflammatory contexts, including *Eggerthella* and *Enterocloster* (Agudelo et al., 2025; Mbaye et al., 2023). Importantly, dbRDA with *envfit* showed that none of the measured clinical parameters significantly explained the observed cluster separation. Rather than weakening the ecological validity of the clusters, this null finding suggests that gut microbiome organization in IS may be governed by intrinsic ecological dynamics not captured by the measured clinical variables. Because cluster-level ordination did not reveal associations between taxa and clinical parameters, we performed genus-level spearman correlation analysis to identify specific microbial taxa associated with clinical parameters within the IS cohort.

*Agathobaculum* is an SCFA-producing genus (Ahn et al., 2016) that has been reported to have anti-inflammatory and neuroprotective effects, and mainly by suppressing neuroinflammatory responses and reducing glial activation (Liu et al., 2021). Experimental evidence further suggests that *Agathobaculum butyriciproducens* can reduce neurotoxicity by regulating oxidative stress pathways and astrocyte activation (Lee et al., 2022). These experimental findings are consistent with observations in human IS cohorts, in which the depletion of SCFA-producing taxa, including butyrate producers, has been associated with poorer functional outcomes and greater stroke severity (Li et al., 2025). Taken together, the observed depletion of *Agathobaculum* in patients with IS is consistent with the hypothesis that microbiome-derived neuroprotective capacity is reduced. However, causal inference cannot be drawn from these cross-sectional data.

*Holdemanella* has been suggested to influence host immune and metabolic regulation. A previous study using human gut lamina propria mononuclear cells showed that *Holdemanella porci* enhanced mucosal immune activation by increasing CCR5^+^ CD4^+^ T-cell frequencies and the TNF-α/IL-10 ratio, consistent with a more pro-inflammatory mucosal immune response (Yamada et al., 2021). Another study showed that *Holdemanella biformis* reduced hyperglycemia and improved glucose tolerance by enhancing GLP-1-related neural signaling and sensitivity (Romani-Perez et al., 2021). In acute IS, hyperglycemia and elevated D-dimer levels are markers of stress and thromboinflammatory activity (Johnson et al., 2019; Yao et al., 2023). These findings suggest that *Holdemanella* may be associated with immune and metabolic pathways in IS. However, whether this relationship is causal or reactive remains unclear.

Similarly, *Prevotella* has been implicated in host metabolic regulation through its branched-chain amino acid (BCAA) biosynthetic capacity. Specifically, *Prevotella copri* was identified in a large non-diabetic cohort as a major contributor to BCAA synthesis linked to insulin resistance and impaired glucose metabolism (Pedersen et al., 2016). In addition, reduced *Prevotella* abundance has been observed in acute IS cohorts among patients with hypercoagulable states compared with those with normal coagulation profiles (Li et al., 2025).

Among the LAA-dominant taxa, *Desulfovibrio* and *Eubacterium* were positively correlated with white matter injury, as reflected by Fazekas scale scores, and *Desulfovibrio* was correlated with stroke severity, as reflected by NIHSS scores. Increased abundance of *Desulfovibrio* has previously been reported in patients with LAA or TIA compared with asymptomatic controls (Yin et al., 2015). Experimental studies further demonstrated that *Desulfovibrio desulfuricans* can aggravate atherosclerotic lesion development in ApoE (-/-) mice by increasing intestinal permeability and promoting endotoxin-driven vascular inflammation through the TLR4/NF-κB pathway (Zhang et al., 2023). However, these findings require further validation in human vascular disease models. Our observation that *Desulfovibrio* correlated with Fazekas and NIHSS scores in patients with LAA is consistent with human cohort data and may reflect a link between gut barrier disruption and atherosclerotic burden, pending experimental confirmation.

In addition, *Eubacterium* was positively correlated with the Fazekas scale scores, indicating an association with greater white matter injury. Although white matter hyperintensities are classically linked to SVO, Fazekas scale scores can also increase in LAA because of chronic hypoperfusion and coexisting small-vessel pathology, often reflecting greater atherosclerotic burden and poorer outcomes (Baik et al., 2017). Consistent with this interpretation, the *Eubacterium oxidoreducens* group has previously been associated with unfavorable IS outcomes, although direct causal mechanisms remain unclear and may involve endotoxin-related host responses (Zhu et al., 2024).

SVO-dominant taxa were associated with metabolic risk factors such as hyperglycemia, obesity, and dyslipidemia. In cohorts of patients with acute IS, elevated HbA1c levels and metabolic risk markers have been associated with distinct microbial signatures, supporting the concept that glycemic dysregulation and gut microbiome composition are linked in the context of stroke (Yu et al., 2024). A recent study reported that *Catenibacterium* promotes a dysbiotic community and elevated lipopolysaccharide (LPS) production (Wei et al., 2025). Because LPS has been linked to insulin resistance and abdominal obesity in humans (Troseid et al., 2013), this provides a biologically plausible, although unconfirmed in human stroke populations, pathway connecting *Catenibacterium* enrichment to the glycemic and obesity-related features observed in our SVO cohort.

Higher abundance of *Collinsella* has previously been reported in individuals with hyperlipidemia and hypertension (Takagi et al., 2020). More specifically, a cohort study of patients with cerebral small-vessel disease (CSVD) demonstrated that *Collinsella* abundance was significantly higher in patients with CSVD and was positively correlated with white matter hyperintensity scores and plasma TNF-α levels. These findings suggest that inflammatory responses mediated by *Collinsella* may contribute to the severity of small-vessel cerebrovascular disease (Shi et al., 2023). Experimental data indicate that *Collinsella* disrupts epithelial tight junction integrity and promotes IL-17A–related inflammatory signaling, providing a plausible mechanistic basis for these clinical observations (He P. et al., 2024). Taken together, findings from human cohorts and experimental models support a pathway linking *Collinsella* enrichment to neuroinflammation and white matter injury, both of which are relevant to the pathophysiology of SVO.

Previous reports have associated *Megamonas* and *Sellimonas/Sellimonas intestinalis* with systemic inflammation, stroke severity, and poor functional outcomes (He P. et al., 2024). This pattern was consistent with the correlations identified in the SVO cohort.

In contrast, *Weissella* spp. showed a distinct pattern in our cohort. Although *Weissella* was positively correlated with hemoglobin A1c, fasting blood glucose, body weight, and BMI, it showed negative correlations with age, Fazekas scale scores, and pre-stroke mRS scores, suggesting that this genus aligns with obesity and poor glycemic control but is dissociated from clinical parameters of white matter injury and IS severity. Although there is no direct human evidence at the genus level linking *Weissella* abundance to HbA1c or fasting glucose levels, *Weissella cibaria* was enriched in the late-stage diabetes group of an elderly type 2 diabetes cohort, suggesting that its abundance may reflect cumulative metabolic burden rather than short-term glycemic control (Choi et al., 2021). Recently, *Weissella confusa* was shown to preserve intestinal epithelial barrier integrity under oxidative stress by reducing epithelial permeability and preserving the tight junction protein ZO-1 (Fatmawati et al., 2020). Despite its association with hyperglycemia and obesity, *Weissella* may exert barrier-stabilizing effects that could modify vascular injury during chronic hyperglycemia. The subtype-associated taxa identified in this study, particularly *Desulfovibrio* and *Eubacterium* in LAA, and *Catenibacterium, Weissella*, and *Collinsella* in SVO, are promising candidates for future investigation as exploratory microbiome-based biomarkers for IS subtype stratification. If these associations are replicated in larger prospective cohorts, they could provide a basis for hypothesis-driven trials of microbiome-targeted interventions in specific IS subtypes. However, clinical translation requires prospective validation, and the present findings should not be interpreted as supporting immediate therapeutic application.

This study has several limitations. First, the limited clinical data available for HCs restricted the direct comparisons of clinical microbiota associations across groups. Although identical technical protocols were applied to HC samples obtained from a separate population-based microbiome project, differences in recruitment setting and unmeasured environmental factors may have introduced residual batch effects. Second, patient stool samples were collected within 1 week of stroke onset during the acute phase of hospitalization; therefore, acute stress responses may have altered microbial profiles relative to premorbid states. There was no significant difference in medication use between the LAA and SVO groups. However, medication data was unavailable for HCs, preventing adjustment for microbiome-modifying medications in cross-group analyses. In addition, the absence of taxon-level medication adjustment precludes exclusion of within-group effects of individual medications on specific taxa. Future studies should incorporate medication use as a covariate in multivariable models. Third, we did not measure microbiota-derived metabolites, such as SCFAs, TMAO, bile acids, and PAGln, or perform functional pathway inference, such as PICRUSt2. This limits the mechanistic interpretation of the observed compositional differences. Therefore, the proposed metabolite-mediated pathways are hypothetical and require validation through targeted metabolomic profiling in future studies. In addition, owing to pipeline constraints, formal validation of LEfSe-identified taxa using compositional methods, such as ANCOM-BC2 or ALDEx2, was not performed; this remains an important direction for future replication studies. Finally, mechanistic validation was not performed in this study. Future *in vivo* and *in vitro* studies are required to determine whether the identified taxa contribute causally to the pathogenesis of LAA and SVO. Taxon-clinical associations are based on Spearman correlations without full multivariable adjustment because the sample size was small for adequately powered regression modeling. Residual confounding cannot be excluded, including by sex: male predominance was noted across all groups (LAA, SVO, and HC), although it did not differ significantly between LAA and SVO or between IS and HC groups (Fisher's exact test, both *p* > 0.05). Nonetheless, confirmatory studies with larger cohorts incorporating multivariable models are needed.

Accordingly, future studies should incorporate matched clinical datasets from HCs, comprehensive functional assessments of host–microbiome interactions, and experimental validation of candidate taxa. These approaches will strengthen causal inference and clarify the biological mechanisms underlying subtype-specific microbial patterns, including the potential contribution of metabolite-related taxa.

## 5 Conclusion

Although alpha and beta diversity did not differ significantly between LAA and SVO, genus-level analysis revealed subtype-associated taxa with distinct clinical correlates. These exploratory cross-sectional findings identify taxa that warrant validation in future hypothesis-driven studies using experimental models and prospective longitudinal cohorts, including assessment of microbiome-derived metabolites.

## Data Availability

The data presented in this study are deposited in the NCBI Sequence Read Archive (SRA) repository under BioProject accession number PRJNA1441156.
