# Supplementary material for: Subtype-associated gut microbial taxa and their clinical correlates in acute ischemic stroke: comparison of large-artery atherosclerosis and small-vessel occlusion
Source: Front Microbiol. 2026 Sep 11;17:1867057. doi: 10.3389/fmicb.2026.1867057 (PMC13613636; doi:10.3389/fmicb.2026.1867057)
Supplement: Supplementary file 1 [file Data_Sheet_1.pdf]

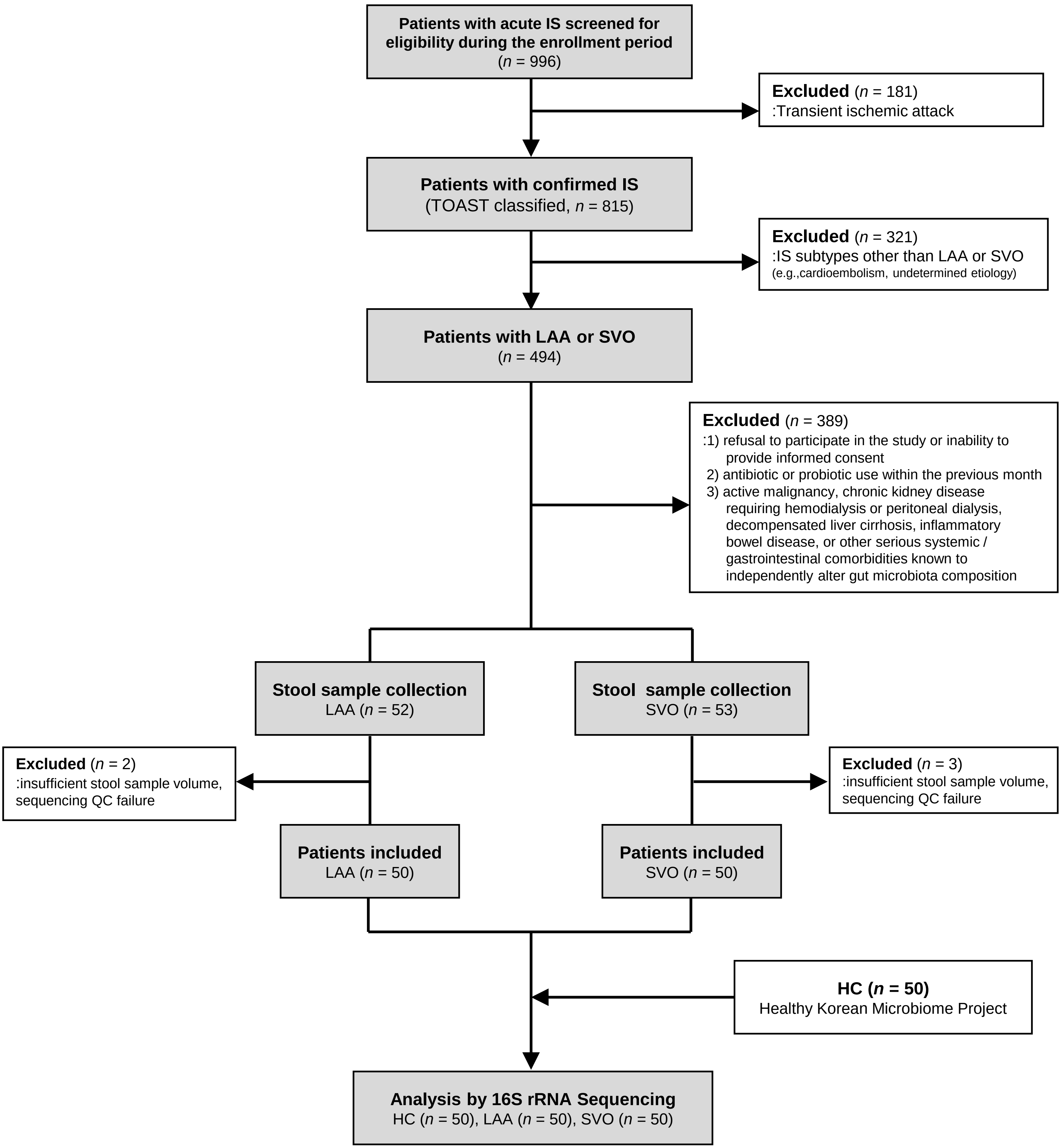
Patients with acute IS screened for eligibility during the enrollment period
(n = 996)
Excluded (n = 181)
:Transient ischemic attack
Patients with confirmed IS
(TOAST classified, n = 815)
Excluded (n = 321)
:IS subtypes other than LAA or SVO
(e.g.,cardioembolism, undetermined etiology)
Patients with LAA or SVO
(n = 494)
Excluded (n = 389)
:1) refusal to participate in the study or inability to provide informed consent
2) antibiotic or probiotic use within the previous month
3) active malignancy, chronic kidney disease requiring hemodialysis or peritoneal dialysis, decompensated liver cirrhosis, inflammatory bowel disease, or other serious systemic / gastrointestinal comorbidities known to independently alter gut microbiota composition
Stool sample collection
LAA (n = 52)
Stool sample collection
SVO (n = 53)
Excluded (n = 2)
:insufficient stool sample volume, sequencing QC failure
Excluded (n = 3)
:insufficient stool sample volume, sequencing QC failure
Patients included
LAA (n = 50)
Patients included
SVO (n = 50)
HC (n = 50)
Healthy Korean Microbiome Project
Analysis by 16S rRNA Sequencing
HC (n = 50), LAA (n = 50), SVO (n = 50)

Supplementary Figure 1. Flow diagram of study participant enrollment.

HC, healthy control; LAA, large-artery atherosclerosis; SVO, small-vessel occlusion.

A

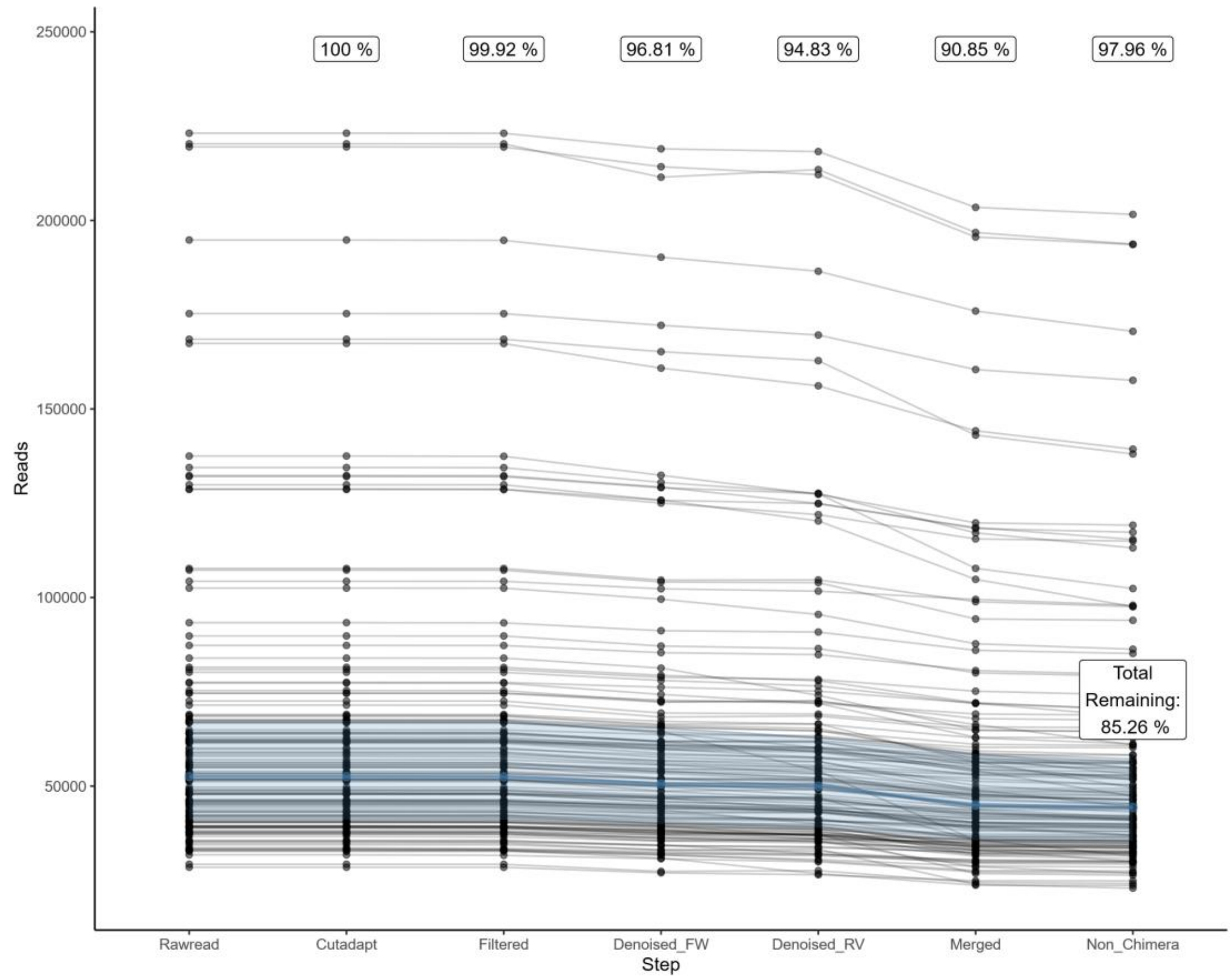

250000
200000
150000
100000
50000
Reads
100 %
99.92 %
96.81 %
94.83 %
90.85 %
97.96 %
Total Remaining: 85.26 %
Rawread
Cutadapt
Filtered
Denoised_FW
Denoised_RV
Merged
Non_Chimera
Step

B

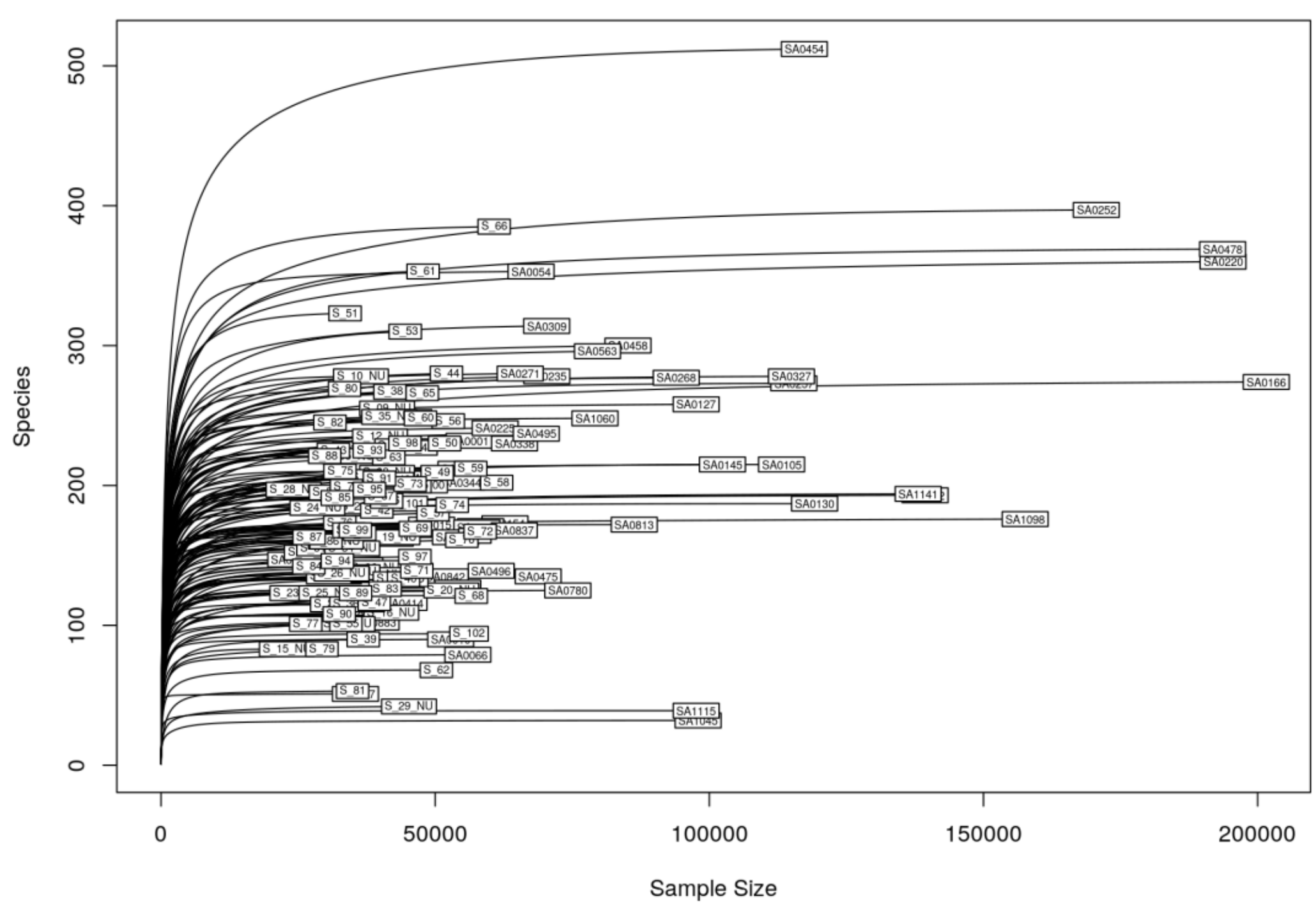

Species
Sample Size
0
50000
100000
150000
200000
100
200
300
400
500
SA0454
SA0252
SA0478
SA0220
SA0166

Supplementary Figure 2. Sequence processing and species richness evaluation.

(A) Sequence retention rates across quality filtering steps. (B) Rarefaction curves showing observed species richness per sample as a function of sequencing depth, indicating sufficient coverage for diversity analyses.

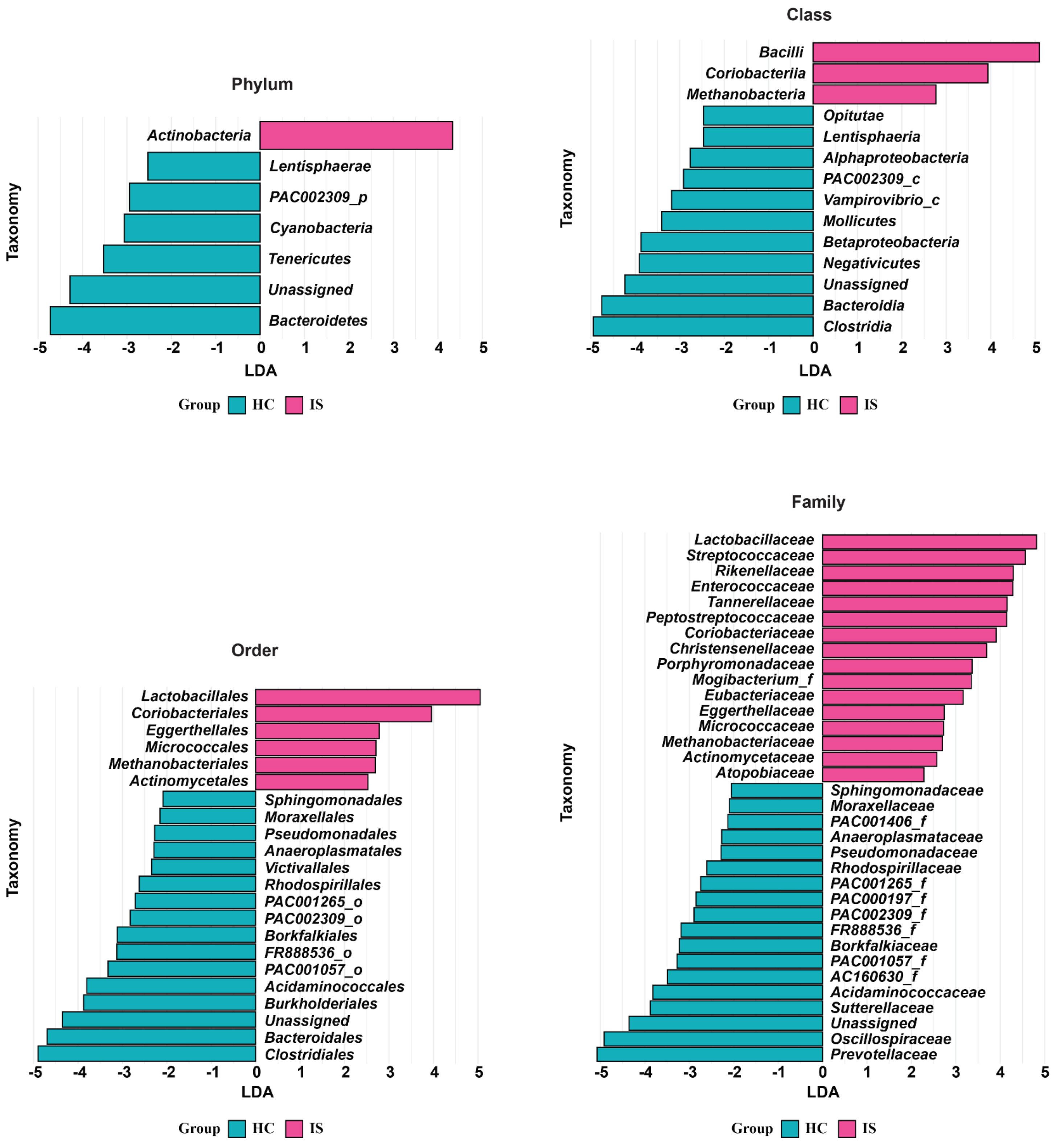

Phylum
Taxonomy
Actinobacteria
Lentisphaerae
PAC002309_p
Cyanobacteria
Tenericutes
Unassigned
Bacteroidetes
-5 -4 -3 -2 -1 0 1 2 3 4 5
LDA
Group HC IS
Class
Taxonomy
Bacilli
Coriobacteriia
Methanobacteria
Opitutae
Lentisphaeria
Alphaproteobacteria
PAC002309_c
Vampirovibrio_c
Mollicutes
Betaproteobacteria
Negativicutes
Unassigned
Bacteroidia
Clostridia
-5 -4 -3 -2 -1 0 1 2 3 4 5
LDA
Group HC IS
Order
Taxonomy
Lactobacillales
Coriobacteriales
Eggerthellales
Micrococcales
Methanobacteriales
Actinomycetales
Sphingomonadales
Moraxellales
Pseudomonadales
Anaeroplasmatales
Victivallales
Rhodospirillales
PAC001265_o
PAC002309_o
Borkfalkiales
FR888536_o
PAC001057_o
Acidaminococcales
Burkholderiales
Unassigned
Bacteroidales
Clostridiales
-5 -4 -3 -2 -1 0 1 2 3 4 5
LDA
Group HC IS
Family
Taxonomy
Lactobacillaceae
Streptococcaceae
Rikenellaceae
Enterococcaceae
Tannerellaceae
Peptostreptococcaceae
Coriobacteriaceae
Christensenellaceae
Porphyromonadaceae
Mogibacterium_f
Eubacteriaceae
Eggerthellaceae
Micrococcaceae
Methanobacteriaceae
Actinomycetaceae
Atopobiaceae
Sphingomonadaceae
Moraxellaceae
PAC001406_f
Anaeroplasmataceae
Pseudomonadaceae
Rhodospirillaceae
PAC001265_f
PAC000197_f
PAC002309_f
FR888536_f
Borkfalkiaceae
PAC001057_f
AC160630_f
Acidaminococcaceae
Sutterellaceae
Unassigned
Oscillospiraceae
Prevotellaceae
-5 -4 -3 -2 -1 0 1 2 3 4 5
LDA
Group HC IS

Supplementary Figure 3. LEfSe analysis of gut microbiome between HC and IS at multiple taxonomic levels.

LEfSe analysis showing taxa enriched in HC (blue) and IS (pink) at the phylum, class, order, and family levels (LDA > 2.0).

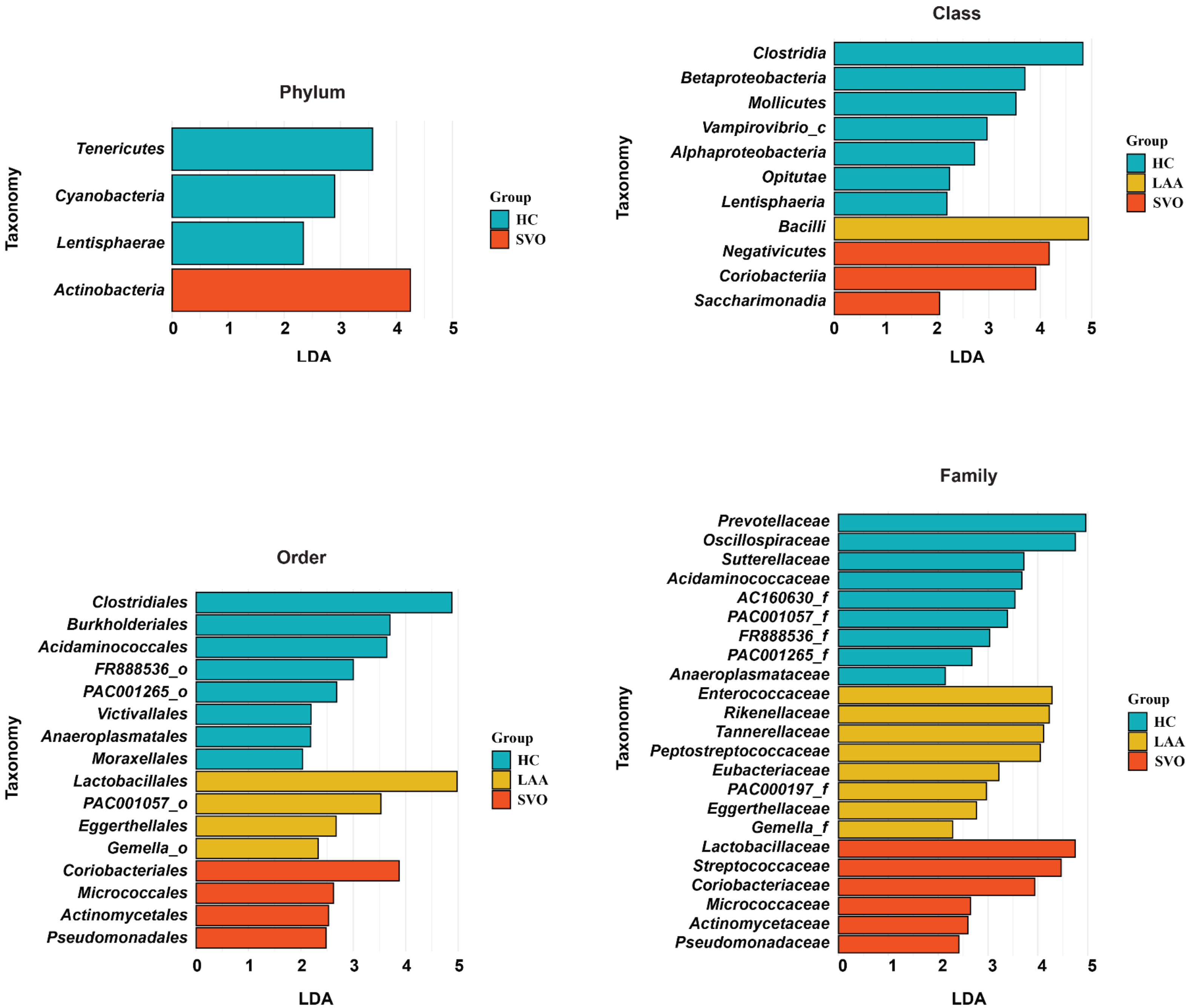

Phylum
Taxonomy
Tenericutes
Cyanobacteria
Lentisphaerae
Actinobacteria
0
1
2
3
4
5
LDA
Group
HC
SVO
Class
Taxonomy
Clostridia
Betaproteobacteria
Mollicutes
Vampirovibrio_c
Alphaproteobacteria
Opitutae
Lentisphaeria
Bacilli
Negativicutes
Coriobacteriia
Saccharimonadia
0
1
2
3
4
5
LDA
Group
HC
LAA
SVO
Order
Taxonomy
Clostridiales
Burkholderiales
Acidaminococcales
FR888536_o
PAC001265_o
Victivallales
Anaeroplasmatales
Moraxellales
Lactobacillales
PAC001057_o
Eggerthellales
Gemella_o
Coriobacteriales
Micrococcales
Actinomycetales
Pseudomonadales
0
1
2
3
4
5
LDA
Group
HC
LAA
SVO
Family
Taxonomy
Prevotellaceae
Oscillospiraceae
Sutterellaceae
Acidaminococcaceae
AC160630_f
PAC001057_f
FR888536_f
PAC001265_f
Anaeroplasmataceae
Enterococcaceae
Rikenellaceae
Tannerellaceae
Peptostreptococcaceae
Eubacteriaceae
PAC000197_f
Eggerthellaceae
Gemella_f
Lactobacillaceae
Streptococcaceae
Coriobacteriaceae
Micrococcaceae
Actinomycetaceae
Pseudomonadaceae
0
1
2
3
4
5
LDA
Group
HC
LAA
SVO

Supplementary Figure 4. LEfSe analysis of gut microbiome across HC, LAA, and SVO at multiple taxonomic levels.

LEfSe analysis showing taxa enriched in HC (blue), LAA (yellow), and SVO (orange) at the phylum, class, order, and family levels (LDA > 2.0). No taxa were significantly enriched in LAA at the phylum level (LDA > 2.0), and therefore only HC and SVO dominant taxa are presented.

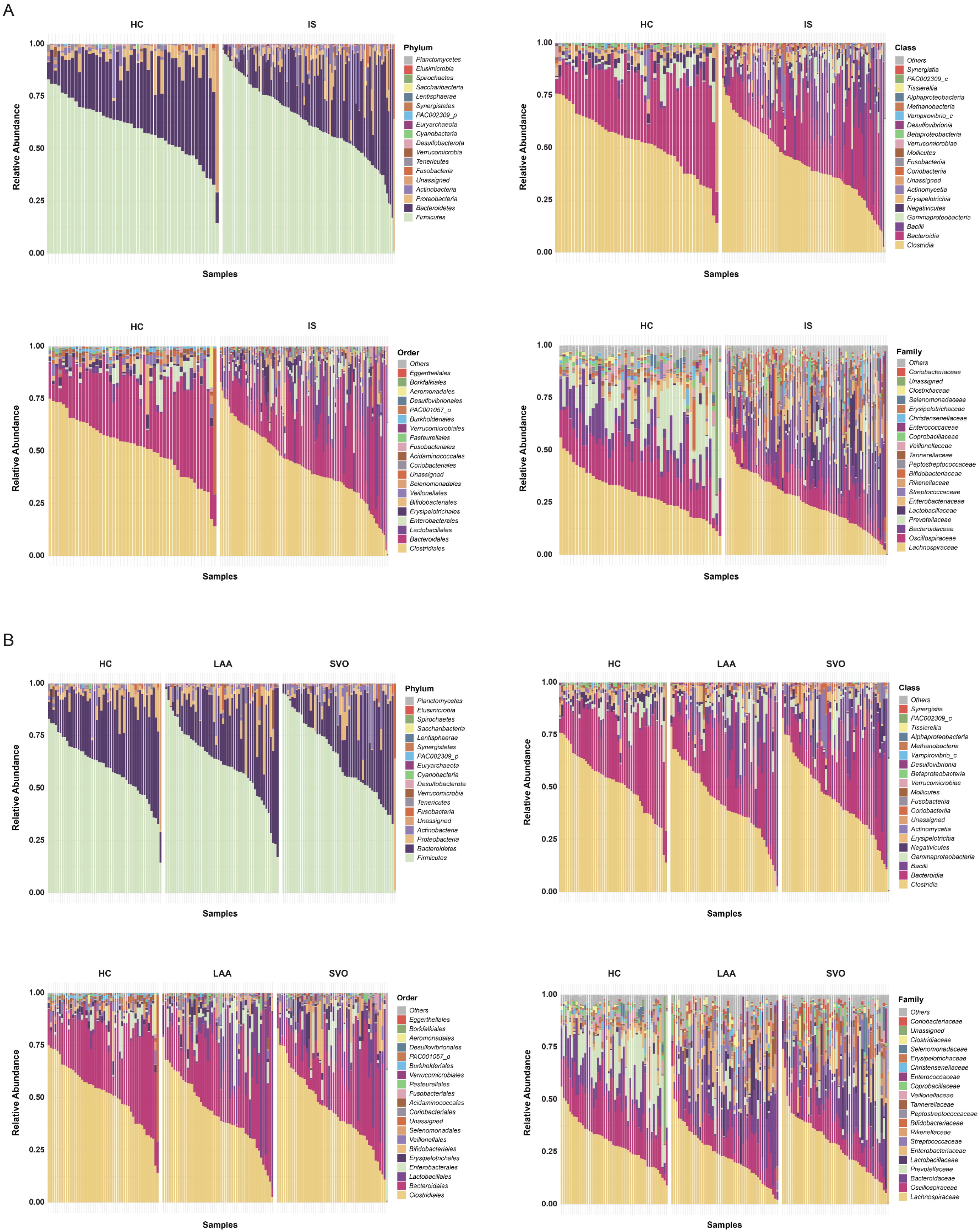
A
HC
IS
Relative Abundance
1.00
0.75
0.50
0.25
0.00
Samples
Phylum
Planctomycetes
Elusimicrobia
Spirochaetes
Saccharibacteria
Lentisphaerae
Synergistetes
PAC002309_p
Euryarchaeota
Cyanobacteria
Desulfobacterota
Verrucomicrobia
Tenericutes
Fusobacteria
Unassigned
Actinobacteria
Proteobacteria
Bacteroidetes
Firmicutes
Class
Others
Synergistia
PAC002309_c
Tissierellia
Alphaproteobacteria
Methanobacteria
Vampirovibrio_c
Desulfovibrionia
Betaproteobacteria
Verrucomicrobiae
Mollicutes
Fusobacteriia
Coriobacteriia
Unassigned
Actinomycetia
Erysipelotrichia
Negativicutes
Gammaproteobacteria
Bacilli
Bacteroidia
Clostridia
Order
Others
Eggerthellales
Borkfalkiales
Aeromonadales
Desulfovibrionales
PAC001057_o
Burkholderiales
Verrucomicrobiales
Pasteurellales
Fusobacteriales
Acidaminococcales
Coriobacteriales
Unassigned
Selenomonadales
Veillonellales
Bifidobacteriales
Erysipelotrichales
Enterobacterales
Lactobacillales
Bacteroidales
Clostridiales
Family
Others
Coriobacteriaceae
Unassigned
Clostridiaceae
Selenomonadaceae
Erysipelotrichaceae
Christensenellaceae
Enterococcaceae
Coprobacillaceae
Veillonellaceae
Tannerellaceae
Peptostreptococcaceae
Bifidobacteriaceae
Rikenellaceae
Streptococcaceae
Enterobacteriaceae
Lactobacillaceae
Prevotellaceae
Bacteroidaceae
Oscillospiraceae
Lachnospiraceae
B
HC
LAA
SVO

Supplementary Figure 5. Taxonomic composition of gut microbiome at multiple taxonomic levels.

(A) Relative abundance at the phylum, class, order, and family levels in HC and IS. Each bar represents one sample. (B) Relative abundance at the phylum, class, order, and family levels across HC, LAA, and SVO. Each bar represents one sample.

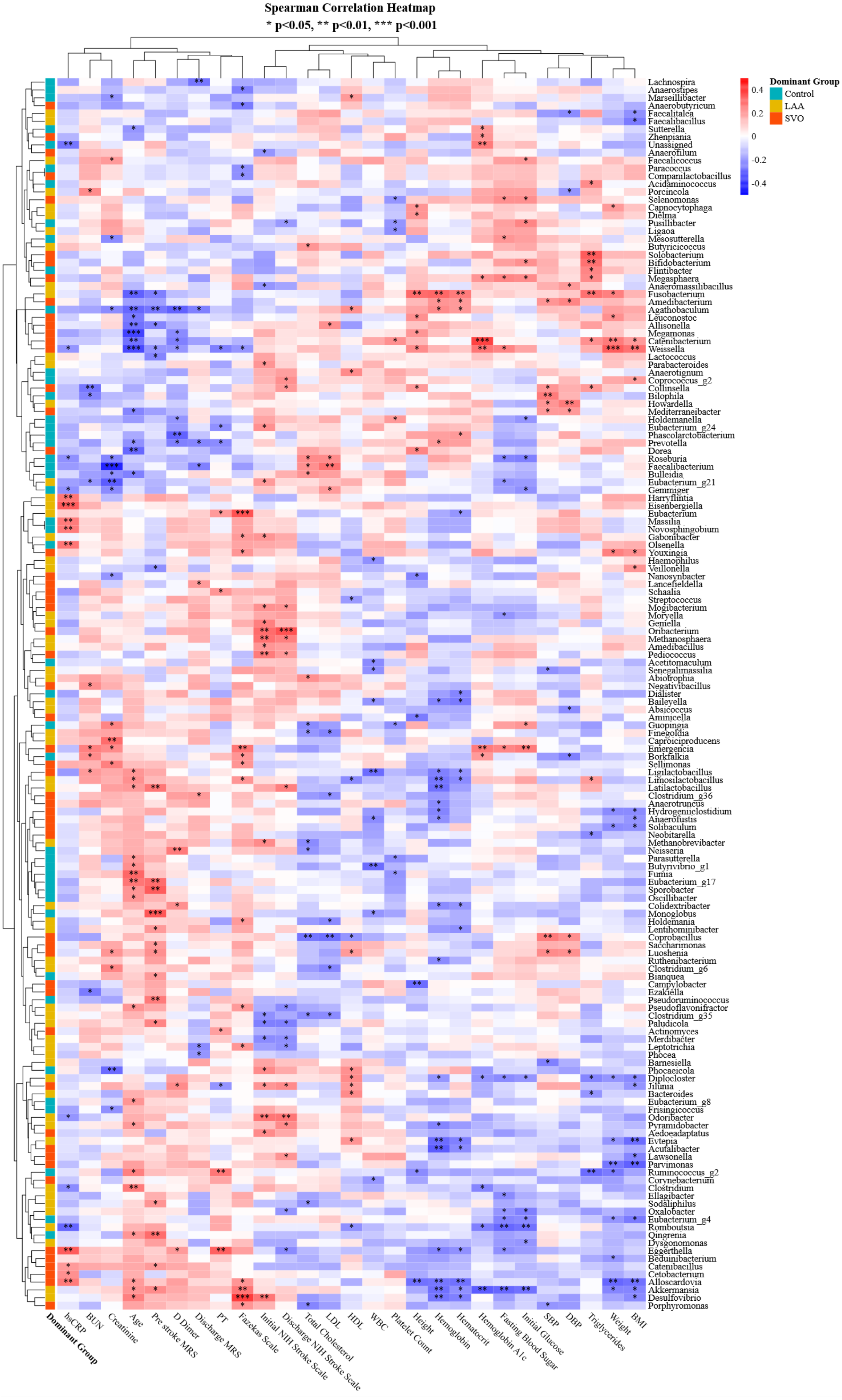
Spearman Correlation Heatmap
* p<0.05, ** p<0.01, *** p<0.001
Dominant Group
Control
LAA
SVO
0.4
0.2
0
-0.2
-0.4
Lachnospira
Anaerostipes
Marseillibacter
Anaerobutyricum
Faecalitalea
Faecalibacillus
Sutterella
Zhenpiania
Unassigned
Anaerofilum
Faecalicoccus
Paracoccus
Companilactobacillus
Acidaminococcus
Porcincola
Selenomonas
Capnocytophaga
Dielma
Pusillibacter
Ligaoa
Mesosutterella
Butyricicoccus
Solobacterium
Bifidobacterium
Flintibacter
Megasphaera
Anaeromassilibacillus
Fusobacterium
Amedibacterium
Agathobaculum
Leuconostoc
Allisonella
Megamonas
Catenibacterium
Weissella
Lactococcus
Parabacteroides
Anaerotignum
Coprococcus_g2
Collinsella
Bilophila
Howardella
Mediterraneibacter
Holdemanella
Eubacterium_g24
Phascolarctobacterium
Prevotella
Dorea
Roseburia
Faecalibacterium
Bulleidia
Eubacterium_g21
Gemmiger
Harryflintia
Eisenbergiella
Eubacterium
Massilia
Novosphingobium
Gabonibacter
Olsenella
Youxingia
Haemophilus
Veillonella
Nanosynbacter
Lancefieldella
Schaalia
Streptococcus
Mogibacterium
Moryella
Gemella
Oribacterium
Methanosphaera
Amedibacillus
Pediococcus
Acetitomaculum
Senegalimassilia
Abiotrophia
Negativibacillus
Dialister
Baileyella
Absicoccus
Aminicella
Guopingia
Finegoldia
Caproiciproducens
Emergencia
Borkfalkia
Sellimonas
Ligilactobacillus
Limosilactobacillus
Latilactobacillus
Clostridium_g36
Anaerotruncus
Hydrogeniiclostidium
Anaerofustis
Solibaculum
Neobitarella
Methanobrevibacter
Neisseria
Parasutterella
Butyrivibrio_g1
Fumia
Eubacterium_g17
Sporobacter
Oscillibacter
Colidextribacter
Monoglobus
Holdemania
Lentihominibacter
Coprobacillus
Saccharimonas
Luoshenia
Ruthenibacterium
Clostridium_g6
Bianquea
Campylobacter
Ezakiella
Pseudoruminococcus
Pseudoflavonifractor
Clostridium_g35
Paludicola
Actinomyces
Merdibacter
Leptotrichia
Phocea
Barnesiella
Phocaeicola
Diplocloster
Jilunia
Bacteroides
Eubacterium_g8
Frisingicoccus
Odoribacter
Pyramidobacter
Aedoeadaptatus
Evtepia
Acutalibacter
Lawsonella
Parvimonas
Ruminococcus_g2
Corynebacterium
Clostridium
Ellagibacter
Sodaliphilus
Oxalobacter
Eubacterium_g4
Romboutsia
Qingrenia
Dysgonomonas
Eggerthella
Beduinibacterium
Catenibacillus
Cetobacterium
Alloscardovia
Akkermansia
Desulfovibrio
Porphyromonas
Dominant Group
hsCRP
BUN
Creatinine
Age
Pre stroke MRS
D Dimer
Discharge MRS
PT
Fazekas Scale
Initial NIH Stroke Scale
Discharge NIH Stroke Scale
Total Cholesterol
LDL
HDL
WBC
Platelet Count
Height
Hemoglobin
Hematocrit
Hemoglobin A1c
Fasting Blood Sugar
Initial Glucose
SBP
DBP
Triglycerides
Weight
BMI

Supplementary Figure 6. Correlations between clinical parameters and LEfSe-identified taxa (LDA > 2.0) in IS.

Spearman correlation coefficients between genus-level gut microbial taxa and clinical parameters in IS are displayed using genera identified from a three-group LEfSe analysis (HC, LAA, SVO; LDA > 2.0), including only genera detected in IS patients. The side bar indicates the LEfSe-dominant group (HC, LAA, SVO). Heatmap colors represent the strength and direction of the associations (red, positive; blue, negative). Statistical significance is indicated as follows: *$p < 0.05$, **$p < 0.01$, ***$p < 0.001$.
