# Supplementary material for: Subtype-associated gut microbial taxa and their clinical correlates in acute ischemic stroke: comparison of large-artery atherosclerosis and small-vessel occlusion
Source: Front Microbiol. 2026 Sep 11;17:1867057. doi: 10.3389/fmicb.2026.1867057 (PMC13613636; doi:10.3389/fmicb.2026.1867057)
Supplement: Supplementary file 2 [file Data_Sheet_2.pdf]

IS(LAA)							
Sample	Rawread	Cutadapt	Filtered	Denoised_FW	Denoised_RV	Merged	Non chimera
S_01	43735	43735	43724	42801	40901	39002	38649
S_05	40481	40481	40470	39411	36744	34717	34397
S_07	46454	46454	46448	45270	43836	41349	40991
S_08	32692	32692	32683	31804	30211	27647	27509
S_13	51194	51194	51179	49737	47669	44350	43960
S_15	34876	34876	34868	33037	33309	23891	22889
S_18	51763	51763	51743	49932	49356	44669	44377
S_20	58382	58382	58328	56708	55515	53314	51838
S_24	32970	32970	32961	31687	30562	28791	28542
S_25	35441	35441	35427	34413	32039	30349	30319
S_26	37701	37701	37687	36239	35292	32975	32930
S_27	48100	48100	48083	46760	45410	43813	43711
S_28	29279	29279	29273	27371	27600	24434	24175
S_32	45803	45803	45791	44131	43672	41282	41167
S_34	41058	41058	41048	39466	38602	35400	34914
S_36	38937	38937	38914	37622	37094	35890	35378
S_42	43099	43099	43092	42036	41000	39458	39315
S_44	62223	62223	62221	59859	59581	53838	52026
S_45	53540	53540	53534	51731	51693	47609	46261
S_46	48763	48763	48757	47062	47093	43402	41173
S_47	42305	42305	42301	41114	40758	39428	38926
S_48	51688	51688	51685	50071	49755	44952	43121
S_49	56992	56992	56986	55623	54981	51379	50483
S_52	40276	40276	40273	38698	37952	32872	29768
S_58	69012	69012	69001	67182	66453	62748	61167
S_61	59036	59036	59026	56695	56524	49621	47173
S_62	56158	56158	56146	54630	53933	52231	50381
S_63	49719	49719	49717	48550	46877	43162	42695
S_66	67735	67735	67727	65789	64587	61062	60737
S_68	62912	62912	62904	60670	60310	57099	56547
S_71	53276	53276	53272	52094	51268	47154	46705
S_75	39274	39274	39080	37835	37317	32902	32581
S_76	36654	36654	36493	35461	34934	33225	32595
S_78	38316	38316	38182	37059	36839	34246	33694
S_79	33626	33626	33460	32659	32043	29490	29344
S_80	38963	38963	38801	37296	36995	33797	33478
S_81	37268	37268	37144	36374	36041	35096	34951
S_82	37957	37957	37796	36273	35386	31520	30844
S_83	48329	48329	48161	47075	46548	42751	40946
S_84	37624	37624	37473	35781	35451	28618	26980
S_86	33284	33284	33159	32210	31787	30320	29969
S_87	31782	31782	31640	30728	29927	27541	27308
S_89	39510	39510	39330	38299	37576	35961	35491
S_92	41660	41660	41620	40145	39156	34370	34251
S_93	47851	47851	47791	45872	44548	38386	37964
S_95	43103	43103	43024	41602	40836	38179	38039
S_96	41840	41840	41782	40085	39972	36290	36174
S_99	48872	48872	48814	47190	46850	35938	35739
S_100	56336	56336	56223	53753	52424	48469	48275
S_102	63766	63766	63676	61742	59117	56396	56301

Supplementary Table 1. Sequencing read processing summary in LAA

Read counts at each processing step for LAA, including raw reads, adapter trimming (Cutadapt), quality filtering, denoising (forward and reverse), merging, and chimera removal, are presented for each sample.

IS(SVO)							
Sample	Rawread	Cutadapt	Filtered	Denoised_FW	Denoised_RV	Merged	Non chimera
S_02	39432	39432	39419	38313	36877	31716	31508
S_03	39289	39289	39278	38080	37046	35528	35435
S_06	48046	48046	48036	46775	43907	40438	39090
S_09	49146	49146	49128	47637	46382	42965	41254
S_10	40566	40566	40559	39524	38466	36532	36426
S_12	45980	45980	45966	44751	43151	40212	39929
S_14	45081	45081	45066	43926	43264	39926	39554
S_16	45862	45862	45857	44871	44352	42821	41993
S_17	37862	37862	37854	36930	36101	35048	34926
S_19	47844	47844	47832	46636	46167	42366	42281
S_22	41118	41118	41113	39898	38963	37223	37163
S_23	28495	28495	28482	26959	26513	24882	24847
S_29	51836	51836	51813	48695	49151	46627	45202
S_30	35636	35636	35621	34194	33959	32276	32257
S_31	34423	34423	34412	33131	32735	29876	29751
S_33	39170	39170	39159	37363	37260	33993	33964
S_35	47816	47816	47791	45901	44725	41832	41302
S_37	40609	40609	40594	39178	38663	32003	31901
S_38	53259	53259	53182	50770	50770	43800	41692
S_39	44388	44388	44386	42907	42993	39033	37053
S_40	51855	51855	51849	50408	49045	45440	44274
S_41	51481	51481	51477	50079	49692	47545	47261
S_43	43332	43332	43329	41414	41461	35826	31465
S_50	64086	64086	64080	61986	61763	55442	51895
S_51	42135	42135	42125	40182	39311	33955	33509
S_53	60097	60097	60087	57596	56634	49029	44633
S_54	44875	44875	44873	43270	43007	39338	37112
S_55	39386	39386	39356	38364	36075	34115	33820
S_56	62265	62265	62257	60157	59912	54492	52396
S_57	63997	63997	63990	61703	61728	56458	49583
S_59	67411	67411	67403	65317	64710	58260	56515
S_60	61620	61620	61613	59521	59009	54032	48671
S_64	66772	66772	66759	65125	62801	58526	57065
S_65	55736	55736	55728	53998	52168	48315	47715
S_67	45339	45339	45333	44210	43693	40458	40035
S_69	53894	53894	53888	51985	51918	47641	46370
S_70	59803	59803	59795	58282	57686	55188	54157
S_72	62354	62354	62347	61147	60389	58467	58286
S_73	54920	54920	54918	53289	51696	47742	45245
S_74	68515	68515	68511	66097	65349	57359	53123
S_77	38969	38969	38817	37751	37117	26841	26397
S_85	40481	40481	40324	39040	38126	33633	32024
S_88	33369	33369	33211	32073	31565	29880	29860
S_90	35269	35269	35125	34242	33744	32674	32538
S_91	46260	46260	46195	44787	43872	40441	39886
S_94	37258	37258	37209	35413	35865	32204	32159
S_97	52516	52516	52453	50878	50523	46664	45784
S_98	52688	52688	52617	50614	50300	44908	44534
S_101	57928	57928	57790	55718	53344	46216	45013
S_103	42274	42274	42265	40651	39503	34493	34229

Supplementary Table 2. Sequencing read processing summary in SVO

Read counts at each processing step for SVO, including raw reads, adapter trimming (Cutadapt), quality filtering, denoising (forward and reverse), merging, and chimera removal, are presented for each sample.

HC							
Sample	Rawread	Cutadapt	Filtered	Denoised_FW	Denoised_RV	Merged	Non chimera
SA0001	61830	61830	61799	60611	59315	56539	56144
SA0015	54954	54954	54939	53817	53279	49435	49377
SA0016	57035	57035	56997	55849	54431	51475	51371
SA0028	58624	58624	58615	57268	56089	52685	52376
SA0031	61866	61866	61856	60822	59985	57719	52251
SA0044	141442	141442	141409	136787	131565	120601	118913
SA0054	75328	75328	75318	72357	72582	67889	67204
SA0099	100371	100371	100361	97270	96912	90496	90074
SA0105	134477	134477	134449	130467	127640	116879	112720
SA0127	107719	107719	107710	104682	104721	98964	97665
SA0130	137526	137526	137460	132377	127332	119671	119114
SA0145	132077	132077	132042	128833	127630	108003	102708
SA0150	58847	58847	58829	56102	55066	48547	48368
SA0154	77314	77314	77291	74295	71967	62960	62458
SA0166	223129	223129	223107	218920	218300	202840	200977
SA0179	131194	131194	131124	127560	124509	116895	112391
SA0220	220313	220313	220283	211451	213445	196702	193316
SA0225	74651	74651	74634	72224	72618	66325	60952
SA0228	199001	199001	198953	192917	189957	179093	174103
SA0235	81513	81513	81483	79309	77951	72113	70587
SA0239	250626	250626	250598	242367	241443	223152	221098
SA0242	102397	102397	102389	100579	99448	93438	92808
SA0247	318688	318688	318516	310580	305790	286384	275521
SA0252	194799	194799	194714	190195	186525	175934	171076
SA0257	132363	132363	132310	129258	125004	118574	115965
SA0268	107267	107267	107243	104121	104009	94292	93296
SA0271	72566	72566	72552	69382	69067	65662	65489
SA0309	80180	80180	80150	78003	76602	71902	70455
SA0321	64912	64912	64902	63658	63318	59353	58268
SA0327	128673	128673	128628	125042	121991	115481	114899
SA0338	71473	71473	71446	68286	68640	64790	64409
SA0344	64292	64292	64263	61799	62040	58468	54876
SA0454	128771	128771	128733	125787	124937	118327	117354
SA0458	93338	93338	93297	91112	90882	86030	85222
SA0478	219498	219498	219466	214250	212335	195653	193645
SA0495	77524	77524	77491	76231	75180	71974	67053
SA0548	220988	220988	219123	218614	218809	216301	65428
SA0562	74779	74779	74753	73071	73231	70250	69962
SA0563	87311	87311	87281	85380	84866	80622	79514
SA0577	109672	109672	109593	106284	104044	99172	97249
SA0632	167380	167380	167349	160752	156049	144034	139174
SA0656	189702	189702	189596	183584	183654	163399	161433
SA0693	171941	171941	171920	165536	160171	146830	146471
SA0792	67390	67390	67373	64002	60265	54519	53831
SA0813	102509	102509	102498	99566	95345	87586	86167
SA0837	83947	83947	83943	81270	74127	64868	64379
SA1060	89821	89821	89816	87091	86639	80453	79408
SA1098	175291	175291	175275	172252	169672	160562	158753
SA1104	78718	78718	78710	76493	72893	66501	66245
SA1141	168497	168497	168486	165205	162888	143031	137375

Supplementary Table 3. Sequencing read processing summary in HC

Read counts at each processing step for HC, including raw reads, adapter trimming (Cutadapt), quality filtering, denoising (forward and reverse), merging, and chimera removal, are presented for each sample.

	Age	WBC	Total Cholesterol	BUN	Creatinine	Hemoglobin	Triglycerides	Hematocrit	HDL	Fasting Blood Sugar	Platelet Count	LDL	Hemoglobin A1c	PT	hsCRP	Initial Glucose	D-Dimer	SBP	DBP	Height	Weight	BMI	Initial NIH Stroke Scale	Discharge NIH Stroke Scale	Pre-stroke MRS	Discharge MRS	Fazekas Scale
Methanobrevibacter	0.16	-0.144	-0.217	0.059	0.124	-0.171	-0.097	-0.144	-0.03	-0.014	-0.038	-0.142	0.017	-0.064	-0.049	0.002	0.165	-0.094	-0.07	0.051	0	-0.138	0.229	0.068	0.105	0.138	0.179
Methanosphaera	0.042	-0.068	-0.007	0.151	0.062	-0.195	-0.003	-0.195	-0.015	0.093	-0.06	0.016	0.079	-0.059	-0.013	0.069	0.04	0.041	0.003	0.075	0.121	0.023	0.285	0.248	0.04	0.161	0.001
Nanosynbacter	-0.033	-0.085	0.126	-0.073	-0.207	0.004	0.019	-0.018	0.003	-0.162	0.03	0.132	-0.098	0.059	-0.085	-0.156	-0.041	0.12	0.137	-0.236	-0.084	0.034	0.089	0.128	-0.006	0.078	0.107
Saccharimonas	0.038	0.138	-0.11	0.113	0.167	-0.031	-0.165	-0.087	0.159	0.078	0.07	-0.131	-0.162	0.028	-0.042	0.084	-0.026	0.162	0.152	0.171	0.172	-0.169	0.028	0.048	0.205	0.053	0.18
Campylobacter	0.117	0.157	-0.025	-0.153	-0.048	0.034	0.1	0.012	-0.1	-0.003	0.088	-0.018	0.013	0.077	-0.123	0.051	0.072	-0.073	-0.073	-0.288	-0.073	0.025	0.009	-0.026	0.177	-0.059	0.021
Fusobacterium	-0.306	0.113	-0.037	-0.09	0.082	0.303	0.284	0.262	-0.181	0.099	0.055	-0.048	0.066	-0.012	-0.04	0.066	-0.123	0.081	0.108	0.259	0.239	0.172	-0.01	-0.032	-0.241	-0.092	-0.094
Leptotrichia	-0.04	0.028	-0.073	0.048	0.13	0.048	-0.043	0.035	-0.194	0.084	-0.065	-0.015	-0.027	0.024	-0.074	0.094	0.094	0.052	-0.035	-0.135	-0.092	-0.011	-0.182	-0.216	0.014	-0.225	0.214
Cetobacterium	0.164	-0.01	0.023	-0.146	-0.074	-0.11	-0.059	-0.103	0.005	-0.131	0.169	-0.042	-0.045	0.092	0.221	-0.124	0.089	-0.134	-0.058	-0.166	-0.162	-0.106	0.028	0.048	-0.068	0.155	0.01
Akkermansia	0.213	-0.015	0.066	0.061	0.088	-0.272	0.003	-0.208	-0.009	-0.294	0.015	0.091	-0.315	0.13	-0.049	-0.275	0.177	-0.072	-0.056	-0.16	-0.219	-0.256	0.07	0.105	0.237	0.134	0.272
Prevotella	-0.211	0.113	0.064	-0.185	-0.05	0.198	0.09	0.192	-0.05	-0.186	0.111	0.093	0.058	-0.223	-0.09	-0.18	-0.241	0.067	0.028	0.014	0.138	0.161	0.106	-0.05	-0.098	-0.234	-0.028
Capnocytophaga	-0.005	-0.047	-0.145	0.023	0.137	0.031	0.01	0.07	-0.184	0.108	-0.108	-0.088	0.055	0.028	-0.06	0.101	-0.086	0.006	-0.088	0.2	0.202	0.119	0.04	-0.023	-0.096	-0.008	0.137
Lancefieldella	0.074	-0.04	0.092	0.144	-0.086	0.033	0.033	0.09	0.063	-0.006	-0.071	0.109	-0.021	0.014	0.007	0.012	-0.013	0.117	0.078	-0.189	-0.038	0.081	0.074	0.188	-0.096	0.204	-0.02
Olsenella	0.038	0.148	-0.155	0.045	0.068	-0.172	-0.038	-0.172	-0.066	-0.028	0.165	-0.143	0.08	0.112	0.272	0.016	0.127	-0.173	-0.173	-0.078	0.141	0.162	0.174	0.164	-0.068	0.155	0.18
Collinsella	-0.164	0.083	0.076	-0.261	-0.008	-0.014	0.201	0.003	-0.02	-0.034	0.095	0.064	0.07	-0.113	-0.118	-0.037	-0.147	0.216	0.163	0.153	0.106	0.004	0.122	0.205	-0.013	0.048	0.068
Senegalimassilia	-0.076	-0.217	-0.064	0.015	0.078	-0.161	0.001	-0.107	0.025	-0.067	0.084	0.012	0.06	-0.084	-0.025	-0.13	0.105	-0.208	-0.181	-0.072	-0.068	-0.059	0.184	0.143	-0.102	0.044	0.187
Ellagibacter	0.023	0.107	-0.171	-0.156	-0.018	-0.064	-0.008	-0.103	0.076	-0.222	0.043	-0.127	-0.047	0.058	-0.106	-0.154	0.066	-0.114	-0.133	0.041	-0.057	-0.112	0.022	-0.071	0.162	-0.152	0.174
Gordonibacter	-0.133	0.075	0.005	0.029	0.149	0.083	0.057	0.089	-0.118	0.131	0.004	0.028	0.124	-0.013	0.08	0.107	-0.129	0.028	0.109	0.242	0.078	-0.027	-0.097	-0.017	-0.013	-0.015	-0.109
Eggerthella	0.135	-0.103	-0.134	0.123	0.106	-0.237	-0.099	-0.211	-0.122	-0.214	-0.016	-0.085	-0.067	0.314	0.323	-0.194	0.206	-0.143	-0.106	-0.145	-0.131	-0.079	-0.065	-0.223	0.098	-0.224	0.188
Lawsonella	0.082	0.181	-0.067	-0.08	0.102	-0.126	0	-0.106	0.023	0.007	0.188	-0.104	-0.036	-0.063	0.06	0.03	0.127	0.074	0.046	-0.025	-0.141	-0.218	0.121	0.217	0.081	0.062	0.015
Corynebacterium	0.069	-0.2	0.053	0.023	-0.099	-0.02	-0.191	-0.052	-0.119	-0.028	-0.02	0.071	-0.091	0.185	-0.106	-0.039	0.033	-0.163	-0.156	-0.183	-0.182	-0.091	0.1	0.012	-0.013	0.092	0.024
Actinomyces	0.086	0.018	0.05	0.139	0.126	-0.022	-0.066	-0.028	-0.097	-0.039	-0.056	0.009	0.002	0.197	-0.036	-0.045	-0.014	0.004	-0.052	-0.084	-0.072	-0.022	-0.153	-0.168	0.051	-0.119	0.021
Schaalia	0.052	-0.137	-0.014	0.161	0.087	-0.077	0.052	-0.108	-0.094	-0.042	-0.044	0.078	-0.049	0.216	-0.184	-0.037	-0.01	0.023	0.058	-0.095	-0.037	-0.038	0.162	0.163	0.005	0.083	0.156
Bifidobacterium	-0.149	0.069	0.119	-0.122	-0.012	-0.111	0.288	-0.123	0.045	0.188	-0.01	0.046	0.057	0.019	-0.041	0.223	-0.031	0.16	0.052	-0.007	0.062	0.116	-0.194	0.016	-0.002	-0.094	-0.159
Allscardovia	0.206	0.039	-0.075	0.09	-0.057	-0.261	-0.002	-0.276	-0.148	-0.051	0.053	-0.011	-0.072	0.067	0.264	-0.048	0.196	-0.106	-0.15	-0.287	-0.292	-0.252	0.066	-0.029	0.113	0.076	0.215
Pyramidobacter	0.2	0.113	0.102	0.041	0.065	-0.228	-0.04	-0.194	0.146	-0.103	-0.003	0.044	-0.137	0.061	-0.042	-0.076	0.158	-0.052	-0.163	-0.176	-0.108	-0.116	0.135	0.233	0.082	0.1	0.092
Parasutterella	0.206	-0.059	-0.067	0.111	-0.132	-0.015	-0.049	-0.003	-0.078	0.04	-0.211	-0.033	0.024	0.042	-0.06	0.056	0.013	-0.159	-0.125	-0.198	-0.102	0.036	-0.038	-0.07	0.166	-0.078	-0.003
Sutterella	-0.197	0.077	0.113	0.082	-0.023	0.073	0.063	0.003	-0.141	0.045	-0.003	0.127	0.199	-0.134	-0.053	0.05	-0.137	-0.096	0.011	0.107	0.013	-0.053	-0.095	-0.081	-0.074	-0.162	-0.054
Mesosutterella	0.064	0.053	0.088	0.019	-0.225	0.025	0.091	0.024	-0.026	0.208	0.036	0.06	0.152	-0.171	0.064	0.192	0.097	0.13	0.104	-0.167	-0.071	0.045	-0.01	0.04	-0.026	0.093	-0.068
Massilia	0.012	0.03	-0.016	0.089	0.125	-0.162	0.091	-0.165	-0.103	-0.003	0.117	0.021	0.012	-0.017	0.266	0.03	0.138	0.134	0.149	-0.078	-0.089	-0.042	0.081	0.087	-0.068	0.115	0.18
Oxalobacter	0.094	-0.079	0.005	0.025	0.058	0.016	-0.188	0.042	-0.029	-0.203	-0.16	0.028	-0.088	0.036	-0.06	-0.24	0.047	-0.106	-0.115	0.003	-0.137	-0.186	0.015	-0.203	0.063	-0.16	0.015
Neisseria	0.164	-0.121	-0.201	-0.004	0.129	-0.015	-0.124	-0.005	-0.042	0.078	-0.066	-0.147	0.027	-0.01	-0.085	0.098	0.266	0.004	-0.033	0.025	-0.082	-0.128	0.127	0.134	0.121	0.019	0.11
Haemophilus	-0.047	-0.213	0.023	-0.044	0.017	-0.065	-0.048	-0.116	-0.123	-0.171	-0.007	0.162	-0.116	0.06	-0.087	-0.186	0.058	-0.009	0.012	-0.033	0.035	0.087	-0.003	0.038	-0.066	-0.04	0.028
Bilophila	-0.118	0.14	0.011	-0.235	-0.133	0.117	-0.089	0.146	0.06	-0.007	-0.02	-0.06	-0.009	0.025	-0.125	-0.002	-0.158	0.264	0.149	-0.017	-0.052	-0.122	0.154	0.03	0.062	-0.012	0.114
Desulfovibrio	0.254	-0.025	-0.195	-0.028	0.064	-0.259	-0.056	-0.242	-0.063	-0.184	0.048	-0.149	0.007	-0.007	-0.015	-0.09	0.149	-0.115	-0.188	-0.099	-0.166	-0.225	0.272	0.113	0.144	0.133	0.387
Paracoccus	-0.03	-0.002	0.002	0.165	0.068	0.085	0.111	0.071	-0.122	0.12	0.096	0.04	0.127	-0.017	-0.042	0.131	-0.042	0.056	-0.058	-0.054	0.092	0.148	0.081	0.087	-0.068	-0.065	-0.207
Novosphingobium	0.012	0.03	-0.016	0.089	0.125	-0.162	0.091	-0.165	-0.103	-0.003	0.117	0.021	0.012	-0.017	0.266	0.03	0.138	0.134	0.149	-0.078	-0.089	-0.042	0.081	0.087	-0.068	0.115	0.18
Romboutsia	0.149	-0.127	-0.031	-0.05	0.019	-0.068	0.054	-0.041	-0.212	-0.289	-0.072	0.061	-0.241	0.172	-0.288	-0.275	0.018	0.029	-0.01	-0.135	-0.127	-0.077	-0.031	-0.028	0.183	-0.039	-0.006
Bulleidia	-0.243	0.093	0.21	-0.181	-0.215	0.019	-0.076	0.038	0.064	-0.13	0.194	0.192	-0.071	0.01	-0.074	-0.155	0.01	0.001	0.014	-0.128	-0.022	0.044	0.059	0.16	-0.118	0.081	-0.088
Solobacterium	-0.146	0.151	0.098	-0.035	-0.009	0.165	0.319	0.102	-0.183	0.069	-0.038	0.04	0.185	-0.069	-0.123	0.075	-0.09	0.139	0.089	0.01	0.14	0.149	-0.185	0.047	0.03	-0.008	-0.037
Holdemania	0.114	-0.091	-0.196	-0.063	0.012	-0.143	-0.012	-0.15	0.055	-0.055	-0.073	-0.225	0.004	-0.18	-0.133	-0.009	0.099	-0.03	-0.158	0.135	-0.049	-0.093	-0.014	-0.011	0.089	0.092	0.201
Dielma	0.011	-0.025	-0.02	0.038	0.12	0.003	-0.077	0.032	-0.016	0.106	-0.103	0.048	0.147	0.005	-0.074	0.054	-0.135	0.087	-0.059	0.204	0.142	0.092	0.01	-0.043	-0.118	0.026	0.119
Clostridium_g36	0.135	-0.102	-0.191	0.127	0.154	-0.193	-0.063	-0.129	0.034	0.09	-0.08	-0.217	-0.067	-0.001	-0.031	0.125	0.187	0.067	-0.012	-0.098	-0.045	-0.018	0.049	0.015	0.018	0.206	0.13
Merdibacter	-0.015	0.038	0.02	0.131	0.055	0.012	-0.192	0.037	-0.057	0.018	-0.012	-0.011	0.074	0.11	0.01	-0.021	0.031	0.018	-0.01	0.098	-0.103	-0.156	-0.223	-0.212	0.072	-0.088	-0.059
Faecalicoccus	-0.023	0.193	-0.003	0.17	0.203	-0.005	0.023	-0.012	0.154	0.193	0.094	-0.072	0.069	-0.082	-0.096	0.199	-0.057	0.086	-0.004	0.18	0.171	-0.046	0.126	0.158	-0.03	-0.095	-0.063
Absicoccus	0.137	0.019	-0.01	0.14	-0.004	-0.161	0.06	-0.163	-0.02	0.018	-0.111	-0.007	-0.033	0.126	-0.085	0.027	-0.019	-0.11	-0.223	-0.152	-0.035	0.08	0.139	0.099	-0.047	-0.041	0.106
Faecalitalea	0.038	0.088	0.16	0.053	0.026	0.055	0.189	0.056	-0.03	0.072	-0.047	0.193	0.09	-0.023	-0.085	0.098	-0.137	-0.191	-0.225	0.039	-0.131	-0.198	-0.011	0.055	-0.137	-0.007	-0.07
Holdemanella	-0.196	0.115	0.094	-0.125	-0.133	0.059	0.038	0.063	-0.016	-0.169	0.206	0.165	0.121	-0.167	-0.104	-0.2	-0.204	-0.001	0.021	-0.028	0.045	0.095	0.182	0.12	-0.098	0.063	-0.092
Amedibacterium	-0.181	-0.019	-0.01	-0.118	0.011	0.208	0.133	0.199	-0.124	-0.001	0.011	-0.047	0.025	-0.01	0.031	-0.022	-0.054	0.211	0.242	0.118	0.139	0.105	-0.076	0.021	-0.185	0.036	-0.148
Amedibacillus	-0.033	-0.16	-0.084	0.054	0.104	-0.056	-0.023	-0.059	-0.046	0.144	-0.191	-0.035	-0.067	-0.07	0.019	0.136	-0.021	-0.019	-0.021	0.173	0.151	0.095	0.214	0.192	0.161	-0.013	0.024
Catenibacterium	-0.276	0.169	0.034	-0.039	-0.049	0.015	0.226	-0.005	0.029	0.015	0.222	0.07	0.378	-0.089	-0.098	0.029	-0.244	-0.013	0.075	0.108	0.253	0.222	0.096	0.062	-0.108	-0.179	-0.142
Faecalibacillus	-0.033	-0.066	0.141	0.053	-0.073	0.135	-0.085	0.138	0.047	0.005	-0.101	0.106	-0.07	-0.106	-0.085	0.007	0.012	-0.076	-0.057	0.124	-0.15	-0.269	0.033	-0.026	-0.111	0.053	-0.073
Clostridium_g6	0.076	-0.011	-0.19	0.015	0.246	-0.016	0.015	-0.013	0.025	0.086	-0.116	-0.23	-0.021	0.018	0.049	0.099	0.12	0.042	0.08	0.014	0.038	0.051	-0.084	-0.005	0.104	0.023	0.146
Coprobacillus	0.145	-0.152	-0.26	-0.169	0.039	-0.089	-0.046	-0.067	-0.205	0.001	-0.105	-0.274	-0.097	0.071	-0.164	0.056	-0.009	0.264	0.219	0.076	0.053	-0.032	0.114	-0.02	0.09	0.095	0.085
Gemella	0.07	-0.18	0.038	0.054	0.046	-0.012	-0.114	-0.018	-0.035	-0.115	-0.117	0.085	-0.111	0.011	-0.168	-0.079	0.081	-0.038	0.099	-0.082	-0.001	-0.007	0.249	0.153	-0.024	0.12	0.161
Abiotrophia	0.11	-0.028	0.225	0.194	0.137	-0.067	0.094	-0.052	0.112	-0.026	0.041	0.171	0.009	-0.046	0.117	-0.014	-0.023	-0.049	-0.082	-0.071	-0.079	-0.083	0.119	0.146	-0.096	0.165	0.006
Ligilactobacillus	0.217	-0.278	-0.027	0.197	0.174	-0.244	-0.065	-0.202	-0.055	-0.007	-0.13	-0.042	0.068	0.053	-0.192	0.083	0.013	0.008	-0.035	-0.197	-0.087	0.035	-0.074	0.022	0.171	-0.057	0.014
Companilactobacillus	-0.03	-0.002	0.002	0.165	0.068	0.085	0.111	0.071	-0.122	0.12	0.096	0.04	0.127	-0.017	-0.042	0.131	-0.042	0.056	-0.058	-0.054	0.092	0.148	0.081	0.087	-0.068	-0.065	-0.207
Lacticaseibacillus	-0.091	-0.11	0.059	0.029	0.143	-0.088	0.172	-0.106	-0.133	-0.059	-0.102	0.039	-0.088	0.076	-0.035	-0.053	0.06	0.096	0.023	0.089	0.182	0.211	-0.045	0.073	0.023	-0.013	-0.074
Pediococcus	-0.12	-0.071	-0.042	-0.074	0.009	-0.065	0.035	-0.09	-0.052	0.167	0.069	-0.015	0.132	-0.018	-0.115	0.166	-0.016	0.001	-0.113	0.136	0.064	-0.001	0.266	0.204	0	0.113	-0.123
Limosilactobacillus	0.224	-0.136	-0.134	0.187	0.166	-0.259	0.198	-0.227	-0.223	0.112	-0.101	-0.088	0.118	0.01	-0.021	0.184	0.119	-0.111	-0.098	-0.118	-0.129	-0.074	0.074	0.055	0.143	0.05	0.207
Latilactobacillus	0.205	-0.069	-0.118	0.004	0.187	-0.273	0.161	-0.178	-0.046	0.019	-0.103	-0.128	-0.064	0.054	0.029	0.06	0.143	0.087	0.055	-0.043	-0.044	-0.036	0.177	0.255	0.269	0.125	0.027
Acidaminococcus	-0.033	-0.012	0.14	0.079	-0.006	-0.049	0.229	-0.014	-0.08	0.15	-0.107	0.095	0.083	-0.06	-0.093	0.137	0.031	0.044	-0.09	-0.01	-0.012	0.01	-0.033	0.059	-0.016	-0.027	-0.011
Phascolarctobacterium	-0.163	0.012	0.042	0.009	-0.045	0.188	-0.044	0.205	-0.017	-0.154	0.052	0.057	-0.066	0.013	-0.14	-0.126	-0.302	0.033	0.168	0.138	0.03	-0.014	0.016	-0.112	-0.053	-0.109	-0.087
Allisonella	-0.278	-0.02	0.062	-0.053	0.079	0.157	0.191	0.113	-0.188	0.04	0.015	0.218	0.095	0.009	-0.044	0.015	-0.15	-0.026	0.083	0.087	0.106	0.109	0.086	0.082	-0.236	-0.072	0.047
Dialister	0.117	0.069	0.132	0.021	-0.104	-0.18	0.02	-0.235	-0.026	0.14	-0.018	0.174	0.033	-0.164	-0.169	0.145	0.183	-0.027	-0.182	-0.114	-0.167	-0.122	0.077	0.164	0.069	-0.027	0.145
Veillonella	-0.095	-0.127	0.054	0.013	-0.086	0.007	0.007	-0.045	-0.142	-0.011	-0.104	0.099	-0.098	0.037	-0.075	-0.019	0.073	-0.081	-0.01	-0.125	0.075	0.25	-0.035	0.072	-0.213	-0.135	-0.13
Megasphaera	-0.092	0.098	-0.101	-0.129	0.076	0.092	0.23	0.081	-0.088	0.242	-0.02	-0.078	0.213	0.053	-0.089	0.237	-0.031	0.108	0.072	0.086	0.024	-0.031	-0.063	0.06	-0.04	-0.188	0.146
Megamonas	-0.368	0.039	0.072	-0.117	-0.085	0.131	-0.001	0.072	-0.046	0.063	0.06	0.095	0.073	0.033	-0.172	-0.006	-0.222	-0.076	-0.063	0.184	0.115	0.062	0.158	0.059	-0.049	-0.053	0.005
Selenomonas	-0.066	0.056	-0.035	0.065	0.102	0.151	0.116	0.118	-0.184	0.217	-0.202	-0.015	0.177	0.029	0.077	0.218	0.051	0.126	0.133	0.092	0.054	0.006	0.047	-0.016	-0.118	0.082	-0.189
Anaerofustis	0.16	-0.227	0.007	0.068	0.143	-0.213	-0.03	-0.137	0.075	-0.035	-0.035	0.045	0.006	0.049	-0.023	-0.059	0.055	0.012	0.06	0.028	-0.161	-0.219	0.043	0.006	-0.046	0.148	-0.015
Eubacterium	-0.025	0.051	-0.124	0.051	0.047	-0.165	-0.031	-0.198	-0.004	0.031	0.029	-0.095	0.071	0.199	0.103	0.027	0.05	0.058	0.181	0.131	0.063	-0.046	0.089	0.03	0.007	0.097	0.34
Borkfalkia	0.093	0.089	-0.039	0.231	0.143	-0.101	-0.122	-0.117	0.059	-0.048	0.01	-0.011	0.197	0.135	-0.028	-0.032	-0.063	-0.194	-0.238	-0.14	-0.102	-0.058	-0.049	0.026	0.058	0.028	0.207
Clostridium	0.261	-0.154	0.046	0.031	0.001	-0.104	-0.025	-0.074	-0.105	-0.15	-0.049	0.083	-0.235	0.072	-0.217	-0.115	0.022	-0.01	-0.083	-0.092	-0.161	-0.167	0.161	0.114	0.045	0.112	-0.051
Parvimonas	0.195	0.069	-0.165	-0.011	0.083	-0.079	0.033	-0.012	-0.015	-0.02	0.071	-0.065	0.007	0.015	-0.123	0.048	0.153	-0.087	-0.058	-0.168	-0.263	-0.28	0.106	0.136	0.098	0.17	0.096
Finegoldia	0.078	-0.046	-0.203	0.168	0.184	-0.024	-0.166	-0.019	0.163	-0.004	-0.122	-0.229	0.074	-0.078	0.11	-0.059	0.078	-0.096	-0.041	0.233	0.022	-0.018	0.011	-0.039	0.096	0.147	0.015
Peptoniphilus	0.172	-0.029	-0.068	-0.101	-0.072	-0.165	-0.065	-0.178	-0.028	-0.105	0.14	-0.056	-0.045	0.06	-0.123	-0.037	0.06	-0.037	-0.038	-0.203	-0.162	-0.113	0.147	0.079	0.056	0.163	0.084
Aedoeadaptatus	0.119	0.092	0.031	-0.058	-0.087	-0.147	-0.042	-0.124	0.101	-0.195	0.096	0.022	-0.121	0.009	-0.085	-0.144	0.039	-0.069	-0.052	-0.13	-0.108	-0.069	0.231	0.175	-0.137	0.066	0.014
Ezakiella	0.087	0.041	-0.078	-0.255	-0.114	0.029	0.051	0.004	-0.057	-0.016	0.099	-0.082	-0.061	-0.126	-0.096	0.037	0.012	0.13	0.099	-0.119	-0.077	-0.015	0.038	0.049	0.161	0.162	0.098
Guopingia	0.143	-0.104	-0.224	0.125	0.246	-0.137	-0.195	-0.105	0.01	0.192	-0.216	-0.185	0.01	-0.135	-0.023	0.21	0.054	-0.102	-0.132	0.245	0.013	-0.201	0.146	0.011	0.102	0.051	0.034
Beduinibacterium	0.169	-0.117	-0.151	0.108	0.092	-0.183	-0.158	-0.137	0.096	-0.109	-0.09	-0.094	-0.065	0.106	0.074	-0.115	0.151	0.038	-0.01	-0.079	-0.177	-0.208	0.004	-0.109	0.114	0.01	0.013
Luoshenia	0.133	0.115	-0.113	-0.007	0.207	-0.049	-0.083	-0.067	0.207	0.081	-0.044	-0.17	-0.054	-0.022	-0.06	0.088	0.033	0.235	0.221	0.169	0.15	-0.135	0.002	0.068	0.226	-0.008	0.137
Colidextribacter	0.155	-0.027	-0.096	0.052	0.06	-0.224	-0.035	-0.207	0.019	-0.105	-0.115	0.014	0.013	0.063	0.057	-0.11	0.227	-0.099	-0.136	0.032	0.035	-0.042	0.071	0.02	0.116	0.011	0.118
Evtepia	0.02	0.021	-0.024	0.036	0.001	-0.282	-0.073	-0.243	0.215	-0.03	0.086	0.016	0.031	0.022	-0.047	-0.071	0.038	-0.071	-0.103	0.091	-0.182	-0.338	0.046	0.046	0.116	0.062	0.083
Pseudoflavonifractor	0.217	-0.05	-0.167	0.069	0.025	-0.047	-0.164	-0.044	-0.029	-0.074	0.01	-0.145	-0.112	0.064	-0.026	-0.04	0.043	0.072	0.02	-0.091	0.029	0.048	-0.155	-0.221	0.175	-0.105	0.199
Sporobacter	0.212	-0.087	0.002	0.073	0.017	-0.13	-0.139	-0.135	0.041	-0.017	-0.184	0.004	0.057	-0.074	-0.115	0.014	0.07	0.011	-0.052	0.002	-0.037	-0.127	-0.05	-0.03	0.323	0.006	0.084
Marseillibacter	-0.001	-0.102	0.029	-0.118	-0.197	0.153	-0.037	0.15	0.2	0.052	-0.051	-0.068	0.079	-0.184	-0.115	0.06	-0.079	-0.044	0.056	-0.08	-0.103	-0.037	0.026	0.031	-0.037	-0.005	-0.116
Pusillibacter	0.075	0.012	-0.017	0.084	0.19	-0.009	0.016	-0.047	-0.185	0.196	-0.202	-0.055	0.092	0.121	-0.01	0.239	-0.012	0.122	0.059	0.108	0.032	-0.008	-0.128	-0.197	-0.016	-0.112	0.024
Oscillibacter	0.209	-0.002	-0.055	0.042	-0.025	-0.081	-0.157	-0.085	0.007	-0.029	-0.134	-0.024	-0.011	-0.08	-0.148	-0.01	0.078	0.029	-0.081	0.004	-0.077	-0.169	0.032	0.007	0.152	-0.021	0.115
Flintibacter	-0.02	-0.01	0.056	0.023	0.131	-0.011	0.241	-0.015	-0.057	0.079	-0.107	0.019	0.054	0.076	-0.05	0.067	-0.089	0.149	0.083	0.172	0.121	0.073	-0.177	-0.129	0.079	0.044	-0.15
Agathobaculum	-0.291	0.049	0.086	-0.168	-0.225	0.228	-0.11	0.201	0.221	-0.045	0.111	0.14	0.109	-0.105	-0.157	-0.081	-0.304	0.132	0.125	0.166	0.195	0.176	-0.065	-0.111	-0.258	-0.241	-0.15
Butyricicoccus	0.007	-0.048	0.216	0.072	-0.003	-0.049	0.106	-0.013	0.172	0.155	-0.098	0.137	0.009	-0.065	0.117	0.171	-0.102	0.077	0.043	-0.032	-0.01	0.025	-0.081	-0.066	-0.096	-0.075	-0.113
Monoglobus	0.08	-0.201	-0.087	0.017	0.127	-0.104	-0.038	-0.148	-0.028	-0.117	-0.17	-0.085	-0.022	-0.036	-0.027	-0.109	0.092	-0.15	-0.124	0.125	-0.053	-0.139	-0.077	-0.096	0.333	-0.04	0.126
Qingrenia	0.21	-0.101	-0.132	-0.024	-0.13	-0.014	-0.116	-0.052	-0.106	-0.186	-0.057	-0.067	-0.123	0.014	-0.074	-0.159	-0.026	0.06	0.028	-0.012	-0.131	-0.149	-0.121	-0.116	0.292	0.002	0.018
Bianquea	0.097	0.015	-0.141	0.043	0.16	-0.038	-0.011	-0.049	-0.024	0.096	-0.007	-0.15	0.154	0.125	-0.033	0.11	-0.015	0.078	0.086	0.116	0.116	-0.004	-0.001	0.046	0.201	0.046	0.077
Anaerotignum	-0.081	0.089	0.031	0.02	-0.063	-0.031	0.042	-0.041	0.232	-0.068	0.171	0.077	-0.042	-0.109	-0.05	-0.082	-0.175	0.123	0.074	0.194	0.006	-0.101	0.157	0.191	-0.135	0.095	-0.082
Aminicella	0.119	-0.036	0.085	0.07	-0.13	-0.12	0.036	-0.155	-0.14	0.029	0.012	0.128	0.02	0.17	-0.106	0.036	0.074	-0.031	-0.067	-0.261	-0.13	0.004	0.116	0.074	-0.022	0.105	0.085
Mogibacterium	0.026	-0.098	0.007	0.089	0.05	-0.178	0.088	-0.135	-0.023	-0.154	0.063	0.031	-0.152	0.057	-0.096	-0.149	0.06	-0.06	-0.06	-0.078	0.006	-0.047	0.219	0.217	0.075	0.164	0.142
Baileyella	0.143	-0.198	0.054	0.159	-0.028	-0.232	-0.016	-0.248	-0.022	0.12	-0.125	0.113	0.065	0.184	-0.106	0.152	0.056	0.045	-0.056	-0.086	-0.051	0.015	0.146	0.16	0.056	0.091	0.029
Emergencia	0.063	0.004	-0.194	0.256	0.232	-0.08	-0.151	-0.089	-0.011	0.246	0.062	-0.134	0.264	-0.034	-0.123	0.263	-0.068	0	-0.041	0.082	0.044	-0.088	-0.077	-0.15	0.049	-0.04	0.274
Lentihominibacter	0.061	-0.05	0.029	0.085	0.05	-0.197	-0.015	-0.214	0.032	-0.032	-0.002	0.022	0.078	-0.075	-0.143	0.004	0.101	0.046	-0.014	0.02	-0.027	-0.1	-0.007	0.097	0.205	0.151	0.177
Zhenpiania	-0.179	0.043	0.058	0.004	-0.008	-0.13	0.023	-0.139	-0.064	0.052	0.181	0.119	0.212	0.028	-0.139	0.044	-0.147	-0.025	0.009	0.08	-0.051	-0.102	0.015	0.044	-0.009	-0.005	0.075
Leuconostoc	-0.255	-0.059	0.063	0.002	0.07	0.026	0.093	0.014	-0.07	0.032	-0.053	0.079	-0.068	-0.075	-0.096	-0.015	-0.104	0.073	0.131	0.251	0.216	0.154	0.001	-0.017	-0.056	-0.038	-0.116
Weissella	-0.357	0.021	0.134	-0.013	0.022	0.149	0.165	0.108	-0.037	0.246	0.028	0.107	0.268	-0.247	-0.206	0.182	-0.198	-0.03	-0.045	0.236	0.31	0.302	0.069	0.042	-0.199	-0.069	-0.204
Streptococcus	-0.008	-0.08	0.021	0.059	0.138	-0.096	0.122	-0.153	-0.197	-0.101	-0.005	0.084	-0.061	0.051	-0.115	-0.099	0.179	-0.008	0.055	-0.069	-0.034	0.005	0.086	0.171	-0.037	0.141	0.12
Lactococcus	-0.065	0.075	0.034	0.019	-0.069	0.052	-0.085	0.041	-0.046	0.054	0.066	0.115	0.121	0.002	0.059	0.015	-0.024	0.043	-0.015	0.004	0.118	0.143	0.184	0.076	-0.231	0.007	-0.017
Neobitarella	0.15	-0.173	-0.143	0.05	0.137	-0.007	-0.221	-0.031	-0.154	0.05	-0.026	-0.141	0.005	0.049	-0.06	0.061	0.053	-0.099	-0.131	-0.105	-0.111	-0.088	-0.041	-0.069	0.159	0.118	0.137
Harryflintia	0.092	-0.01	0.037	0.096	-0.132	-0.141	0.005	-0.145	-0.016	0.005	-0.103	0.115	-0.023	0.112	0.277	-0.052	0.151	-0.157	-0.145	-0.113	0.165	0.172	-0.144	-0.143	0.184	-0.065	0.01
Negativibacillus	0.092	0.039	0.121	0.219	0.163	-0.123	0.098	-0.141	0.129	-0.047	0.136	0.099	0.1	-0.098	0.027	-0.031	0.032	-0.123	-0.106	0.031	-0.002	-0.099	0.003	0.069	-0.01	0.168	0.193
Fumia	0.299	-0.043	-0.011	0.087	0.12	-0.074	0.022	-0.013	-0.057	-0.097	-0.203	-0.026	-0.034	-0.094	-0.083	-0.045	0.065	-0.004	-0.016	0.038	-0.047	-0.079	0.001	0.007	0.117	-0.012	-0.064
Paludicola	0.106	0.116	-0.118	0.111	0.051	-0.003	-0.101	-0.045	0.049	-0.011	-0.017	-0.084	0.053	0.079	0.059	0.023	0.029	0.044	-0.048	-0.026	0.03	-0.02	-0.238	-0.242	0.24	-0.146	0.036
Anaerotruncus	0.127	0.059	-0.057	0.193	0.142	-0.224	-0.017	-0.144	0.093	0.052	0.138	-0.052	-0.084	0.002	0.083	0.047	0.141	0.077	0.024	-0.013	-0.053	-0.099	-0.02	0.091	0.064	0.185	0.122
Youxingia	-0.041	0.145	-0.13	0.026	0.12	-0.076	0.042	-0.044	-0.185	0.064	0.142	-0.062	0.192	0.07	0.106	0.09	0.005	-0.066	-0.087	0.082	0.246	0.22	0.131	0.075	-0.118	0.151	0.214
Ligaoa	0.113	-0.079	-0.027	0.064	0.185	-0.03	-0.004	-0.026	0.042	0.183	-0.215	0.013	0.128	0.026	-0.074	0.185	-0.077	0.027	-0.017	0.155	0.057	-0.043	-0.115	-0.066	-0.016	-0.17	0.018
Phocea	-0.009	-0.102	-0.099	0.117	0.043	-0.113	-0.082	-0.107	-0.044	0.021	-0.121	-0.04	-0.066	0.084	-0.106	0.028	-0.004	0.077	0.042	-0.052	-0.112	-0.086	-0.078	-0.111	-0.003	-0.197	0.022
Hydrogeniiclostidium	0.136	-0.19	-0.176	-0.115	0.045	-0.231	-0.142	-0.13	0.037	0.012	-0.068	-0.168	-0.041	0.001	-0.076	-0.019	0.043	0.062	0.073	-0.063	-0.225	-0.24	0.105	0.012	0.171	0.025	0.037
Pseudoruminococcus	0.054	-0.128	-0.105	-0.139	-0.168	-0.029	0.049	-0.023	-0.044	0.025	-0.107	-0.083	0.011	0.128	-0.085	0.044	0.065	0.066	0.12	-0.1	-0.058	0.01	-0.144	-0.134	0.262	-0.075	0.11
Unassigned	0.017	-0.072	-0.068	0.092	-0.038	-0.059	0.006	-0.074	0.079	0.104	-0.14	0.026	0.264	0.022	-0.259	0.129	-0.072	-0.013	0.081	-0.045	-0.155	-0.159	-0.062	-0.065	0.137	-0.094	0.067
Ruminococcus_g2	0.249	-0.05	0.031	0.066	-0.182	-0.142	-0.282	-0.175	-0.056	-0.174	-0.095	0.048	-0.132	0.274	-0.054	-0.164	0.108	-0.047	-0.041	-0.172	-0.231	-0.216	-0.034	-0.01	0.127	0.098	0.032
Anaeromassilibacillus	-0.133	0.141	0.097	0.076	0.134	-0.03	0.108	-0.049	-0.085	0.081	0.02	0.077	0.109	-0.018	-0.024	0.084	0.052	0.159	0.205	0.114	-0.003	-0.06	-0.207	-0.174	0.007	-0.082	0.184
Acutalibacter	0.11	0.05	-0.056	-0.105	-0.044	-0.315	-0.08	-0.224	0.074	-0.088	0.048	0.02	-0.103	-0.06	-0.047	-0.071	0.137	-0.1	-0.16	-0.094	-0.111	-0.142	0.05	0.044	0.055	0.091	0.031
Caproiciproducens	0.01	0.069	-0.124	0.129	0.288	0.023	-0.003	0.048	0.05	0.104	-0.145	-0.122	-0.144	-0.073	0.109	0.104	0.031	0.003	-0.101	0.135	0.02	-0.086	0.088	0.012	0.006	0.163	0.066
Solibaculum	0.162	-0.178	-0.083	0.076	0.097	-0.081	-0.139	0.099	0.072	0.009	0.016	-0.046	0.005	-0.005	-0.06	0.038	0.09	0.049	0.043	-0.186	-0.219	-0.201	0.053	0.02	0.033	0.147	0.137
Eubacterium_g8	0.215	0.046	-0.049	0.052	-0.073	-0.174	-0.054	-0.142	0.102	-0.121	-0.011	-0.051	0.021	-0.076	-0.07	-0.096	0.073	0.01	0.039	-0.141	-0.036	-0.03	0.027	0.007	0.076	0.047	0.007
Anaerobutyricum	-0.137	-0.062	0.04	-0.104	-0.189	0.103	-0.045	0.117	0.124	-0.093	-0.006	-0.002	0.023	-0.129	-0.161	-0.148	-0.094	0.037	-0.026	0.112	-0.087	-0.165	-0.056	-0.113	-0.053	-0.037	-0.249
Eubacterium_g17	0.271	-0.077	-0.03	0.035	0.019	-0.111	-0.139	-0.126	-0.053	-0.048	-0.192	-0.025	-0.073	0.118	-0.146	-0.011	-0.037	-0.038	-0.025	0.01	-0.122	-0.231	0.069	0.015	0.292	0.024	-0.041
Eubacterium_g24	-0.042	0.17	0.094	0.039	-0.082	0.144	-0.023	0.135	-0.006	-0.127	0.044	0.086	0.091	-0.213	-0.156	-0.107	-0.187	-0.083	0.011	-0.024	-0.033	-0.088	0.222	0.073	-0.062	-0.052	-0.024
Howardella	-0.072	0.048	0.029	-0.15	-0.108	0.018	-0.009	0.082	0.033	-0.128	0.012	0.001	0.148	-0.086	-0.029	-0.153	-0.09	0.208	0.265	-0.031	0.057	0.083	0.033	0.035	-0.031	-0.017	0.066
Lachnospira	0.086	-0.032	-0.031	0.01	-0.16	0.097	-0.175	0.101	0.072	0.007	-0.102	-0.022	0.087	0	-0.17	0.005	-0.166	-0.08	-0.043	0.001	-0.031	0.007	-0.159	-0.137	0.051	-0.259	-0.163
Anaerostipes	-0.058	0.053	0.003	0.042	-0.127	0.145	0.087	0.141	0.117	0.016	-0.085	-0.109	0.073	-0.032	0.054	0.015	-0.062	0.074	0.1	0.162	-0.03	-0.049	-0.113	-0.122	-0.119	-0.076	-0.242
Frisingicoccus	0.141	-0.112	-0.008	-0.001	-0.215	-0.136	-0.153	-0.116	0.162	0.008	-0.083	-0.009	0.034	-0.089	-0.17	0.004	0.096	0.03	-0.061	-0.061	-0.112	-0.119	0.173	0.119	0.101	-0.015	-0.024
Catenibacillus	0.169	-0.078	-0.145	0.169	0.158	-0.138	-0.097	-0.131	0.063	-0.049	-0.155	-0.157	-0.005	0.058	0.199	-0.136	0.172	-0.103	-0.058	0.171	-0.131	-0.172	-0.144	-0.143	0.205	0.155	0.01
Eubacterium_g4	0.095	0	0.051	-0.045	-0.015	0.006	-0.098	0.007	0.054	-0.236	-0.016	0.109	-0.107	0.174	-0.138	-0.217	-0.035	-0.008	-0.038	-0.007	-0.199	-0.228	0.079	0.046	0.05	-0.027	-0.014
Eisenbergiella	0	0.165	0.021	0.154	0.068	-0.011	0.048	-0.023	0.068	0.059	0.103	0.032	0.076	-0.031	0.326	0.04	0.058	-0.091	-0.053	0.038	0.133	0.118	-0.06	-0.005	0.045	-0.013	0.022
Acetitomaculum	-0.004	-0.223	0.033	-0.052	-0.075	-0.074	-0.131	-0.073	0.006	0.094	-0.091	0.021	0.059	0.022	-0.06	0.053	-0.105	-0.042	0.062	0.064	-0.078	-0.134	0.115	0.123	0.066	0.119	-0.11
Roseburia	-0.087	0.12	0.229	-0.066	-0.231	0.184	-0.018	0.144	0.031	-0.234	0.107	0.23	0.016	-0.021	-0.209	-0.242	-0.137	0.055	0.077	-0.105	-0.054	0.049	-0.062	-0.038	-0.098	-0.124	-0.12
Eubacterium_g21	-0.091	0.043	0.073	-0.207	-0.316	-0.006	-0.027	-0.027	0.161	-0.206	0.167	0.118	-0.065	0.096	-0.181	-0.196	-0.094	-0.055	-0.002	-0.028	-0.054	-0.047	0.202	0.076	-0.144	-0.071	0.027
Diplocloster	0.071	-0.105	0.065	-0.052	-0.004	-0.202	-0.228	-0.019	0.233	-0.225	0.042	0.073	-0.202	0.045	-0.085	-0.198	0.194	0.076	0.003	-0.088	-0.198	-0.213	0.052	0.06	-0.024	0.104	0.021
Coprococcus_g2	-0.099	0.095	-0.033	-0.082	-0.08	0.104	-0.024	0.071	0.079	-0.071	0.147	0.079	-0.04	-0.004	-0.071	-0.109	-0.023	0.016	0.146	0.002	0.182	0.178	0.147	0.227	-0.122	0.063	-0.084
Mediterraneibacter	-0.227	-0.037	-0.026	-0.077	0.017	0.129	-0.048	0.154	0.097	-0.023	-0.06	-0.014	0.067	-0.03	-0.095	-0.024	-0.133	0.201	0.206	0.1	0.058	-0.008	0.142	0.046	-0.179	0.064	0.009
Oribacterium	0.05	0.013	-0.002	0.117	0.031	-0.026	0.021	-0.029	0.06	-0.097	-0.047	0.025	-0.102	-0.016	-0.115	-0.084	0.166	-0.094	-0.015	-0.129	-0.068	-0.017	0.295	0.339	-0.113	0.164	0.093
Moryella	0.077	0.017	0.084	0.032	-0.091	-0.072	0.151	-0.076	-0.078	-0.202	-0.069	0.096	-0.106	0.089	-0.085	-0.163	0.048	-0.105	-0.057	-0.197	-0.053	0.047	0.173	0.194	-0.047	0.191	0.105
Porcincola	0.127	-0.043	-0.022	0.229	0.014	-0.094	0.14	-0.077	-0.026	0.194	-0.08	-0.059	0.169	-0.05	-0.074	0.194	-0.052	-0.077	-0.199	-0.126	0.011	0.088	0.07	0.013	0.011	-0.115	-0.088
Faecalibacterium	-0.122	-0.069	0.217	-0.166	-0.423	0.101	-0.086	0.083	-0.007	-0.106	0.018	0.283	-0.055	0.017	-0.189	-0.129	-0.127	0.013	-0.04	-0.119	-0.012	0.09	-0.082	-0.041	-0.008	-0.246	-0.187
Gemmiger	-0.055	0.009	0.107	-0.095	-0.22	-0.024	-0.128	-0.008	0.082	-0.179	0.035	0.207	-0.058	-0.041	-0.215	-0.207	-0.004	-0.045	-0.09	-0.025	-0.017	0.034	0.124	0.037	-0.148	-0.119	-0.057
Ruthenibacterium	0.036	0.041	-0.108	0.097	0.123	-0.22	0.018	-0.148	-0.09	0.098	-0.072	-0.139	0.02	0.036	0.031	0.145	0.014	0.111	0.099	0.056	-0.007	-0.019	-0.052	0.011	0.191	0.173	0.067
Anaerofilum	-0.001	-0.013	0.085	0.127	0.121	-0.101	-0.079	-0.121	0.027	0.047	-0.098	0.176	0.12	-0.034	-0.06	0.022	-0.11	-0.084	-0.133	0.087	-0.03	-0.101	-0.205	-0.114	0.081	-0.078	0.015
Clostridium_g35	0.156	-0.084	-0.226	-0.01	0.085	-0.034	-0.145	-0.055	-0.007	-0.016	-0.003	-0.218	0.02	0.145	0.027	0.015	-0.033	0.118	0.083	0.07	-0.013	-0.028	-0.208	-0.158	0.083	-0.019	0.04
Butyrivibrio_g1	0.222	-0.286	-0.168	0.114	-0.068	-0.054	0.046	-0.063	-0.012	-0.046	-0.145	-0.125	0.094	-0.021	-0.03	-0.024	0.051	-0.149	-0.121	-0.05	-0.105	-0.102	0.044	-0.074	0.101	-0.083	0.028
Blautia	-0.194	0.033	-0.082	0.136	0.086	-0.049	0.081	-0.084	0.036	0.032	0.152	-0.03	0.083	-0.039	0.02	0.011	-0.014	-0.024	0.044	0.231	0.046	-0.099	-0.051	-0.059	-0.153	-0.082	0.033

Supplementary Table 4. Spearman correlation matrix between IS gut microbiome taxa and clinical parameters

Spearman correlation coefficients between genus-level gut microbial taxa and clinical parameters in IS are displayed, with color shading indicating the strength and direction of the associations (red = positive, blue = negative). This table provides the numerical data for the Supplementary Figure 2.

	Age	WBC	Total Cholesterol	BUN	Creatinine	Hemoglobin	Triglycerides	Hematocrit	HDL	Fasting Blood Sugar	Platelet Count	LDL	Hemoglobin A1c	PT	hsCRP	Initial Glucose	D-Dimer	SBP	DBP	Height	Weight	BMI	Initial NIH Stroke Scale	Discharge NIH Stroke Scale	Pre-stroke MRS	Discharge MRS	Fazekas Scale
Methanobrevibacter	0.112	0.153	0.03	0.557	0.22	0.088	0.338	0.152	0.768	0.886	0.71	0.158	0.865	0.525	0.627	0.983	0.101	0.351	0.49	0.613	0.998	0.172	0.022	0.499	0.299	0.17	0.075
Methanosphaera	0.682	0.498	0.942	0.133	0.54	0.052	0.98	0.052	0.881	0.356	0.551	0.871	0.436	0.563	0.899	0.496	0.69	0.682	0.978	0.458	0.229	0.822	0.004	0.013	0.692	0.108	0.991
Nanosynbacter	0.743	0.398	0.213	0.563	0.039	0.972	0.854	0.861	0.977	0.106	0.767	0.192	0.333	0.557	0.398	0.121	0.686	0.236	0.175	0.018	0.404	0.739	0.376	0.205	0.954	0.443	0.291
Saccharimonas	0.705	0.172	0.277	0.262	0.097	0.757	0.1	0.389	0.115	0.438	0.491	0.195	0.107	0.783	0.678	0.408	0.796	0.107	0.131	0.089	0.086	0.093	0.781	0.638	0.041	0.602	0.073
Campylobacter	0.246	0.118	0.808	0.128	0.638	0.738	0.324	0.904	0.321	0.979	0.387	0.857	0.899	0.447	0.222	0.612	0.477	0.107	0.472	0.004	0.469	0.803	0.933	0.8	0.079	0.557	0.837
Fusobacterium	0.002	0.265	0.716	0.372	0.418	0.002	0.004	0.008	0.072	0.325	0.59	0.637	0.514	0.904	0.696	0.514	0.223	0.424	0.283	0.009	0.017	0.088	0.923	0.752	0.016	0.362	0.354
Leptotrichia	0.693	0.782	0.469	0.637	0.198	0.638	0.67	0.726	0.053	0.408	0.523	0.885	0.79	0.816	0.467	0.35	0.352	0.605	0.73	0.181	0.361	0.915	0.07	0.031	0.894	0.024	0.032
Cetobacterium	0.103	0.918	0.823	0.147	0.466	0.277	0.559	0.309	0.959	0.195	0.093	0.68	0.654	0.36	0.027	0.22	0.38	0.183	0.569	0.1	0.107	0.293	0.781	0.638	0.504	0.125	0.918
Akkermansia	0.033	0.884	0.512	0.548	0.385	0.006	0.974	0.038	0.931	0.003	0.883	0.368	0.001	0.197	0.628	0.006	0.079	0.477	0.579	0.112	0.029	0.01	0.491	0.297	0.018	0.184	0.006
Prevotella	0.035	0.263	0.528	0.066	0.62	0.048	0.374	0.055	0.619	0.064	0.272	0.358	0.568	0.026	0.374	0.073	0.016	0.506	0.784	0.888	0.172	0.11	0.295	0.624	0.332	0.019	0.78
Capnocytophaga	0.959	0.644	0.149	0.823	0.175	0.758	0.918	0.489	0.067	0.284	0.286	0.387	0.584	0.782	0.555	0.319	0.395	0.955	0.386	0.045	0.044	0.238	0.696	0.823	0.342	0.935	0.175
Lancefieldella	0.463	0.694	0.362	0.154	0.394	0.741	0.742	0.375	0.532	0.952	0.483	0.281	0.837	0.889	0.943	0.908	0.897	0.246	0.439	0.06	0.71	0.422	0.463	0.062	0.341	0.041	0.844
Olsenella	0.705	0.142	0.124	0.655	0.499	0.086	0.705	0.086	0.513	0.783	0.1	0.156	0.428	0.269	0.006	0.877	0.208	0.086	0.085	0.438	0.161	0.108	0.083	0.102	0.504	0.125	0.073
Collinsella	0.103	0.414	0.453	0.009	0.939	0.894	0.045	0.977	0.842	0.734	0.347	0.526	0.489	0.262	0.244	0.716	0.146	0.031	0.106	0.129	0.296	0.966	0.226	0.041	0.894	0.632	0.501
Senegalimassilia	0.455	0.03	0.529	0.88	0.441	0.11	0.99	0.492	0.803	0.506	0.408	0.909	0.551	0.408	0.895	0.703	0.301	0.038	0.071	0.479	0.5	0.564	0.067	0.313	0.16	0.667	0.062
Ellagibacter	0.821	0.287	0.088	0.12	0.859	0.529	0.936	0.453	0.026	0.674	0.209	0.645	0.502	0.296	0.127	[illegible]	0.511	0.259	0.188	0.684	0.511	0.269	0.831	0.485	0.107	0.131	0.083
Gordonibacter	0.187	0.461	0.962	0.772	0.138	0.413	0.57	0.377	0.241	0.193	0.966	0.783	0.22	0.897	0.428	0.289	0.202	0.779	0.282	0.015	0.439	0.79	0.338	0.868	0.896	0.885	0.281
Eggerthella	0.18	0.306	0.185	0.223	0.295	0.018	0.326	0.035	0.225	0.032	0.876	0.402	0.509	0.001	0.001	0.053	0.04	0.156	0.294	0.151	0.194	0.432	0.521	0.026	0.334	0.809	0.061
Lawsonella	0.418	0.072	0.508	0.431	0.311	0.212	0.997	0.295	0.822	0.949	0.061	0.302	0.721	0.534	0.555	0.767	0.207	0.465	0.65	0.805	0.161	0.03	0.231	0.03	0.425	0.541	0.883
Corynebacterium	0.493	0.046	0.6	0.822	0.326	0.847	0.058	0.608	0.237	0.786	0.843	0.481	0.369	0.066	0.296	0.699	0.742	0.105	0.121	0.068	0.071	0.369	0.32	0.908	0.894	0.362	0.815
Actinomyces	0.392	0.863	0.619	0.169	0.213	0.828	0.511	0.783	0.339	0.7	0.578	0.931	0.988	0.05	0.719	0.654	0.889	0.968	0.609	0.404	0.479	0.829	0.129	0.094	0.616	0.237	0.833
Schaalia	0.607	0.175	0.892	0.109	0.388	0.445	0.608	0.285	0.354	0.678	0.665	0.443	0.63	0.031	0.067	0.715	0.918	0.82	0.565	0.346	0.712	0.706	0.107	0.106	0.964	0.409	0.121
Bifidobacterium	0.14	0.498	0.236	0.228	0.907	0.273	0.004	0.223	0.654	0.061	0.92	0.647	0.572	0.852	0.683	0.026	0.756	0.112	0.609	0.946	0.541	0.251	0.054	0.872	0.984	0.354	0.114
Alloscardovia	0.039	0.697	0.457	0.375	0.576	0.009	0.986	0.005	0.142	0.613	0.598	0.915	0.477	0.507	0.008	0.636	0.051	0.292	0.136	0.004	0.003	0.012	0.516	0.772	0.263	0.452	0.031
Pyramidobacter	0.046	0.262	0.311	0.684	0.519	0.022	0.692	0.053	0.146	0.307	0.978	0.664	0.176	0.548	0.68	0.451	0.116	0.605	0.104	0.08	0.285	0.251	0.181	0.02	0.415	0.322	0.365
Parasutterella	0.04	0.56	0.508	0.272	0.19	0.878	0.628	0.978	0.443	0.695	0.035	0.747	0.812	0.68	0.553	0.58	0.895	0.115	0.217	0.048	0.311	0.724	0.704	0.491	0.098	0.442	0.973
Sutterella	0.049	0.446	0.261	0.417	0.818	0.47	0.533	0.976	0.162	0.657	0.974	0.207	0.047	0.184	0.6	0.623	0.173	0.343	0.917	0.291	0.901	0.598	0.348	0.425	0.465	0.108	0.59
Mesosutterella	0.527	0.602	0.385	0.848	0.024	0.809	0.369	0.816	0.797	0.038	0.725	0.554	0.13	0.089	0.527	0.056	0.336	0.199	0.305	0.097	0.483	0.655	0.921	0.695	0.798	0.359	0.5
Massilia	0.904	0.77	0.877	0.38	0.217	0.107	0.37	0.1	0.309	0.973	0.248	0.837	0.904	0.863	0.007	0.77	0.172	0.183	0.14	0.438	0.379	0.68	0.423	0.392	0.504	0.256	0.073
Oxalobacter	0.352	0.437	0.962	0.801	0.565	0.878	0.061	0.675	0.772	0.043	0.111	0.783	0.381	0.722	0.555	0.016	0.644	0.292	0.256	0.977	0.175	0.064	0.885	0.042	0.534	0.113	0.883
Neisseria	0.103	0.232	0.045	0.965	0.202	0.879	0.221	0.957	0.679	0.442	0.512	0.143	0.788	0.924	0.398	0.332	0.007	0.969	0.742	0.803	0.418	0.203	0.207	0.184	0.232	0.853	0.276
Haemophilus	0.644	0.033	0.821	0.663	0.864	0.519	0.636	0.251	0.224	0.09	0.943	0.108	0.252	0.551	0.387	0.064	0.568	0.925	0.908	0.742	0.733	0.389	0.973	0.706	0.511	0.692	0.78
Bilophila	0.242	0.166	0.914	0.018	0.186	0.246	0.38	0.146	0.551	0.947	0.845	0.551	0.927	0.807	0.214	0.982	0.117	0.008	0.139	0.864	0.608	0.226	0.125	0.764	0.539	0.907	0.259
Desulfovibrio	0.011	0.806	0.052	0.782	0.524	0.009	0.583	0.015	0.532	0.067	0.636	0.14	0.947	0.941	0.882	0.371	0.138	0.256	0.061	0.328	0.098	0.024	0.006	0.265	0.154	0.189	6.94E-05
Paracoccus	0.77	0.986	0.986	0.1	0.499	0.399	0.27	0.48	0.227	0.234	0.343	0.692	0.207	0.863	0.678	0.195	0.68	0.581	0.569	0.594	0.361	0.142	0.423	0.392	0.504	0.517	0.038
Novosphingobium	0.904	0.77	0.877	0.38	0.217	0.107	0.37	0.1	0.309	0.973	0.248	0.837	0.904	0.863	0.007	0.77	0.172	0.183	0.14	0.438	0.379	0.68	0.423	0.392	0.504	0.256	0.073
Romboutsia	0.138	0.208	0.757	0.624	0.852	0.5	0.593	0.689	0.034	0.004	0.479	0.548	0.016	0.088	0.004	0.006	0.859	0.777	0.92	0.179	0.208	0.447	0.757	0.785	0.069	0.703	0.956
Bulleidia	0.015	0.356	0.036	0.072	0.031	0.848	0.455	0.706	0.53	0.198	0.053	0.056	0.48	0.919	0.467	0.123	0.921	0.993	0.891	0.205	0.828	0.662	0.56	0.112	0.242	0.421	0.386
Solobacterium	0.148	0.134	0.333	0.727	0.929	0.102	0.001	0.31	0.069	0.493	0.705	0.69	0.065	0.495	0.222	0.457	0.373	0.168	0.378	0.922	0.166	0.139	0.065	0.641	0.766	0.935	0.716
Holdemania	0.26	0.368	0.051	0.534	0.904	0.157	0.908	0.138	0.584	0.584	0.47	0.024	0.966	0.074	0.187	0.927	0.329	0.768	0.116	0.179	0.629	0.357	0.89	0.916	0.378	0.361	0.045
Dielma	0.915	0.808	0.845	0.709	0.235	0.98	0.446	0.754	0.873	0.295	0.306	0.633	0.146	0.96	0.467	0.594	0.181	0.388	0.558	0.042	0.159	0.364	0.919	0.668	0.242	0.794	0.237
Clostridium_g36	0.181	0.31	0.056	0.208	0.125	0.054	0.534	0.201	0.74	0.375	0.431	0.03	0.51	0.992	0.762	0.215	0.063	0.51	0.904	0.331	0.654	0.857	0.628	0.885	0.859	0.04	0.198
Merdibacter	0.879	0.71	0.84	0.193	0.585	0.903	0.055	0.712	0.575	0.861	0.902	0.913	0.465	0.275	0.924	0.834	0.763	0.856	0.925	0.334	0.306	0.122	0.026	0.035	0.474	0.384	0.56
Faecalicoccus	0.819	0.054	0.98	0.091	0.043	0.963	0.821	0.904	0.127	0.054	0.354	0.479	0.495	0.415	0.342	0.047	0.573	0.395	0.967	0.073	0.088	0.651	0.21	0.116	0.768	0.345	0.535
Absicoccus	0.175	0.851	0.921	0.166	0.97	0.111	0.556	0.106	0.843	0.86	0.272	0.942	0.744	0.21	0.398	0.793	0.848	0.277	0.026	0.131	0.756	0.43	0.169	0.326	0.643	0.688	0.295
Faecalitalea	0.705	0.382	0.112	0.604	0.799	0.586	0.06	0.58	0.764	0.479	0.641	0.054	0.374	0.824	0.398	0.334	0.175	0.057	0.024	0.699	0.193	0.049	0.913	0.589	0.174	0.944	0.489
Holdemanella	0.05	0.253	0.354	0.214	0.186	0.562	0.704	0.532	0.877	0.093	0.04	0.101	0.229	0.096	0.303	0.046	0.042	0.991	0.84	0.779	0.659	0.349	0.07	0.235	0.334	0.535	0.361
Amedibacterium	0.071	0.852	0.921	0.242	0.916	0.037	0.186	0.047	0.217	0.989	0.914	0.64	0.802	0.921	0.762	0.827	0.591	0.035	0.015	0.242	0.168	0.3	0.451	0.836	0.066	0.723	0.142
Amedibacillus	0.743	0.112	0.408	0.593	0.303	0.581	0.818	0.558	0.649	0.153	0.057	0.73	0.507	0.488	0.85	0.176	0.834	0.848	0.835	0.085	0.134	0.349	0.032	0.056	0.11	0.9	0.813
Catenibacterium	0.005	0.093	0.737	0.329	0.63	0.886	0.024	0.958	0.773	0.851	0.027	0.486	0	0.378	0.33	0.773	0.015	0.894	0.46	0.285	0.011	0.027	0.343	0.54	0.287	0.074	0.158
Faecalibacillus	0.747	0.516	0.163	0.603	0.469	0.179	0.4	0.171	0.644	0.959	0.317	0.293	0.489	0.292	0.403	0.945	0.902	0.45	0.574	0.22	0.136	0.007	0.747	0.796	0.273	0.6	0.47
Clostridium_g6	0.451	0.912	0.059	0.881	0.013	0.878	0.881	0.896	0.807	0.397	0.249	0.021	0.835	0.859	0.629	0.325	0.236	0.678	0.43	0.893	0.707	0.617	0.407	0.961	0.303	0.821	0.147
Coprobacillus	0.149	0.132	0.009	0.093	0.702	0.378	0.653	0.508	0.041	0.994	0.3	0.006	0.336	0.482	0.103	0.582	0.925	0.008	0.029	0.455	0.602	0.751	0.26	0.842	0.373	0.348	0.399
Gemella	0.491	0.073	0.705	0.597	0.65	0.907	0.26	0.858	0.726	0.253	0.245	0.399	0.27	0.912	0.095	0.432	0.423	0.708	0.325	0.42	0.994	0.946	0.013	0.128	0.815	0.235	0.109
Abiotrophia	0.275	0.783	0.024	0.053	0.175	0.508	0.352	0.606	0.269	0.795	0.688	0.088	0.929	0.646	0.245	0.889	0.823	0.628	0.417	0.48	0.435	0.412	0.237	0.147	0.342	0.102	0.949
Ligilactobacillus	0.03	0.005	0.787	0.049	0.083	0.015	0.521	0.044	0.588	0.943	0.196	0.675	0.502	0.599	0.056	0.411	0.898	0.94	0.73	0.05	0.387	0.728	0.462	0.83	0.089	0.573	0.894
Companilactobacillus	0.77	0.986	0.986	0.1	0.499	0.399	0.27	0.48	0.227	0.234	0.343	0.692	0.207	0.863	0.678	0.195	0.68	0.581	0.569	0.594	0.361	0.142	0.423	0.392	0.504	0.517	0.038
Lacticaseibacillus	0.368	0.275	0.562	0.777	0.156	0.383	0.086	0.294	0.186	0.561	0.311	0.7	0.385	0.451	0.728	0.601	0.55	0.342	0.823	0.38	0.07	0.035	0.654	0.471	0.824	0.897	0.466
Pediococcus	0.233	0.485	0.679	0.466	0.928	0.52	0.73	0.375	0.606	0.097	0.494	0.883	0.19	0.86	0.256	0.099	0.873	0.988	0.263	0.177	0.525	0.991	0.008	0.041	0.998	0.264	0.222
Limosilactobacillus	0.025	0.179	0.184	0.062	0.098	0.009	0.049	0.023	0.026	0.267	0.319	0.384	0.241	0.924	0.832	0.067	0.24	0.272	0.334	0.242	0.201	0.467	0.462	0.589	0.155	0.619	0.039
Latilactobacillus	0.04	0.496	0.241	0.97	0.062	0.006	0.11	0.077	0.648	0.848	0.307	0.203	0.529	0.596	0.772	0.551	0.155	0.388	0.59	0.67	0.665	0.72	0.079	0.01	0.007	0.216	0.787
Acidaminococcus	0.746	0.908	0.164	0.434	0.955	0.63	0.022	0.892	0.429	0.136	0.29	0.349	0.413	0.551	0.359	0.174	0.756	0.664	0.373	0.922	0.902	0.921	0.746	0.56	0.873	0.786	0.917
Phascolarctobacterium	0.105	0.907	0.678	0.927	0.659	0.061	0.663	0.041	0.865	0.126	0.605	0.575	0.512	0.899	0.164	0.212	0.002	0.745	0.095	0.17	0.765	0.893	0.877	0.266	0.601	0.281	0.389
Allisonella	0.005	0.846	0.538	0.599	0.437	0.118	0.057	0.263	0.061	0.693	0.882	0.029	0.348	0.928	0.661	0.882	0.137	0.795	0.409	0.389	0.292	0.28	0.397	0.415	0.018	0.476	0.639
Dialister	0.245	0.493	0.189	0.839	0.303	0.073	0.84	0.019	0.8	0.166	0.855	0.083	0.743	0.103	0.092	0.151	0.068	0.79	0.071	0.257	0.096	0.225	0.445	0.102	0.498	0.792	0.151
Veillonella	0.345	0.21	0.594	0.9	0.396	0.946	0.947	0.66	0.159	0.915	0.304	0.326	0.33	0.717	0.458	0.85	0.468	0.423	0.923	0.214	0.46	0.012	0.733	0.474	0.033	0.181	0.199
Megasphaera	0.364	0.332	0.317	0.202	0.45	0.361	0.021	0.423	0.385	0.015	0.84	0.443	0.034	0.602	0.376	0.017	0.763	0.284	0.479	0.396	0.81	0.761	0.536	0.556	0.696	0.061	0.146
Megamonas	0	0.702	0.475	0.245	0.4	0.194	0.992	0.479	0.651	0.536	0.556	0.348	0.469	0.745	0.088	0.955	0.026	0.454	0.532	0.068	0.255	0.543	0.118	0.56	0.627	0.601	0.958
Selenomonas	0.517	0.582	0.729	0.519	0.311	0.133	0.25	0.241	0.066	0.03	0.044	0.88	0.078	0.776	0.447	0.029	0.611	0.212	0.189	0.365	0.596	0.95	0.645	0.874	0.242	0.42	0.059
Anaerofustis	0.113	0.023	0.941	0.501	0.156	0.034	0.766	0.173	0.459	0.729	0.73	0.656	0.953	0.626	0.822	0.559	0.59	0.907	0.555	0.779	0.11	0.028	0.67	0.95	0.648	0.141	0.879
Eubacterium	0.802	0.613	0.219	0.613	0.64	0.101	0.763	0.049	0.971	0.763	0.774	0.345	0.48	0.047	0.308	0.787	0.62	0.565	0.071	0.193	0.533	0.647	0.38	0.767	0.945	0.337	0.001
Borkfalkia	0.359	0.377	0.7	0.021	0.155	0.318	0.227	0.245	0.561	0.632	0.92	0.916	0.049	0.18	0.782	0.753	0.533	0.053	0.017	0.164	0.31	0.568	0.627	0.796	0.566	0.779	0.039
Clostridium	0.009	0.125	0.648	0.763	0.989	0.301	0.809	0.463	0.297	0.137	0.626	0.414	0.019	0.474	0.03	0.254	0.827	0.919	0.411	0.362	0.108	0.097	0.109	0.259	0.66	0.266	0.613
Parvimonas	0.052	0.495	0.1	0.91	0.409	0.434	0.746	0.907	0.884	0.841	0.485	0.524	0.948	0.88	0.222	0.632	0.129	0.391	0.566	0.095	0.008	0.005	0.295	0.178	0.33	0.09	0.344
Finegoldia	0.443	0.647	0.043	0.096	0.067	0.81	0.099	0.848	0.105	0.966	0.228	0.022	0.465	0.438	0.277	0.558	0.443	0.341	0.688	0.02	0.829	0.862	0.914	0.703	0.344	0.145	0.883
Peptoniphilus	0.087	0.771	0.503	0.318	0.477	0.101	0.52	0.077	0.78	0.297	0.166	0.579	0.655	0.554	0.222	0.711	0.552	0.713	0.704	0.043	0.107	0.265	0.143	0.436	0.581	0.104	0.404
Aedoeadaptatus	0.236	0.362	0.759	0.564	0.391	0.146	0.675	0.22	0.318	0.052	0.344	0.829	0.232	0.929	0.398	0.153	0.697	0.492	0.61	0.198	0.285	0.494	0.021	0.081	0.174	0.512	0.894
Ezakiella	0.389	0.686	0.44	0.01	0.26	0.773	0.616	0.966	0.575	0.873	0.328	0.418	0.546	0.213	0.342	0.716	0.903	0.196	0.326	0.239	0.445	0.88	0.705	0.63	0.11	0.108	0.331
Guopingia	0.155	0.305	0.025	0.215	0.014	0.173	0.052	0.297	0.918	0.056	0.031	0.066	0.924	0.179	0.817	0.036	0.59	0.315	0.192	0.014	0.898	0.045	0.147	0.917	0.31	0.614	0.74
Beduinibacterium	0.093	0.247	0.133	0.284	0.365	0.069	0.116	0.175	0.342	0.28	0.375	0.352	0.522	0.293	0.465	0.253	0.133	0.704	0.92	0.435	0.079	0.038	0.972	0.282	0.257	0.925	0.896
Luoshenia	0.187	0.256	0.263	0.945	0.038	0.625	0.411	0.505	0.039	0.425	0.661	0.091	0.595	0.83	0.555	0.383	0.747	0.018	0.027	0.093	0.138	0.182	0.987	0.503	0.024	0.935	0.175
Colidextribacter	0.122	0.787	0.343	0.61	0.554	0.025	0.73	0.039	0.851	0.297	0.255	0.888	0.895	0.536	0.576	0.276	0.023	0.329	0.177	0.751	0.728	0.68	0.48	0.845	0.251	0.914	0.24
Evtepia	0.84	0.836	0.813	0.72	0.992	0.005	0.47	0.015	0.031	0.767	0.397	0.877	0.758	0.831	0.642	0.483	0.706	0.482	0.307	0.367	0.07	0.001	0.65	0.651	0.249	0.543	0.414
Pseudoflavonifractor	0.03	0.619	0.098	0.493	0.803	0.644	0.102	0.661	0.772	0.464	0.923	0.15	0.268	0.528	0.795	0.694	0.673	0.477	0.844	0.37	0.777	0.633	0.124	0.027	0.081	0.298	0.047
Sporobacter	0.034	0.388	0.985	0.472	0.869	0.198	0.168	0.18	0.683	0.868	0.067	0.97	0.577	0.467	0.256	0.89	0.491	0.916	0.608	0.986	0.717	0.207	0.62	0.765	0.001	0.95	0.407
Marseillibacter	0.993	0.315	0.775	0.24	0.049	0.128	0.716	0.136	0.046	0.608	0.617	0.504	0.434	0.067	0.256	0.556	0.435	0.666	0.579	0.428	0.308	0.715	0.794	0.761	0.712	0.96	0.249
Pusillibacter	0.456	0.904	0.87	0.404	0.058	0.932	0.877	0.645	0.065	0.051	0.044	0.584	0.363	0.231	0.92	0.017	0.902	0.228	0.563	0.284	0.751	0.936	0.206	0.05	0.875	0.267	0.812
Oscillibacter	0.037	0.982	0.59	0.678	0.803	0.421	0.118	0.4	0.947	0.771	0.185	0.812	0.916	0.429	0.142	0.924	0.44	0.773	0.423	0.969	0.447	0.092	0.753	0.948	0.131	0.836	0.253
Flintibacter	0.846	0.918	0.582	0.824	0.193	0.917	0.016	0.88	0.573	0.433	0.291	0.85	0.593	0.451	0.62	0.507	0.378	0.138	0.409	0.087	0.232	0.469	0.077	0.2	0.435	0.663	0.137
Agathobaculum	0.003	0.631	0.397	0.095	0.024	0.022	0.275	0.045	0.027	0.657	0.271	0.166	0.279	0.297	0.119	0.425	0.002	0.19	0.214	0.098	0.052	0.08	0.521	0.273	0.01	0.016	0.137
Butyricicoccus	0.942	0.639	0.031	0.48	0.978	0.631	0.293	0.898	0.088	0.124	0.333	0.174	0.929	0.522	0.245	0.089	0.311	0.445	0.668	0.751	0.919	0.806	0.421	0.511	0.342	0.46	0.263
Monoglobus	0.43	0.044	0.388	0.868	0.207	0.302	0.706	0.143	0.783	0.247	0.09	0.403	0.83	0.721	0.788	0.279	0.364	0.137	0.221	0.216	0.597	0.166	0.444	0.342	0.001	0.691	0.212
Qingrenia	0.036	0.315	0.19	0.809	0.199	0.894	0.252	0.609	0.293	0.064	0.577	0.507	0.222	0.893	0.467	0.114	0.801	0.554	0.78	0.904	0.195	0.138	0.23	0.249	0.003	0.986	0.856
Bianquea	0.335	0.88	0.163	0.67	0.111	0.71	0.912	0.631	0.816	0.342	0.946	0.135	0.126	0.215	0.744	0.277	0.88	0.438	0.397	0.251	0.25	0.968	0.993	0.649	0.045	0.646	0.449
Anaerotignum	0.426	0.376	0.76	0.842	0.536	0.763	0.676	0.683	0.02	0.503	0.089	0.449	0.676	0.282	0.624	0.415	0.081	0.224	0.462	0.053	0.952	0.317	0.119	0.057	0.182	0.349	0.419
Aminicella	0.239	0.719	0.401	0.487	0.196	0.236	0.725	0.124	0.164	0.777	0.905	0.205	0.845	0.09	0.296	0.724	0.464	0.756	0.506	0.009	0.198	0.968	0.251	0.462	0.825	0.299	0.4
Mogibacterium	0.795	0.334	0.947	0.377	0.618	0.077	0.384	0.18	0.822	0.127	0.535	0.761	0.13	0.573	0.342	0.138	0.555	0.551	0.555	0.44	0.956	0.645	0.028	0.03	0.46	0.103	0.16
Baileyella	0.156	0.049	0.594	0.115	0.781	0.02	0.873	0.013	0.829	0.235	0.215	0.264	0.521	0.066	0.296	0.13	0.583	0.656	0.582	0.396	0.618	0.881	0.147	0.112	0.578	0.365	0.777
Emergencia	0.536	0.965	0.053	0.01	0.02	0.431	0.134	0.379	0.913	0.013	0.54	0.183	0.008	0.735	0.222	0.008	0.503	0.998	0.689	0.418	0.666	0.383	0.444	0.136	0.628	0.696	0.006
Lentihominibacter	0.546	0.623	0.772	0.4	0.621	0.05	0.879	0.033	0.752	0.755	0.981	0.83	0.439	0.46	0.156	0.969	0.318	0.651	0.888	0.843	0.788	0.323	0.948	0.339	0.041	0.133	0.079
Zhenpiania	0.075	0.668	0.569	0.971	0.933	0.199	0.817	0.169	0.526	0.607	0.072	0.24	0.034	0.785	0.168	0.664	0.146	0.804	0.933	0.426	0.612	0.312	0.881	0.662	0.926	0.958	0.456
Leuconostoc	0.01	0.56	0.535	0.983	0.487	0.797	0.356	0.89	0.488	0.753	0.598	0.437	0.503	0.461	0.344	0.879	0.303	0.47	0.194	0.012	0.031	0.127	0.989	0.868	0.579	0.705	0.251
Weissella	0	0.837	0.184	0.901	0.829	0.14	0.101	0.286	0.715	0.014	0.782	0.287	0.007	0.013	0.04	0.07	0.048	0.771	0.658	0.018	0.002	0.002	0.496	0.678	0.047	0.495	0.042
Streptococcus	0.936	0.429	0.838	0.557	0.171	0.341	0.227	0.128	0.05	0.32	0.961	0.407	0.545	0.617	0.256	0.326	0.075	0.938	0.588	0.496	0.735	0.958	0.395	0.089	0.716	0.162	0.236
Lactococcus	0.519	0.459	0.737	0.851	0.492	0.609	0.402	0.687	0.646	0.596	0.516	0.253	0.229	0.982	0.562	0.88	0.812	0.673	0.881	0.97	0.243	0.155	0.067	0.452	0.021	0.942	0.868
Neobitarella	0.137	0.085	0.155	0.62	0.175	0.942	0.027	0.759	0.126	0.623	0.8	0.161	0.962	0.629	0.555	0.548	0.598	0.327	0.193	0.298	0.27	0.384	0.687	0.494	0.114	0.24	0.175
Harryflintia	0.361	0.918	0.718	0.343	0.192	0.162	0.959	0.151	0.877	0.959	0.309	0.255	0.823	0.269	0.005	0.606	0.133	0.119	0.15	0.262	0.1	0.086	0.152	0.156	0.067	0.517	0.918
Negativibacillus	0.365	0.699	0.229	0.029	0.105	0.223	0.331	0.161	0.201	0.642	0.178	0.327	0.323	0.33	0.788	0.756	0.755	0.223	0.292	0.763	0.986	0.325	0.979	0.497	0.919	0.095	0.054
Fumia	0.003	0.668	0.911	0.39	0.233	0.463	0.825	0.898	0.574	0.338	0.043	0.797	0.74	0.353	0.414	0.659	0.52	0.97	0.877	0.706	0.642	0.437	0.994	0.946	0.244	0.907	0.528
Paludicola	0.294	0.251	0.242	0.273	0.614	0.978	0.318	0.659	0.626	0.914	0.863	0.404	0.598	0.433	0.561	0.82	0.776	0.662	0.638	0.801	0.769	0.845	0.017	0.015	0.016	0.147	0.724
Anaerotruncus	0.208	0.557	0.572	0.055	0.158	0.025	0.864	0.153	0.358	0.604	0.171	0.608	0.407	0.984	0.409	0.64	0.162	0.449	0.812	0.9	0.599	0.327	0.847	0.37	0.528	0.065	0.225
Youxingia	0.686	0.15	0.196	0.795	0.235	0.451	0.68	0.665	0.066	0.53	0.158	0.543	0.056	0.488	0.295	0.376	0.964	0.513	0.388	0.417	0.014	0.028	0.193	0.457	0.242	0.135	0.032
Ligaoa	0.262	0.435	0.786	0.525	0.065	0.767	0.969	0.795	0.676	0.069	0.031	0.894	0.205	0.799	0.467	0.066	0.445	0.79	0.864	0.123	0.573	0.673	0.255	0.516	0.877	0.091	0.856
Phocea	0.929	0.314	0.327	0.247	0.669	0.263	0.416	0.289	0.665	0.838	0.231	0.695	0.517	0.409	0.296	0.785	0.972	0.447	0.678	0.606	0.269	0.394	0.442	0.272	0.975	0.049	0.826
Hydrogeniiclostidium	0.177	0.059	0.08	0.253	0.656	0.021	0.158	0.199	0.713	0.909	0.504	0.095	0.685	0.989	0.45	0.852	0.671	0.542	0.472	0.531	0.025	0.016	0.297	0.902	0.09	0.807	0.718
Pseudoruminococcus	0.596	0.206	0.299	0.168	0.094	0.775	0.626	0.821	0.661	0.806	0.287	0.41	0.915	0.204	0.398	0.664	0.523	0.512	0.234	0.324	0.567	0.92	0.152	0.185	0.008	0.46	0.276
Unassigned	0.868	0.479	0.5	0.365	0.706	0.559	0.952	0.463	0.436	0.302	0.166	0.797	0.008	0.832	0.009	0.202	0.477	0.9	0.422	0.656	0.123	0.114	0.542	0.521	0.174	0.354	0.505
Ruminococcus_g2	0.012	0.619	0.758	0.514	0.071	0.159	0.005	0.081	0.581	0.083	0.346	0.632	0.19	0.006	0.591	0.102	0.285	0.64	0.682	0.087	0.021	0.031	0.733	0.918	0.209	0.332	0.754
Anaeromassilibacillus	0.188	0.162	0.339	0.454	0.184	0.765	0.284	0.632	0.403	0.421	0.84	0.447	0.281	0.858	0.812	0.404	0.61	0.114	0.041	0.261	0.973	0.551	0.039	0.083	0.946	0.417	0.067
Acutalibacter	0.278	0.624	0.579	0.299	0.665	0.001	0.428	0.025	0.464	0.386	0.636	0.842	0.308	0.554	0.64	0.482	0.173	0.324	0.111	0.355	0.272	0.158	0.622	0.665	0.59	0.367	0.761
Caproiciproducens	0.923	0.496	0.218	0.202	0.004	0.822	0.979	0.637	0.618	0.303	0.149	0.228	0.153	0.469	0.282	0.305	0.758	0.973	0.316	0.18	0.847	0.395	0.383	0.905	0.956	0.106	0.517
Solibaculum	0.108	0.076	0.411	0.453	0.336	0.423	0.167	0.328	0.479	0.927	0.874	0.651	0.962	0.96	0.555	0.711	0.373	0.626	0.668	0.064	0.028	0.045	0.598	0.841	0.746	0.145	0.175
Eubacterium_g8	0.032	0.649	0.626	0.606	0.472	0.084	0.59	0.16	0.315	0.232	0.91	0.617	0.832	0.45	0.49	0.34	0.469	0.925	0.703	0.163	0.725	0.764	0.787	0.947	0.45	0.645	0.948
Anaerobutyricum	0.174	0.542	0.692	0.305	0.06	0.306	0.653	0.246	0.219	0.359	0.953	0.982	0.819	0.199	0.11	0.141	0.354	0.717	0.801	0.267	0.391	0.1	0.58	0.263	0.602	0.714	0.012
Eubacterium_g17	0.006	0.447	0.769	0.733	0.849	0.272	0.167	0.211	0.599	0.636	0.056	0.805	0.469	0.243	0.146	0.917	0.712	0.704	0.807	0.921	0.227	0.021	0.495	0.882	0.003	0.815	0.685
Eubacterium_g24	0.676	0.092	0.354	0.702	0.417	0.152	0.819	0.182	0.953	0.209	0.66	0.397	0.366	0.034	0.121	0.289	0.062	0.414	0.917	0.813	0.743	0.385	0.026	0.469	0.542	0.608	0.815
Howardella	0.478	0.635	0.775	0.136	0.286	0.858	0.932	0.414	0.743	0.203	0.904	0.993	0.141	0.396	0.771	0.128	0.371	0.038	0.008	0.761	0.576	0.41	0.745	0.726	0.757	0.867	0.512
Lachnospira	0.396	0.753	0.756	0.922	0.112	0.336	0.082	0.319	0.476	0.947	0.311	0.825	0.389	0.998	0.091	0.964	0.099	0.427	0.667	0.994	0.757	0.944	0.115	0.173	0.612	0.009	0.105
Anaerostipes	0.568	0.601	0.975	0.681	0.207	0.15	0.391	0.163	0.248	0.876	0.4	0.28	0.473	0.75	0.594	0.879	0.54	0.462	0.324	0.108	0.765	0.629	0.264	0.227	0.24	0.453	0.015
Frisingicoccus	0.16	0.269	0.938	0.991	0.032	0.179	0.13	0.25	0.107	0.934	0.414	0.933	0.737	0.379	0.09	0.968	0.341	0.771	0.549	0.544	0.266	0.238	0.085	0.238	0.318	0.881	0.816
Catenibacillus	0.093	0.438	0.151	0.093	0.116	0.172	0.335	0.195	0.535	0.63	0.124	0.119	0.959	0.57	0.047	0.178	0.086	0.309	0.569	0.089	0.195	0.086	0.152	0.156	0.041	0.125	0.918
Eubacterium_g4	0.347	0.998	0.614	0.655	0.882	0.952	0.334	0.944	0.596	0.018	0.871	0.278	0.288	0.083	0.171	0.03	0.727	0.936	0.705	0.945	0.047	0.023	0.435	0.65	0.619	0.789	0.891
Eisenbergiella	0.999	0.1	0.835	0.127	0.502	0.917	0.632	0.819	0.501	0.559	0.31	0.754	0.454	0.761	0.001	0.691	0.569	0.367	0.603	0.708	0.188	0.243	0.556	0.964	0.659	0.9	0.83
Acetitomaculum	0.972	0.026	0.744	0.607	0.46	0.463	0.193	0.471	0.95	0.353	0.367	0.834	0.561	0.829	0.555	0.603	0.298	0.679	0.538	0.53	0.44	0.182	0.254	0.223	0.513	0.237	0.274
Roseburia	0.388	0.233	0.022	0.512	0.021	0.067	0.856	0.152	0.757	0.019	0.291	0.021	0.873	0.839	0.037	0.015	0.173	0.588	0.444	0.3	0.593	0.63	0.542	0.704	0.33	0.218	0.234
Eubacterium_g21	0.369	0.67	0.471	0.039	0.001	0.953	0.787	0.789	0.109	0.04	0.097	0.243	0.523	0.341	0.072	0.051	0.353	0.587	0.983	0.784	0.594	0.644	0.043	0.45	0.153	0.485	0.786
Diplocloster	0.484	0.3	0.522	0.61	0.97	0.044	0.022	0.851	0.02	0.024	0.679	0.47	0.044	0.656	0.398	0.048	0.053	0.454	0.978	0.383	0.049	0.034	0.607	0.55	0.815	0.305	0.834
Coprococcus_g2	0.328	0.349	0.742	0.419	0.431	0.301	0.809	0.483	0.433	0.486	0.144	0.433	0.691	0.97	0.481	0.281	0.821	0.877	0.149	0.985	0.07	0.076	0.145	0.023	0.228	0.536	0.405
Mediterraneibacter	0.023	0.714	0.8	0.444	0.865	0.201	0.638	0.127	0.337	0.823	0.556	0.89	0.51	0.769	0.347	0.812	0.186	0.045	0.04	0.32	0.564	0.937	0.159	0.649	0.075	0.526	0.932
Oribacterium	0.621	0.896	0.988	0.245	0.763	0.794	0.834	0.777	0.551	0.335	0.64	0.802	0.314	0.877	0.256	0.406	0.098	0.353	0.885	0.201	0.501	0.866	0.003	0.001	0.263	0.103	0.358
Moryella	0.446	0.868	0.408	0.755	0.365	0.474	0.134	0.451	0.439	0.044	0.498	0.343	0.294	0.381	0.398	0.105	0.638	0.297	0.576	0.049	0.599	0.642	0.084	0.053	0.643	0.057	0.299
Porcincola	0.21	0.671	0.825	0.022	0.886	0.351	0.165	0.444	0.796	0.054	0.426	0.56	0.094	0.619	0.467	0.054	0.611	0.448	0.047	0.212	0.913	0.383	0.491	0.897	0.914	0.256	0.386
Faecalibacterium	0.228	0.497	0.03	0.099	1.14E-05	0.317	0.395	0.412	0.949	0.295	0.856	0.004	0.589	0.866	0.06	0.201	0.208	0.9	0.691	0.239	0.908	0.373	0.416	0.685	0.936	0.014	0.062
Gemmiger	0.59	0.932	0.289	0.349	0.028	0.813	0.206	0.937	0.415	0.075	0.733	0.039	0.568	0.688	0.031	0.038	0.972	0.657	0.374	0.806	0.869	0.735	0.219	0.713	0.142	0.238	0.57
Ruthenibacterium	0.722	0.682	0.283	0.336	0.221	0.028	0.863	0.141	0.375	0.332	0.474	0.168	0.846	0.72	0.763	0.149	0.886	0.273	0.327	0.58	0.943	0.85	0.605	0.912	0.057	0.086	0.506
Anaerofilum	0.994	0.901	0.399	0.207	0.231	0.318	0.436	0.232	0.788	0.639	0.332	0.08	0.235	0.739	0.555	0.83	0.274	0.404	0.188	0.392	0.763	0.318	0.041	0.259	0.425	0.442	0.883
Clostridium_g35	0.122	0.407	0.024	0.919	0.403	0.734	0.149	0.586	0.946	0.871	0.974	0.029	0.845	0.15	0.788	0.885	0.741	0.243	0.413	0.489	0.899	0.779	0.038	0.117	0.41	0.85	0.693
Butyrivibrio_g1	0.026	0.004	0.095	0.258	0.501	0.593	0.647	0.535	0.909	0.649	0.149	0.214	0.351	0.838	0.766	0.809	0.616	0.139	0.23	0.62	0.299	0.313	0.664	0.464	0.319	0.412	0.786
Blautia	0.053	0.743	0.416	0.176	0.396	0.628	0.424	0.405	0.72	0.755	0.131	0.767	0.413	0.702	0.841	0.913	0.892	0.815	0.667	0.021	0.65	0.326	0.614	0.558	0.129	0.42	0.744
Dorea	0.003	0.74	0.067	0.332	0.815	0.29	0.068	0.476	0.554	0.432	0.527	0.152	0.398	0.055	0.245	0.267	0.697	0.913	0.741	0.033	0.211	0.923	0.877	0.796	0.446	0.22	0.753
Sellimonas	0.193	0.384	0.531	0.131	0.012	0.272	0.785	0.367	0.821	0.923	0.454	0.604	0.676	0.962	0.116	0.899	0.371	0.346	0.532	0.439	0.673	0.717	0.349	0.438	0.191	0.748	0.032
Parabacteroides	0.741	0.138	0.984	0.809	0.56	0.564	0.926	0.425	0.571	0.856	0.873	0.505	0.459	0.097	0.946	0.417	0.398	0.69	0.754	0.842	0.231	0.442	0.037	0.346	0.522	0.421	0.613
Dysgonomonas	0.234	0.134	0.807	0.881	0.979	0.107	0.398	0.166	0.632	0.054	0.163	0.93	0.289	0.704	0.555	0.048	0.478	0.474	0.84	0.257	0.171	0.361	0.105	0.107	0.055	0.11	0.183
Barnesiella	1	0.3	0.22	0.303	0.279	0.671	0.955	0.709	0.135	0.477	0.288	0.885	0.979	0.937	0.561	0.588	0.99	0.017	0.14	0.678	0.425	0.428	0.073	0.214	0.525	0.066	0.891
Sodaliphilus	0.401	0.759	0.044	0.658	0.797	0.702	0.616	0.493	0.22	0.11	0.644	0.232	0.701	0.529	0.193	0.238	0.977	0.447	0.077	0.969	0.902	0.935	0.213	0.219	0.037	0.116	0.126
Porphyromonas	0.075	0.693	0.018	0.197	0.992	0.32	0.87	0.215	0.626	0.192	0.469	0.077	0.744	0.587	0.703	0.45	0.288	0.046	0.061	0.309	0.245	0.374	0.13	0.808	0.139	0.395	0.038
Odoribacter	0.1	0.72	0.412	0.294	0.783	0.934	0.827	0.642	0.25	0.268	0.356	0.756	0.09	0.748	0.049	0.671	0.826	0.751	0.462	0.769	0.243	0.03	0.007	0.007	0.151	0.454	0.585
Gabonibacter	0.657	0.273	0.327	0.822	0.239	0.452	0.145	0.886	0.218	0.543	0.814	0.693	0.517	0.444	0.859	0.976	0.325	0.514	0.347	0.804	0.744	0.527	0.031	0.097	0.372	0.329	0.043
Phocaeicola	0.767	0.544	0.441	0.324	0.004	0.295	0.211	0.113	0.029	0.494	0.405	0.244	0.052	0.385	0.675	0.469	0.26	0.166	0.073	0.352	0.309	0.944	0.023	0.089	0.279	0.973	0.727
Bacteroides	0.115	0.972	0.93	0.597	0.144	0.902	0.04	0.549	0.023	0.412	0.968	0.507	0.128	0.971	0.801	0.84	0.083	0.89	0.946	0.278	0.408	0.482	0.477	0.447	0.201	0.893	0.9
Jilunia	0.418	0.888	0.477	0.856	0.108	0.241	0.248	0.298	0.046	0.485	0.547	0.369	0.146	0.044	0.555	0.758	0.023	0.085	0.088	0.87	0.232	0.061	0.032	0.022	0.342	0.541	0.883

Supplementary Table 5. Corresponding *p*-values for the correlation matrix between IS gut microbiome taxa and clinical parameters

Corresponding *p*-values for Spearman correlations between genus-level gut microbial taxa and clinical parameters in IS are displayed. This table provides the numerical data for the Supplementary Figure 2.

Parameter	Description	Significance
Demographics and anthropometrics	Age<br>BMI<br>Height<br>Weight	Baseline physical profile of the patient
Stroke and neurological scales	Discharge MRS<br>Discharge NIHSS<br>Fazekas Scale<br>Initial NIHSS<br>Pre-stroke MRS	Severity of a stroke and level of disability
Metabolic and lipid profile	Fasting Blood Sugar<br>HDL<br>Hemoglobin A1c<br>Initial Glucose<br>LDL<br>Total Cholesterol<br>Triglycerides	Assess blood sugar control and the risk of atherosclerosis
Renal, hematology and inflammation	BUN<br>Creatinine<br>D-Dimer<br>Hematocrit<br>hsCRP<br>Platelet Count<br>PT	Kidney function, blood clotting ability, and general inflammation
Cardiovascular	DBP<br>SBP	Measurements of cardiovascular stability

Supplementary Table 6. Clinical parameters included in the correlation analysis and their significance.

Clinical parameters included in the correlation analysis are categorized and described according to their clinical relevance, including demographics, neurological scales, metabolic and lipid profiles, renal and inflammatory markers, and cardiovascular measures, along with their clinical significance.
