# Supplementary material for: Subtype-associated gut microbial taxa and their clinical correlates in acute ischemic stroke: comparison of large-artery atherosclerosis and small-vessel occlusion
Source: Front Microbiol. 2026 Sep 11;17:1867057. doi: 10.3389/fmicb.2026.1867057 (PMC13613636; doi:10.3389/fmicb.2026.1867057)

Subtype-Associated Gut Microbial Taxa and Their Clinical Correlates in Acute Ischemic Stroke: Comparison of Large Artery Atherosclerosis and Small Vessel Occlusion

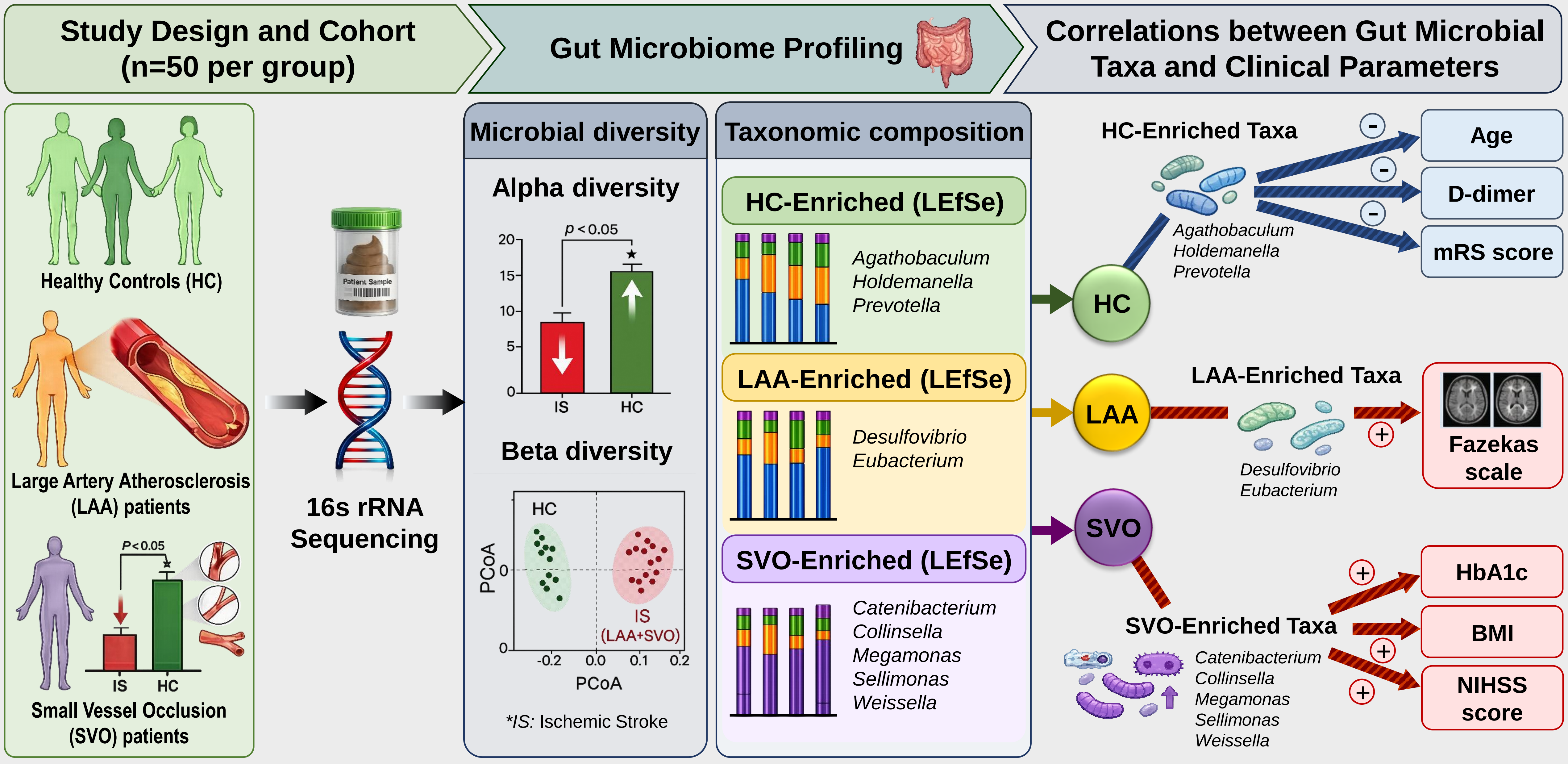

Supplement: Supplementary file 3 [file Data_Sheet_4.pdf]
